# Effect of cerebellar stimulation on awareness recovery in disorders of consciousness (CARE-DoC): A randomized, sham-controlled, crossover clinical trial

**DOI:** 10.1016/j.neurot.2025.e00635

**Published:** 2025-07-05

**Authors:** Rong Chen, Qiong Gao, Dian-Wei Wu, Jianmin Hao, Jing-Jing Zhao, Xuan Wang, Ji-Heng He, Fang Yuan, Xiao-Gang Kang, Ling Wang, Hai-Bo Di, Chang-Geng Song, Wen Jiang

**Affiliations:** a Department of Neurology, Xijing Hospital, Fourth Military Medical University, Xi'an, China; b State Key Laboratory of Traditional Chinese Medicine Syndrome/Department of Neurocritical Care, The Second Affiliated Hospital of Guangzhou University of Chinese Medicine, Guangdong Provincial Academy of Chinese Medical Sciences, Guangzhou, China; c Department of Health Statistics, Fourth Military Medical University, Xi'an, China; d International Unresponsive Wakefulness Syndrome and Consciousness Science Institute, Hangzhou Normal University, Hangzhou, China

**Keywords:** Transcranial magnetic stimulation, Intermittent theta burst stimulation, Disorders of consciousness, Cerebellum, Randomized crossover clinical trial

## Abstract

Disorders of consciousness (DoC) are major clinical challenges. We aimed to evaluate the effects of cerebellar intermittent theta-burst stimulation (CRB-iTBS) in the treatment of DoC. We conducted a randomized, sham-controlled, double-blind, cross-over clinical trial. Patients with vegetative state/unresponsive wakefulness syndrome or minimally conscious state within 15 days to 1 year after brain injuries were recruited. The bilateral cerebellum was targeted by iTBS for 5 consecutive days under neuronavigation. The primary outcome was the change in Coma Recovery Scale-Revised (CRS-R) total scores after five sessions. Secondary outcomes included changes in CRS-R scores after the first session, the changes in CRS-R subscales and the alterations in “ABCD” EEG patterns after the first and fifth sessions. Follow-up outcomes included six-month functional outcomes and consciousness recovery. We included 44 patients in the intention-to-treat analysis. No significant difference was observed in the change of CRS-R total scores between active and sham groups after five sessions (difference ​= ​0.428, 95 ​% CI ​= ​−0.202 - 1.057, *P* ​= ​0.180). However, active stimulation induced greater CRS-R improvements after the first session (difference ​= ​1.048, 95 ​% CI ​= ​0.480–1.615, *P* ​< ​0.001), especially in auditory, visual, oromotor/verbal, and arousal subscales. Active stimulation increased the prevalence of EEG patterns “C” and “D” after both the first and fifth sessions. Favorable six-month functional outcomes and consciousness recovery were associated with an elevation in “ABCD” EEG patterns during active treatment periods. These findings demonstrate that CRB-iTBS exhibits potential as a neuromodulation strategy to promote consciousness recovery in DoC.

## Introduction

Advances in intensive care have increased survival rates following severe brain injuries [1]. However, this progress has led to a greater incidence of disorders of consciousness (DoC), such as vegetative state/unresponsive wakefulness syndrome (VS/UWS) and minimally conscious state (MCS) [2]. DoC impose severe burdens on patients, their families, and society. Currently, effective treatments for DoC remain limited. Among existing therapies, only amantadine has been shown to significantly enhance behavioral recovery in severe traumatic brain injury patients with DoC in the subacute phase [3]. This highlights the urgent need for new and effective treatments.

Transcranial magnetic stimulation (TMS) is a non-invasive technique that modulates neural excitability and it has shown potential in the treatment of DoC [4]. Previous randomized controlled trials (RCTs) have used high-frequency repetitive TMS (HF-rTMS) targeting regions such as the motor cortex or dorsolateral prefrontal cortex (DLPFC); however, the results have been inconsistent [[5], [6], [7], [8], [9], [10]]. These disparities may stem from differences in study designs, stimulation parameters, and the selection of brain regions for stimulation. Thus, it's crucial to optimize stimulation paradigms and identify more effective stimulation targets for improving therapeutic outcomes.

The integrity of the thalamocortical circuit is crucial for consciousness recovery in DoC [4]. The cerebellum, traditionally associated with motor control, also plays a vital role in higher-order brain functions such as emotion and cognition [11]. It has excitatory connections to the thalamus, which projects to cortical areas, including the frontal and parietal regions [12]. This cerebello-thalamocortical loop offers a promising pathway for promoting consciousness recovery. Preliminary study in patients with MCS demonstrated that cerebellar transcranial direct current stimulation (tDCS) increased EEG power in theta and gamma frequencies in the frontal and parietal cortices, which was associated with temporary consciousness improvements [13]. Additionally, animal studies have demonstrated that activating cerebellar Purkinje cells enhances cortical activity and facilitates consciousness recovery from anesthesia [14].

Building on these findings, our study employs intermittent theta-burst stimulation (iTBS), a potent excitatory TMS protocol [15], targeted to the cerebellar lobule VII under neuronavigation. The lobule VII of the cerebellar cortex has close neural connections with the frontoparietal network, which is associated with consciousness [11,12,16,17], suggesting that stimulation of this region may potently modulate frontoparietal network activity. We investigated the efficacy and safety of cerebellar iTBS (CRB-iTBS) in treating DoC, aiming to provide a new therapeutic avenue for this challenging condition.

## Materials and methods

### Study design and participants

This study was a randomized, sham-controlled, double-blinded, cross-over clinical trial conducted at the Coma Awakening Center, Department of Neurology, Xijing Hospital, Xi'an, China. The complete protocol is detailed in the Supplementary protocol file. This study was in accordance with the Declaration of Helsinki and the recommendation of Good Clinical Practice guidelines. This study was registered at Clinicaltrials.gov (NCT05558930) and was approved by the ethics committee of Xijing Hospital (KY20222028–F-1). Written informed consents were acquired from legal surrogates.

To recruit patients with DoC, we posted recruitment advertisements on the official website of Xijing Hospital, clearly outlining the inclusion and exclusion criteria of our study. Over the entire study period, a total of 101 DoC patients underwent preliminary screening. These patients were recruited from diverse settings, including rehabilitation centers, neurological intensive care units (NICUs), home care settings, and Xijing Hospital Emergency Department. All participants were subsequently transferred to the Coma Awakening Center of our hospital for a standardized evaluation by a senior neurologist based on the inclusion and exclusion criteria. Inclusion criteria were: (1) age ≥18 years; (2) diagnose of VS/UWS or MCS based on at least two Coma Recovery Scale-Revised (CRS-R) assessments; (3) time since brain injury from 15 days to 1 year; (4) written informed consent acquired from legal surrogates. Exclusion criteria included: (1) brain injury of unknown etiology; (2) pre-existing mental or psychiatric disorders prior to brain injury; (3) uncontrolled seizures or status epilepticus; (4) unstable or deteriorating medical conditions; (5) skull defect; (6) contraindications to the magnetic resonance imaging (MRI); and (7) contraindications to TMS [18].

### Randomization and blinding

Patients were randomized 1:1 into one of two groups: active stimulation followed by sham stimulation (active stimulation-first group) or vice versa (sham stimulation-first group). Randomization was performed using a random number table without stratification or block randomization. The randomization codes were generated by an unmasked statistician who was not involved in this study. Group allocation was blinded to all patients and investigators, except for the TMS operator, who did not participate in follow-up or data analysis [19].

### Crossover procedures

**Fig. 1:**
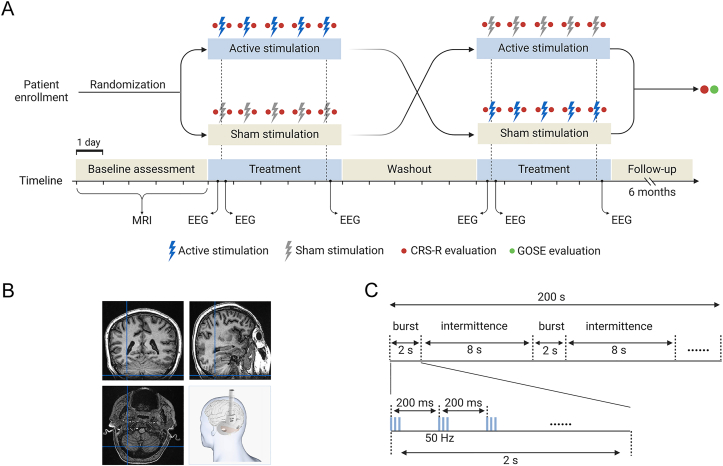
Study design. (A) A timeline summarizing the trial protocol. During the baseline assessment period, clinical evaluations and magnetic resonance imaging (MRI) were performed. Patients were then randomly assigned to receive either active cerebellar intermittent theta-burst stimulation (CRB-iTBS) or sham stimulation. After a five-day washout period, they crossed over to the alternate treatments. Each treatment period consisted of five consecutive days of daily CRB-iTBS or sham treatment session. Electroencephalogram (EEG) recordings were conducted within 30 ​min before and after the first, and after the fifth treatment sessions. The Coma Recovery Scale-Revised (CRS-R) was assessed within 2 ​h before and after the treatment sessions. (B) CRB-iTBS was administered over the cerebellum using a double-cone coil under neuronavigation guidance. **(C)** A schematic diagram illustrating the iTBS stimulation pattern.

The study design is illustrated in Fig. 1. After a 5-day baseline assessment, patients were randomly assigned to either the active stimulation-first group or the sham stimulation-first group. During each treatment period, patients received either active CRB-iTBS stimulation or sham stimulation once daily for 5 consecutive days. The two treatment periods were separated by a 5-day washout period. Demographic, MRI, and clinical characteristics of the patients were collected at enrollment. The level of consciousness was assessed with CRS-R before and after each stimulation session within 2 ​h. EEG was continuously recorded for 20 ​min before and after the first and after the fifth treatment sessions within 30 ​min of the stimulation (Fig. 1A).

### TMS stimulation protocol

We delivered the CRB-iTBS by targeting to the VII lobule of cerebellum under neuronavigation [20]. Individual structural MRI data were uploaded to a neuronavigation system (Quicks Vision, Yingchi, Shenzhen, China) to localize the coil over the VII lobule of cerebellum according to MRI atlas (Fig. 1B) [21]. CRB-iTBS was delivered with a double-cone coil in 90 ​mm diameter connected to the TMS device (M ​− ​100 Ultimate, Yingchi, Shenzhen, China). The iTBS protocol consisted of 3 pulses at 50 ​Hz repeated at 5 ​Hz, with a 2 ​s train repeated every 10 ​s for a total of 200 ​s (600 pulses) (Fig. 1C) [22]. During each treatment session, the left and right hemispheres of the cerebellum were sequentially stimulated, separated by 5-min interval. Sham stimulation was delivered by rotating the coil 90° from the scalp over the targeting position according to previous studies [[23], [24], [25], [26], [27]]. All patients wore earplugs during stimulation to block out the auditory interference. The active CRB-iTBS was delivered at an intensity of 90 ​% resting motor threshold (RMT) of the contralateral primary motor cortex adjusted according to the individual scalp-to-cortex distance [28]. For patients with only one side of RMT detectable, the intensity of iTBS was based on this detectable RMT. For patients with undetectable RMTs on both sides, 50 ​% of the maximum stimulator output was used, as previously described [29].

### Behavioral assessment

Two qualified and independent neurologists who were blinded to the allocation evaluated the CRS-R scores to assess the level of consciousness [30]. Diagnoses of VS/UWS and MCS (minus or plus) were determined based on the presence of specific CRS-R subscale items [31]. The baseline consciousness was determined by the highest CRS-R score from two assessments conducted one day apart during the baseline assessment period.

### EEG recording and analysis

EEG signals were recorded using 32 Ag/AgCl ring electrodes connected to an amplifier system (BrainAmp MR Plus, Brain Products GmbH, Gilching, Germany). The electrodes were positioned according to the international 10–20 system. The EEG data were acquired at a sampling rate of 1000 ​Hz, with impedance kept below 5 ​kΩ. EEG were continuously recorded for 20 ​min within 30 ​min before and after the first stimulation session, as well as after the fifth stimulation session (Fig. 1A). Patients were awake with their eyes open during EEG recordings, and an arousal-promoting protocol was implemented if their eyes were closed [30].

To evaluate the neurophysiological effects of CRB-iTBS, an “ABCD” EEG pattern analysis was conducted based on power spectral density across different frequency bands, which decodes the integrity of thalamocortical circuitry and the preservation of consciousness [2]. The “ABCD” EEG patterns are defined as follows: (1) pattern A indicates a complete loss of thalamocortical integrity, with the EEG power spectrum restricted to ​< ​4 ​Hz; (2) pattern B reflects a narrow oscillation of the layer V pyramidal cells in the theta frequency range (4–7 ​Hz), resulting from the depolarization of neocortical neurons with depressed membrane potentials; (3) pattern C represents oscillations in theta and beta frequencies due to the partial restoration of neocortical membrane potentials and coincident bursts of the deafferented thalamic neurons; (4) pattern D refers to a normal neocortical neuronal firing pattern at alpha and beta frequencies (Fig. S1). The EEG responsiveness to CRB-iTBS was analyzed. Responders were defined as patients who exhibited elevated levels of “ABCD” EEG patterns compared to the baseline after either the first or fifth active stimulation session. Two qualified EEG technicians analyzed the EEG independently, and disagreement was resolved by consulting a third EEG technician.

### Basic treatment and routine rehabilitation

Sedative medications and the medications potentially interfering with brain stimulation, such as Na^+^/Ca^2+^ channel blockers or N-Methyl-d-Aspartate receptor antagonists were suspended from baseline assessment until the last EEG and behavior assessments to avoid interference with the evaluation of EEG and consciousness [32,33]. Other medications, physiotherapy, and rehabilitation were kept unchanged throughout the experiment [33].

### Outcomes

The primary outcome was the difference in the change of CRS-R total score between the active and sham stimulation groups after five treatment sessions (the change of CRS-R total score meant the change between the CRS-R total score collected before the first treatment session, and the CRS-R total score collected after five treatment sessions). The prespecified secondary outcomes were: (1) the between-group difference in the change of CRS-R subscale scores after five treatment sessions (the change of CRS-R subscale scores meant the change between the CRS-R subscale scores collected before the first treatment session, and the CRS-R subscale scores collected after five treatment sessions); (2) the difference in ABCD EEG pattern proportions between active and sham stimulations after treatment on the fifth day. The post hoc secondary outcomes were: (1) the between-group difference in the change of CRS-R total and subscale scores after the first treatment session (the change of CRS-R scores meant the change between the CRS-R scores collected before the first treatment session, and the CRS-R scores collected after the first treatment session); (2) the difference in ABCD EEG pattern proportions between active and sham stimulations after treatment on the first day.

We evaluated the functional outcome and the consciousness recovery of the patients with DoC three months and six months post-treatment via structured telephone interviews by a trained interviewer, either with patients themselves or close relatives. The functional outcome was assessed using the Glasgow Outcome Scale-Extended (GOSE), with a score of ≥4 indicating a favorable outcome and a score of <4 indicating an unfavorable outcome [34]. The levels of consciousness were evaluated via a structured telephone interview specifically designed to capture key consciousness-related behaviors, feasible for remote evaluation, according to previously published studies [32,35,36]. Patients were categorized into the improved consciousness group if they transitioned from UWS to MCS or to emergence from MCS (EMCS), from MCS minus to MCS plus or to EMCS, and from MCS plus to EMCS. Patients with unimproved consciousness were defined as those with a reduced or unchanged level of consciousness. The consciousness status of patients who died during the follow-up period was evaluated based on their level of consciousness prior to death. Specifically, for patients who died 6 months post-treatment, their pre-death consciousness was assessed according to their condition at 3 months post-treatment. For those who died within 3 months of treatment, their pre-death consciousness was evaluated based on their status at hospital discharge.

### Statistical analyses

We determined that enrolling 44 patients would provide 90 ​% power to detect a mean difference of 1.2 points (standard deviation [SD] ​= ​1.5) in CRS-R changes between the active and sham stimulation groups, using a two-sided α of 0.05 and accounting for a 15 ​% dropout rate. The anticipated 1.2 ​± ​1.5-point difference was conservatively estimated based on previous studies [5,7,9,33].

Continuous variables were expressed as mean ​± ​SD or median (interquartile range, IQR), and categorical variables were expressed as percentages. Baseline continuous variables were analyzed using Student's *t* - test for normal distribution, and Mann - Whitney *U* test for skewed distribution. Categorical variables were analyzed using χ^2^ test analysis and Fisher's exact tests, when appropriate.

The between-group differences in the change of CRS-R total and subscale scores after the first and fifth treatment sessions were analyzed using a linear mixed model (LMM) with fixed effects (sequence, period, time since injury, etiology, and age) and random effects (subjects for repeated measurements) [37,38]. The effects of CRB-iTBS on CRS-R total scores were further analyzed in subgroups of patients according to their baseline characteristics, i.e., VS/UWS and MCS, as well as anoxia etiology and non-anoxia eitology. The between-group differences in power spectral density (PSD) after the first and fifth treatment sessions were also analyzed via LMM, incorporating baseline PSD as a fixed effect, alongside other fixed and random variables as above. The cumulative LMM, which incorporated fixed and random effects as those of LMM (fixed effects: sequence, period, time since injury, etiology, and age; random effects: subjects for repeated measurements) and added baseline “ABCD” EEG patterns as fixed effects, was used to analyze the between-group differences in “ABCD” EEG patterns after the first and fifth treatments [39,40]. We used the ordinal logistic regression to explore the relationships between the baseline “ABCD” EEG patterns and the functional outcomes and recovery of consciousness six months post-treatment. To investigate the relationships between EEG responsiveness and six-month outcomes, we used the generalized linear model, with adjustments made for baseline characteristics and time period.

The analysis was conducted in the intention-to-treat (ITT) populations, and was repeated in the per-protocol (PP) populations to test the robustness. For the ITT population, missing data were imputed using the last observation carried forward method. EEG analyses were performed in patients with valid EEG data, and adjustments for multiple comparisons were conducted using the false discovery rate method. Two-sided *P*-values <0.05 were considered statistically significant. All statistical analyses were performed using PASS 20.0 (NCSS, LLC, Kaysville, UT, USA), R version 4.3.0 and SPSS version 26 (SPSS Inc., Chicago, IL, USA).

## Results

### Baseline demographic and clinical characteristics

**Fig. 2:**
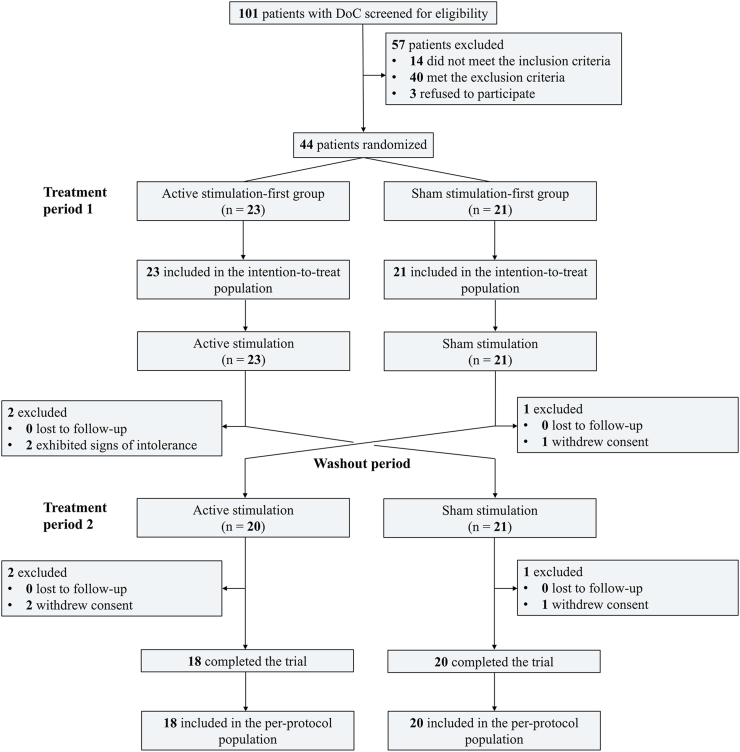
CONSORT flow diagram.

From April 29, 2022 to March 2, 2024, 101 DoC patients were screened, of whom 44 underwent randomization. Twenty-three patients were assigned to the active stimulation-first group and 21 to the sham stimulation-first group, and they were included in the ITT analysis. Six patients were excluded, including four who withdrew consent and two who exhibited signs of intolerance during active stimulation, leaving 20 and 18 patients from the active and sham stimulation-first groups in PP analysis, respectively (Fig. 2). The median (IQR) age of ITT patients was 53.0 (42.0–64.8) years, with 45.5 ​% (20 patients) being female. At enrollment, the median (IQR) time since brain injury was 48 (28.3–84.8) days, and the median (IQR) baseline CRS-R score was 6.5 (5.0–10.3) points. Of the 44 ITT patients, 22 (50 ​%) patients were in VS/UWS, while the remaining 22 were in MCS (Table 1, Table S1). Baseline characteristics for the PP population are detailed in Table S2.

**Table 1:** Baseline demographic and clinical characteristics of the intention-to-treat population.

	Total (n ​= ​44)	Active stimulation-first group (n ​= ​23)	Sham stimulation-first group (n ​= ​21)	*P* value
**Age, years**	53.0 (42.0–64.8)	56.0 (45.0–64.5)	49 (34–62)	0.372
**Sex**				0.123
Female	20 (45.5 ​%)	13 (56.5 ​%)	7 (33.3 ​%)	
Male	24 (54.5 ​%)	10 (43.5 ​%)	14 (66.7 ​%)	
**Time since injury**				
≤28 days	10 (25.0 ​%)	4 (17.4 ​%)	6 (28.6 ​%)	0.600
>28 days	34 (75.0 ​%)	19 (82.6 ​%)	15 (71.4 ​%)	
**Consciousness**				0.763
VS/UWS	22 (50.0 ​%)	12 (52.2 ​%)	10 (47.6 ​%)	
MCS	22 (50.0 ​%)	11 (47.8 ​%)	11 (52.4 ​%)	
**CRS-R score**				
Total	6.5 (5.0–10.3)	7.0 (5.0–12.0)	6.0 (5.0–9.0)	0.995
Auditory	1.0 (1.0–2.0)	1.0 (1.0–2.0)	1.0 (1.0–1.0)	0.727
Visual	0.0 (0.0–3.0)	0.0 (0.0–3.0)	0.0 (0.0–3.0)	0.928
Motor	2.0 (1.0–4.0)	2.0 (1.0–5.0)	2.0 (1.0–3.0)	0.932
Oromotor/verbal	1.0 (1.0–2.0)	1.0 (1.0–2.0)	1.0 (1.0–1.0)	0.785
Communication	0.0 (0.0–0.0)	0.0 (0.0–0.0)	0.0 (0.0–0.0)	0.538
Arousal	2.0 (1.0–2.0)	2.0 (1.0–2.0)	2.0 (1.0–2.0)	0.702
**Etiology**				0.166
Anoxia	18 (40.9 ​%)	10 (43.5 ​%)	8 (38.1 ​%)	
Stroke				
Hemorrhagic stroke	12 (27.3 ​%)	7 (30.4 ​%)	5 (23.8 ​%)	
Ischemic stroke	3 (6.8 ​%)	0 (0)	3 (14.3 ​%)	
TBI	7 (15.9 ​%)	2 (8.7 ​%)	5 (23.8 ​%)	
Other^a^	4 (9.1 ​%)	4 (17.4 ​%)	0 (0)	

Data are presented as n (%), mean (standard deviation), or median (interquartile range). VS/UWS, vegetative state/unresponsive wakefulness syndrome; MCS, minimally conscious state; CRS-R, Coma Recovery Scale-Revised; TBI, traumatic brain injury.

a Other etiology refers to osmotic demyelination syndrome.

### The effects of CRB-iTBS on consciousness and EEG

**Fig. 3:**
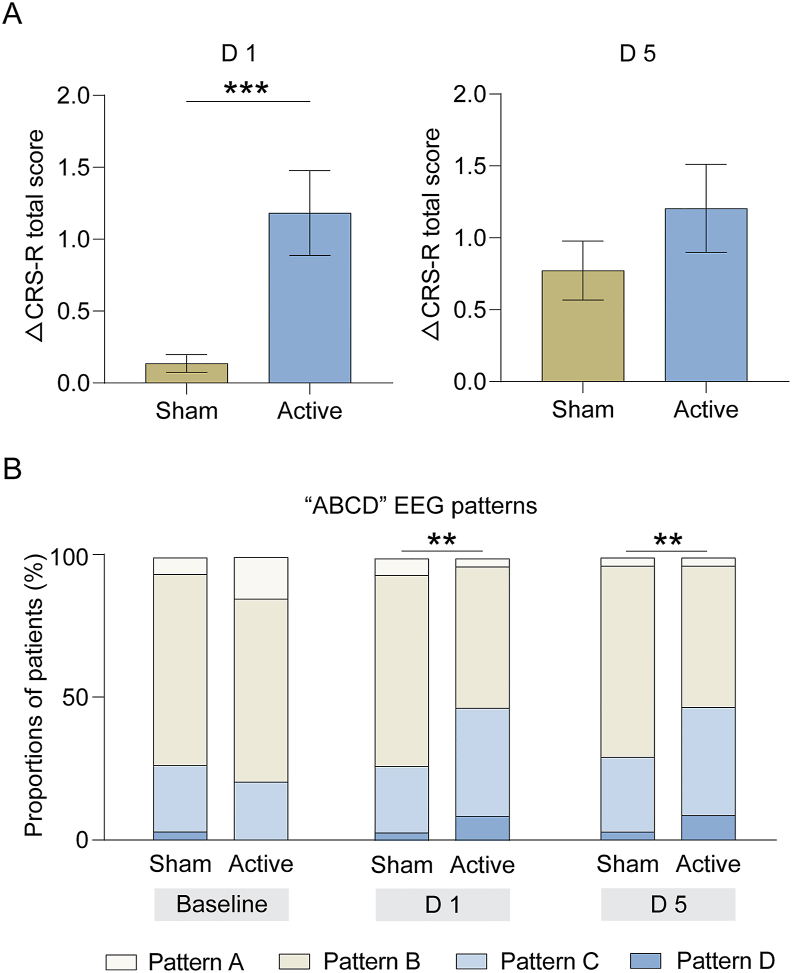
Clinical scores and “ABCD” EEG patterns. (A) The between-group differences in the change of CRS-R total scores after the first treatment session (D1) and after five treatment sessions (D5) in the intention-to-treat population. (B) The between-group differences in the distribution of “ABCD” EEG patterns at baseline, after the first treatment session (D1) and after five treatment sessions (D5). ∗∗*P* ​< ​0.01, ∗∗∗*P* ​< ​0.001, comparisons between the active and sham stimulation groups.

No significant differences were observed in the changes of CRS-R total scores between active and sham stimulation groups after five treatment sessions in both the ITT population (Fig. 3A; between-group difference after five treatment sessions ​= ​0.428, 95 ​% CI ​= ​−0.202 - 1.057, *P* ​= ​0.180) and the PP population (Fig. S2). However, active stimulation induced a significantly greater increase in the CRS-R total scores after the first treatment session compared to sham stimulation in both the ITT population (Fig. 3A; between-group difference after the first treatment session ​= ​1.048, 95 ​% CI ​= ​0.480–1.615, *P* ​< ​0.001) and the PP population (Fig. S2), especially in the auditory, visual, oromotor/verbal, and arousal subscales (Tables S3 and S4).

Subgroup analyses revealed that active stimulation was effective across all consciousness states (VS/UWS and MCS) and etiologies (anoxia and non-anoxia) after the first session, but not after five sessions (Figs. S3 and S4). Interestingly, non-anoxic patients exhibited a more pronounced improvement in CRS-R total scores compared to anoxic patients after the first treatment (between-group difference ​= ​1.18, 95 ​% CI: −0.11 - 2.47; *P* ​= ​0.074) (Fig. S4).

For EEG analysis, four patients in the PP population were excluded due to excessive artifacts, leaving 34 patients for evaluation. At baseline, the distribution of “ABCD” EEG patterns was similar between the active and sham groups (adjusted OR ​= ​0.60, 95 ​% CI ​= ​0.22–1.63, *P* ​= ​0.316; Fig. 3B). Active stimulation resulted in significantly different “ABCD” EEG pattern distributions after the first (adjusted OR ​= ​4.72, 95 ​% CI ​= ​1.62–13.77, *P* ​= ​0.004) and fifth treatment sessions (adjusted OR ​= ​3.30, 95 ​% CI ​= ​1.36–7.97, *P* ​= ​0.008; Fig. 3B), with more patients exhibiting patterns “C” and “D”. Active stimulation also reduced global PSD in the delta frequency after the first session, particularly in frontal, parietal, and occipital lobes (Figs. S5 and S6). These effects on PSD were not observed after five sessions.

### Long-term outcomes and safety considerations

**Fig. 4:**
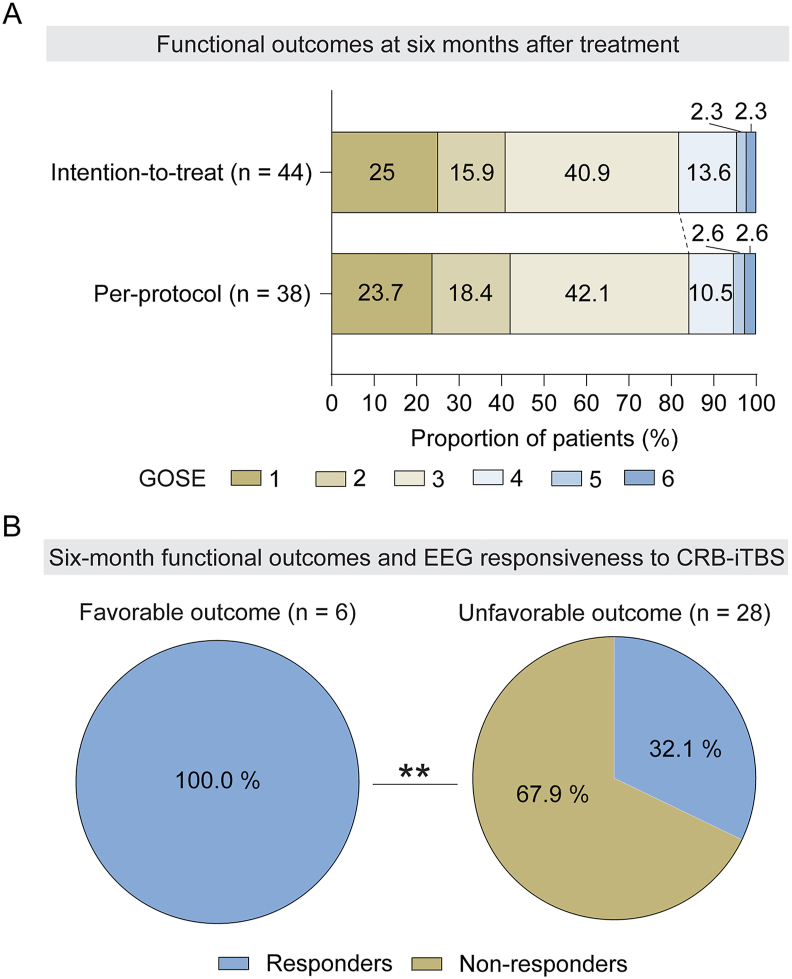
Long-term functional outcomes at six months after **treatment.** (A) Functional outcomes at six months post-treatment as assessed by the Glasgow Outcome Scale-Extended (GOSE) in the intention-to-treat population and the per-protocol population. A GOSE score of ≥4 was classified as a favorable outcome, while a score of <4 was considered an unfavorable outcome. (B) The relationship between six-month functional outcomes and EEG responsiveness to cerebellar intermittent theta-burst stimulation (CRB-iTBS). Responders were defined as patients who exhibited elevated levels of “ABCD” EEG patterns during active stimulation, either after the first or fifth treatment sessions. ∗∗*P* ​< ​0.01, comparison between the patients with favorable functional outcome and the unfavorable functional outcome.

Among the ITT population, 9.1 ​% (4 patients) had favorable functional outcomes and 50 ​% (22 patients) showed improved consciousness at three months post-treatment, and these proportions increased to 18.2 ​% (8 patients) and 59.1 ​% (26 patients) at six months post-treatment, respectively (Fig. 4A and Fig. S7A). Baseline “ABCD” EEG patterns were not associated with six-month functional outcomes (Table S5) but were significantly linked to consciousness recovery (Table S6). Furthermore, responders—patients who exhibited elevated “ABCD” EEG patterns during active stimulation—were more likely to achieve favorable outcomes. Among patients with favorable outcomes, 100 ​% were responders, compared to only 32.1 ​% of those with unfavorable outcomes (*P* ​= ​0.001; Fig. 4B). Similarly, responders were significantly more prevalent among patients with improved consciousness than those without (*P* ​= ​0.014; Fig. S7B). Two patients displayed signs of intolerance during active stimulation, such as painful facial expressions, moaning, crying, and head movements. During the six-month follow-up, 11 patients passed away. No other adverse or serious events occurred throughout the study.

## Discussion

The present study demonstrated that although the effect of CRB-iTBS on consciousness recovery was not sustained after five active stimulation sessions, the initial improvement observed following the first session underscored the potential of CRB-iTBS as an effective intervention for DoC with good safety profile. Notably, consciousness improvements were coincided with enhanced integrity of the thalamocortical circuit, as evidenced by elevated “ABCD” EEG patterns. Additionally, long-term outcomes were more favorable among patients who responded to CRB-iTBS.

The transient yet significant consciousness improvements induced by CRB-iTBS align with previous neuromodulation studies, including cerebellar tDCS [13], single-session rTMS targeting the DLPFC [41], and tDCS over the precuneus [42]. Several factors may account for this temporary efficacy. First, the baseline characteristics presented inherent challenges: half of the patients were in VS/UWS, a condition notoriously difficult to treat [33], and many had anoxic etiologies associated with extensive neuronal damage and poorer prognoses [43]. The greater CRS-R improvement noted in non-anoxic patients highlights the challenges of treating anoxic DoC with CRB-iTBS. Nonetheless, including patients with diverse etiologies reflects real-world clinical conditions and underscores the need for effective treatments for all DoC patients. Second, the diminished efficacy after five treatment sessions may stem from rapid neuroadaptive responses to CRB-iTBS. Third, the consciousness state of DoC patients exhibits spontaneous fluctuations. The spontaneous fluctuations in CRS-R total scores observed over the 5 days of treatment may have diminished the differences between real and sham stimulation. Future multi-center studies with large sample sizes and optimized stimulation protocols are warranted to validate the therapeutic effects of CRB-iTBS.

In the pathogenesis of DoC, the disruption of the thalamocortical circuit impairs consciousness [2]. The EEG findings suggested that CRB-iTBS enhanced thalamocortical circuit integrity. CRB-iTBS reduced the delta-frequency PSD in the frontal, parietal, and occipital lobes. Delta oscillation is associated with the hyperpolarization of cortical neurons, which usually occurs in sleep, anesthesia, and DoC [44]. The decreased power in delta oscillation indicated that CRB-iTBS restored the neuronal excitability. Furthermore, the increased prevalence of “C” and “D” EEG patterns following CRB-iTBS points to improved thalamocortical connectivity, which laid the foundation for consciousness recovery [2]. Notably, although changes in “ABCD” EEG patterns persisted after the fifth sessions, behavioral improvements were only observed after the first session. This discrepancy merits further investigation into the underlying mechanisms.

At six months post-treatment, 18.2 ​% of patients achieved favorable functional outcomes, and 59.1 ​% demonstrated improved consciousness, which surpass those for thalamic deep brain stimulation in DoC [45], underscoring the potential impact of CRB-iTBS. Notably, responders were more likely to achieve favorable long-term functional and consciousness outcomes. This suggests that responders may possess more intact neural structures, enabling CRB-iTBS to effectively facilitate the thalamocortical integrity. Identifying responders at an early stage could optimize the therapeutic application of CRB-iTBS, thereby maximizing patient benefit.

Unlike previous “top-down” strategies that targeted the DLPFC or motor cortex [6,8,10,46], this study highlights the cerebellum as a viable “bottom-up” target for consciousness recovery. The cerebellum's excitatory projections to the thalamus, which in turn modulates wide-ranging cortical areas [12], provide a unique pathway for influencing cortical activity. We focused CRB-iTBS on cerebellar lobule VII, which is closely linked to the frontoparietal network essential for consciousness [11]. Notably, the temporary consciousness recovery was related to increase in the scores of auditory, visual, oromotor/verbal, and arousal functions in CRS-R subscales, known to be involved multiple cortical and subcortical regions. This further suggested the divergent nature of cerebellum's downstream projection, which could be harnessed to modulate widespread brain areas.

During active stimulation, two patients displayed signs that might be interpreted as intolerance, such as painful facial expressions and head movements. However, these responses may also indicate heightened consciousness. Further research is necessary to clarify these observations.

Several limitations should be noted. First, although our study was conducted at a single center, the sample size of 44 patients was comparable to or exceeded that of other DoC studies [32,47,48]. Second, the diverse etiologies of patients mirror real-world clinical practice, enhancing the applicability of the findings, but may reduce statistical power. Third, we did not employ functional MRI or positron emission tomography due to high costs, logistical challenges, and the inability to ensure continuous monitoring, which would otherwise provide more detailed mechanism insights. Fourth, although the sham simulation provides comparable auditory stimulation compared to sham stimulation, it could not fully reproduce the somatosensory effects of active stimulation. This may confound treatment effects of the active CRB-iTBS and future studies with optimized sham coil that better replicates the somatosensory effects of active stimulation are needed. Fifth, we employed a structured telephone interview rather than the CRS-R scale to assess consciousness levels during follow-up. Although previous reports suggest that such structured telephone interviews can capture key consciousness-related behaviors and may be feasible for remote evaluation [32,35,36], it should be noted that this remains an unvalidated assessment method for consciousness. Further comparative studies between structured telephone interviews and standard clinical CRS-R assessments are warranted to rigorously evaluate the reliability of this telephone-based approach.

In conclusion, CRB-iTBS was not able to promote consciousness recovery after five daily treatments, although it temporarily facilitated consciousness after the first treatment and enhanced the integrity of the thalamocortical circuit. Future studies employing optimized stimulation protocols are warranted to explore the cerebellum's potential as a “bottom-up” stimulation target to treat DoC.

## Ethics approval and consent to participate

This study was approved by the ethics committee of Xijing Hospital (KY20222028–F-1). Written informed consents were acquired from legal surrogates.

## Availability of data and materials

The datasets used and/or analyzed during the current study are available from the corresponding author on reasonable request.

## Author contributions

WJ conceived and designed the study, provided the main administrative, technical, and material support. CGS provided the administrative, technical, and material support, and contributed to the study design. RC contributed to study design, enrollment of patients, data collection, and integrity of the data. QG and DWW participated in study design, enrollment of patients, data collection and interpretation, and statistical analysis of data. JMH, JJZ, XW, FY, XGK, LW and HBD gave support of data collection, statistical analysis, and interpretation of the data. JHH contributed to TMS treatment. RC, QG, DWW, CGS, and WJ drafted the manuscript with input from all the authors and with no external writing assistance. All authors have critically reviewed and approved the final version of the manuscript.

## Funding

WJ reports grants from the National Natural Science Foundation of China (82441054), Shaanxi Province Special Support Program for Leading Talents in Scientific and Technological Innovation (tzjhjw), and Clinical Research Project of the Fourth Military Medical University (2023LC2314).

## Declaration of competing interest

The authors declare that they have no competing interests. The funder had no role in the design of the study or the decision to publish the results.
