# Supplementary material for: Effect of cerebellar stimulation on awareness recovery in disorders of consciousness (CARE-DoC): A randomized, sham-controlled, crossover clinical trial
Source: Neurotherapeutics. 2025 Jul 5;22(5):e00635. doi: 10.1016/j.neurot.2025.e00635 (PMC12491794; doi:10.1016/j.neurot.2025.e00635)
Supplement: Multimedia component 1 [file mmc1.pdf]

Supplementary Materials for CARE-DoC

Table of contents

Supplementary Figures **2**

Figure S1. The "ABCD" EEG patterns reflecting the thalamocortical circuit integrity. 2

Figure S2. The changes of CRS-R total scores in the per-protocol population. 3

Figure S3. Subgroup analysis of the change of CRS-R total scores in the intention-to-treat population. 4

Figure S4. Subgroup analysis of the change of CRS-R total scores in the per-protocol population. 5

Figure S5. The global power spectral density in patients with DoC. 6

Figure S6. The topographic power spectral density in patients with DoC. 8

Figure S7. Long-term consciousness recovery of patients receiving CRB-iTBS. 9

Supplementary Tables **10**

Table S1. Detailed clinical and demographic data of the enrolled patients. 10

Table S2. Baseline demographic and clinical characteristics of the per-protocol population. 16

Table S3. Changes in Coma Recovery Scale-Revised subscale scores in the intention-to-treat population. 17

Table S4. Changes in Coma Recovery Scale-Revised subscale scores in the per-protocol population. 18

Table S5. Baseline "ABCD" EEG patterns of patients with favorable and unfavorable functional outcomes 19

Table S6. Baseline "ABCD" EEG patterns of patients with improved and unimproved consciousness 20

Supplementary Figures

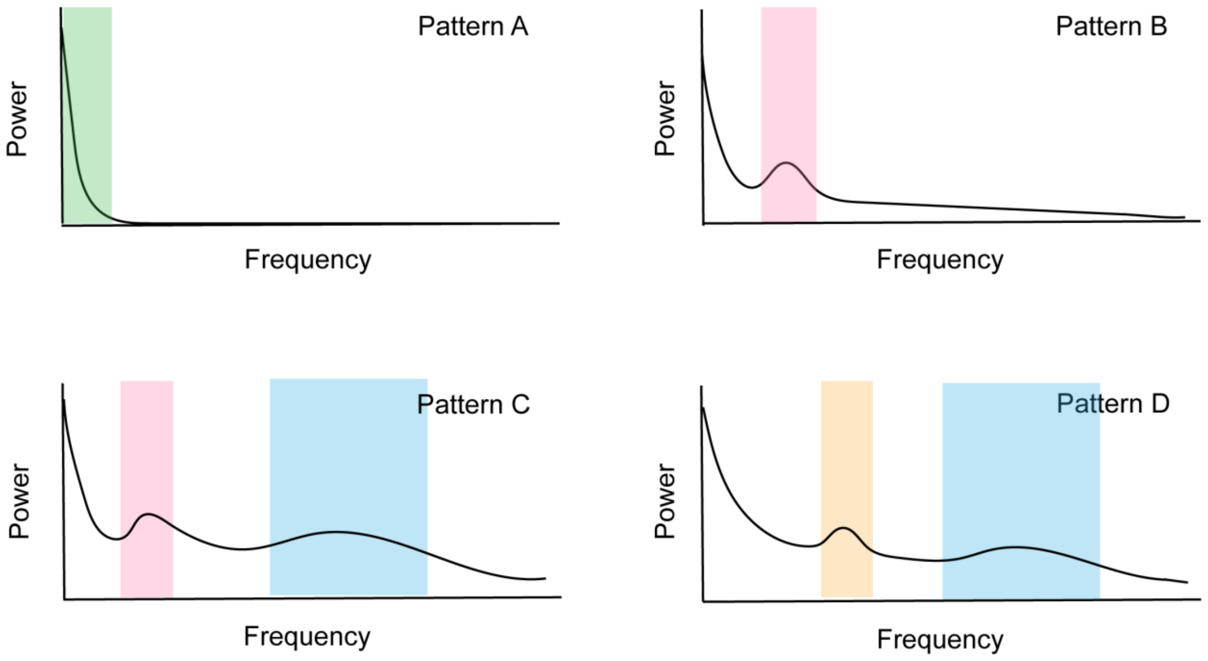

Figure S1. The "ABCD" EEG patterns reflecting the thalamocortical circuit integrity.
A schematic diagram illustrating "ABCD" EEG patterns that reflect the thalamocortical circuit integrity. Green color represents delta frequency; pink color represents theta frequency; orange color represents alpha frequency; blue color represents beta frequency.

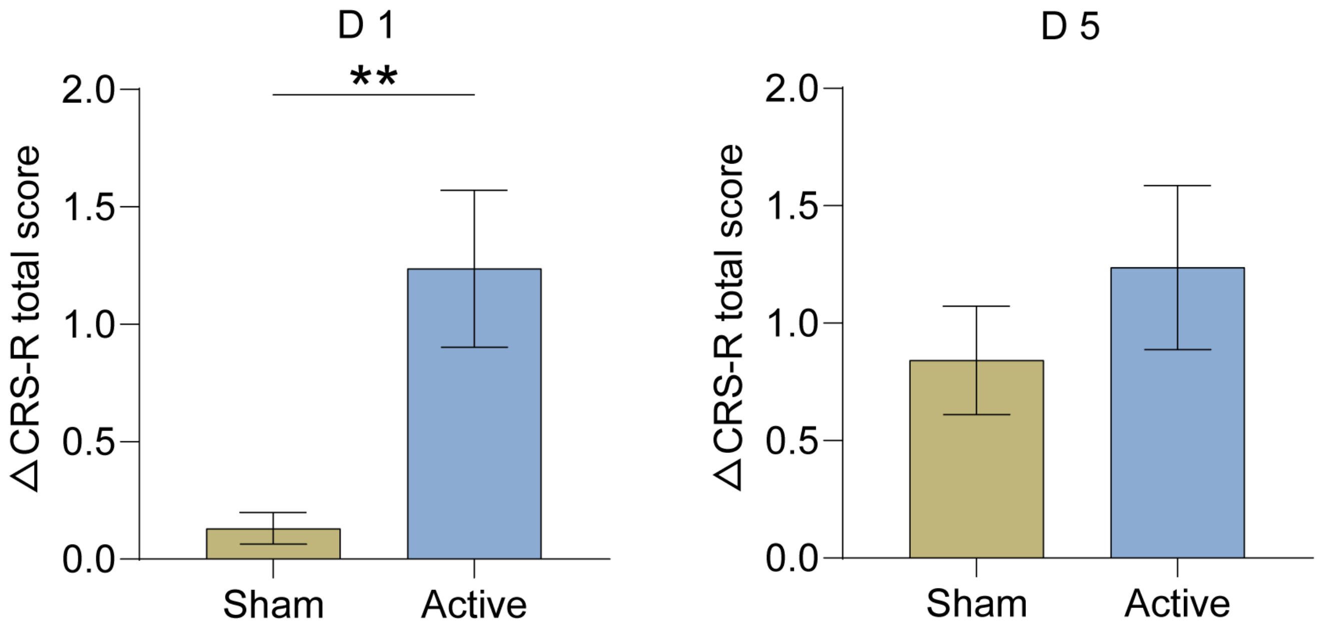

Figure S2. The changes of CRS-R total scores in the per-protocol population.
The between-group differences in the changes of CRS-R total scores after the first treatment session (D1) and after five treatment sessions (D5) in the per-protocol population. ***P* < 0.01, comparison between the active and sham stimulation groups.

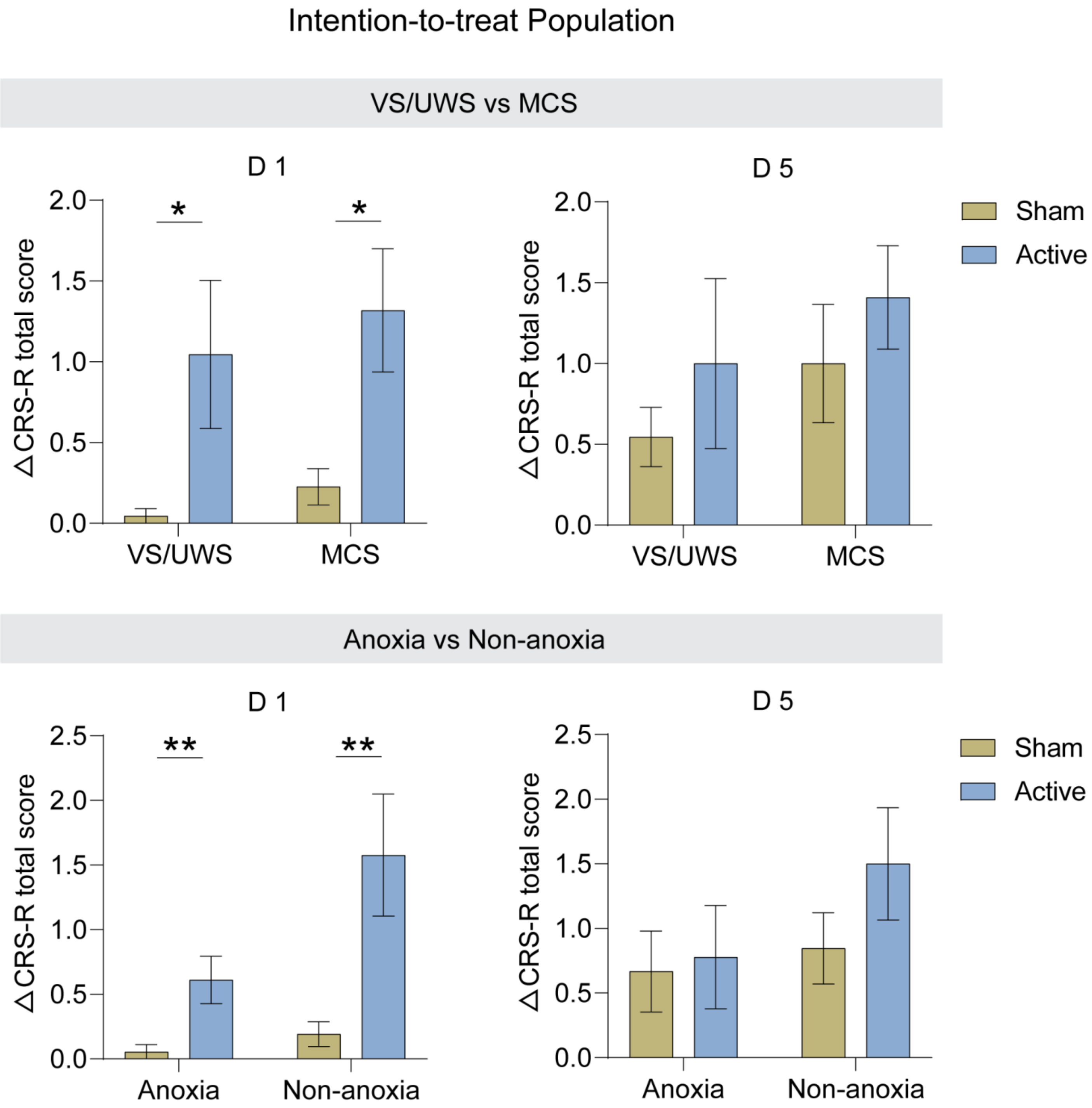

Figure S3. Subgroup analysis of the change of CRS-R total scores in the intention-to-treat population. The between-group differences in the change of CRS-R total scores after the first treatment session (D1) and after five treatment sessions (D5) in patients with different consciousness states and etiologies in the intention-to-treat population. **P* < 0.05, ***P* < 0.01, comparisons between the active and sham stimulation groups. VS/UWS, vegetative state/unresponsive wakefulness syndrome; MCS, minimally conscious state.

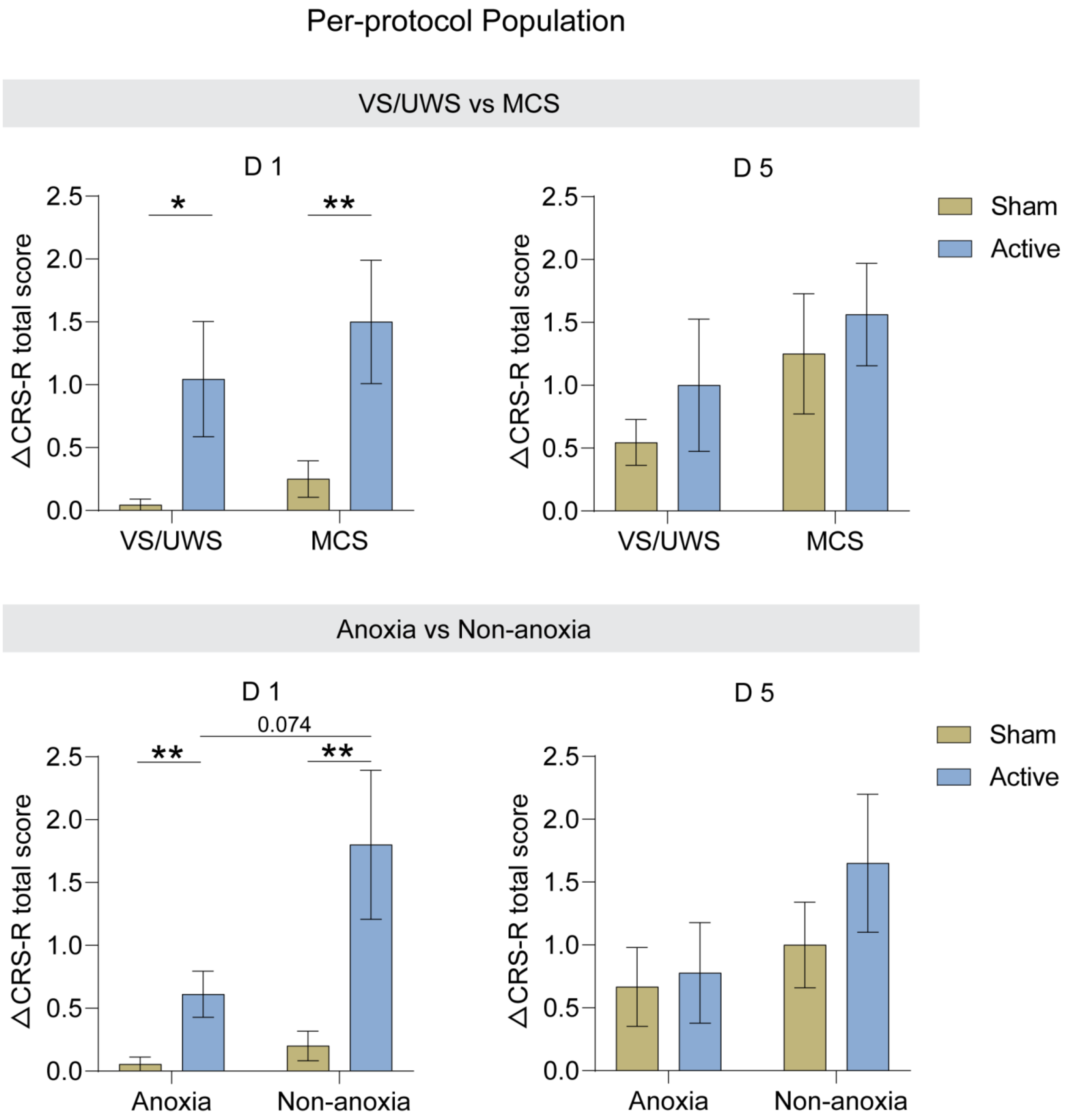

Figure S4. Subgroup analysis of the change of CRS-R total scores in the per-protocol population. The between-group differences in the change of CRS-R total scores after the first treatment session (D1) and after five treatment sessions (D5) in patients with different consciousness states and etiologies in the per-protocol population. *$P < 0.05$, **$P < 0.01$, comparisons between the active and sham stimulation groups. VS/UWS, vegetative state/unresponsive wakefulness syndrome; MCS, minimally conscious state.

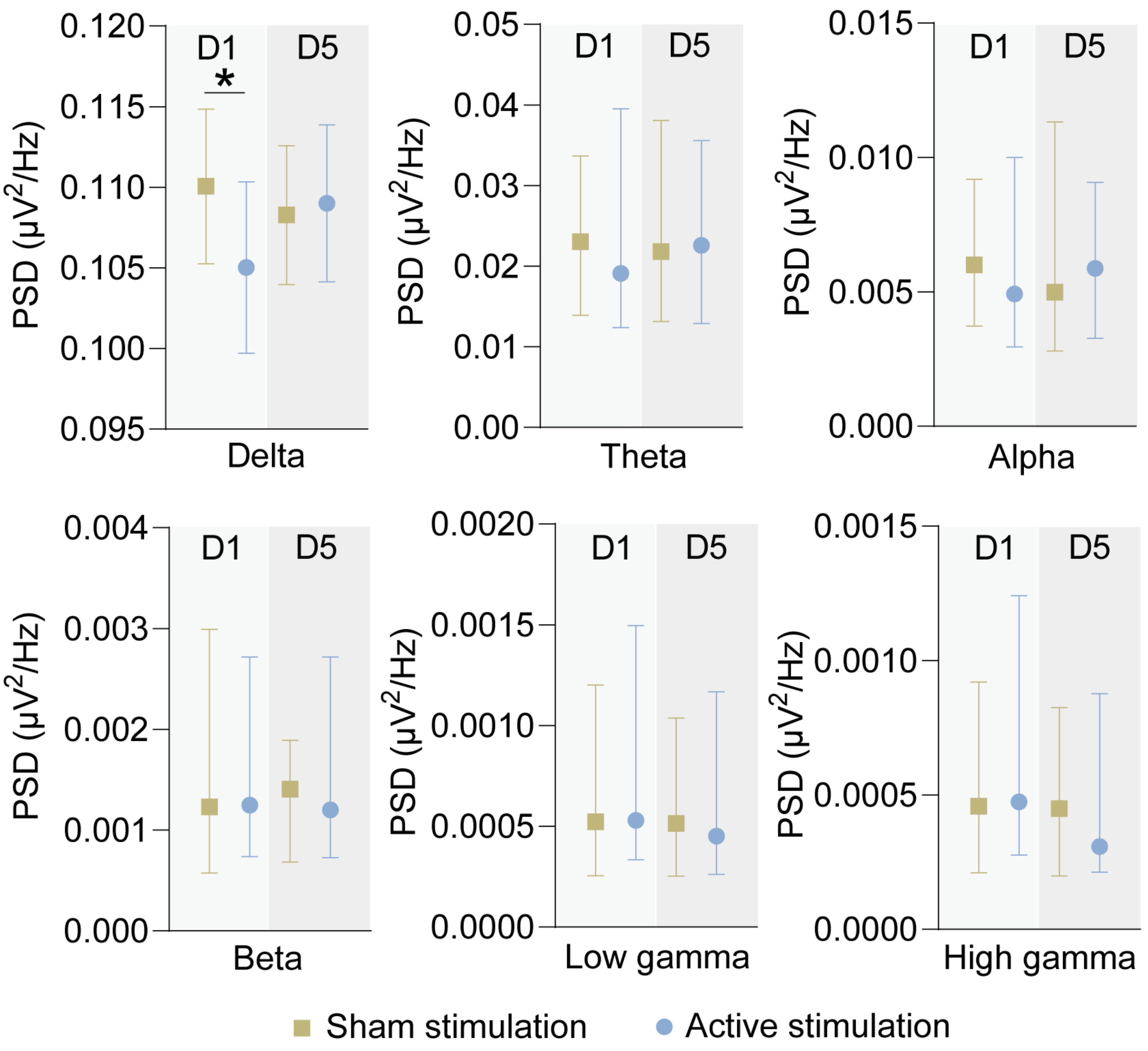

Figure S5. The global power spectral density in patients with DoC.

The between-group differences in the global power spectral density (PSD) after the first treatment session (D1) and after five treatment sessions (D5) in delta, theta, alpha, beta, low gamma, and high gamma frequency bands. Data in delta frequency bands are shown in mean and standard error of mean. Data in theta, alpha, beta, low gamma, and high gamma frequency bands are shown in median and interquartile range. *P<0.05, comparison between the active and sham stimulation groups.

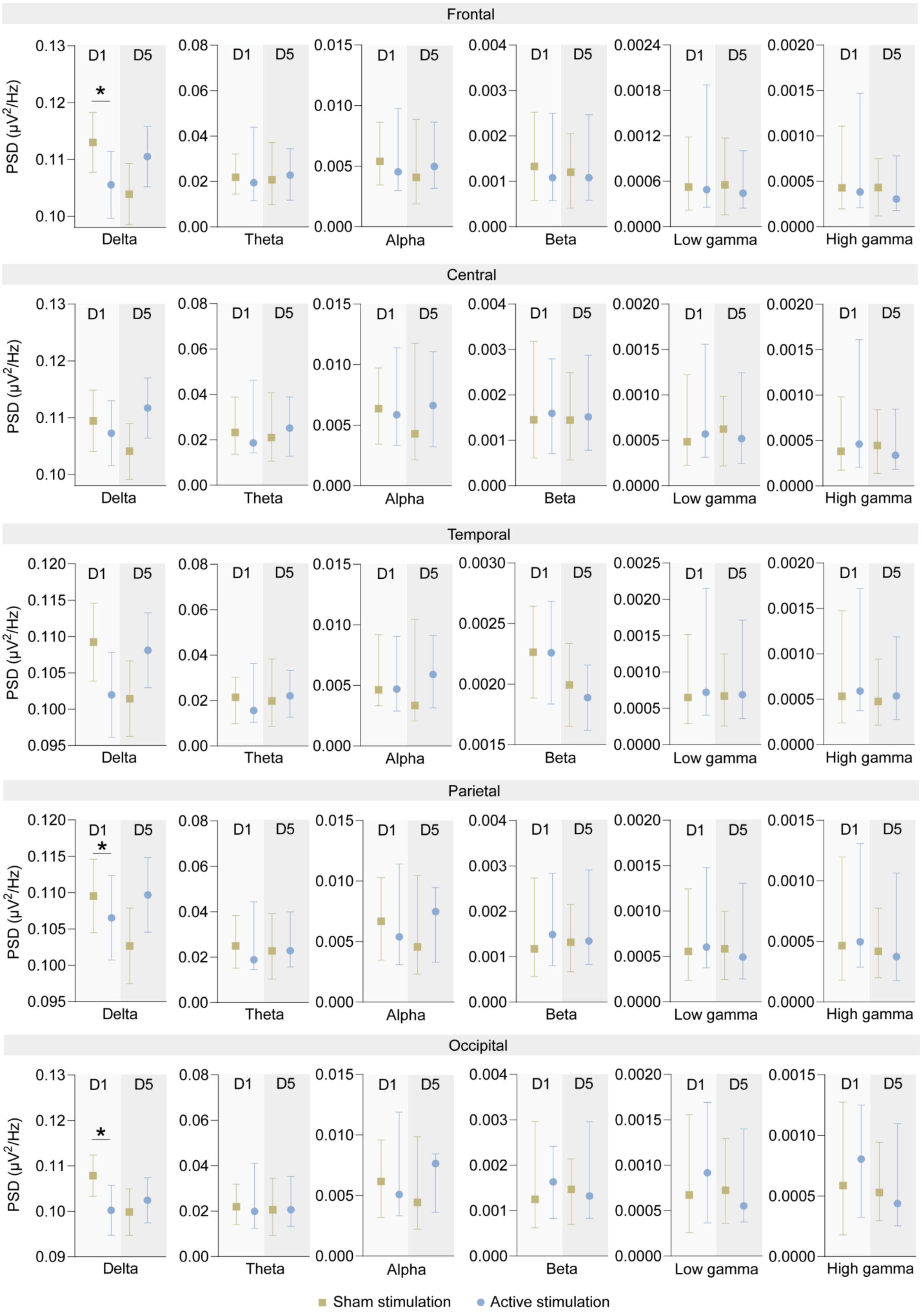
Frontal
Central
Temporal
Parietal
Occipital
PSD (μV2/Hz)
D1
D5
*
Delta
Theta
Alpha
Beta
Low gamma
High gamma
Sham stimulation
Active stimulation

Figure S6. The topographic power spectral density in patients with DoC.

The between-group differences in topographic power spectral density (PSD) of the frontal, central, temporal, parietal, and occipital regions at baseline, after the first treatment session (D1), and after five treatment sessions (D5) in different frequency bands. Data in delta frequency bands are shown in mean and standard error of mean. Data in theta, alpha, beta, low gamma, and high gamma frequency bands are shown in median and interquartile range. *$P<0.05$, comparisons between the active and sham stimulation groups, adjusted using the false discovery rate method.

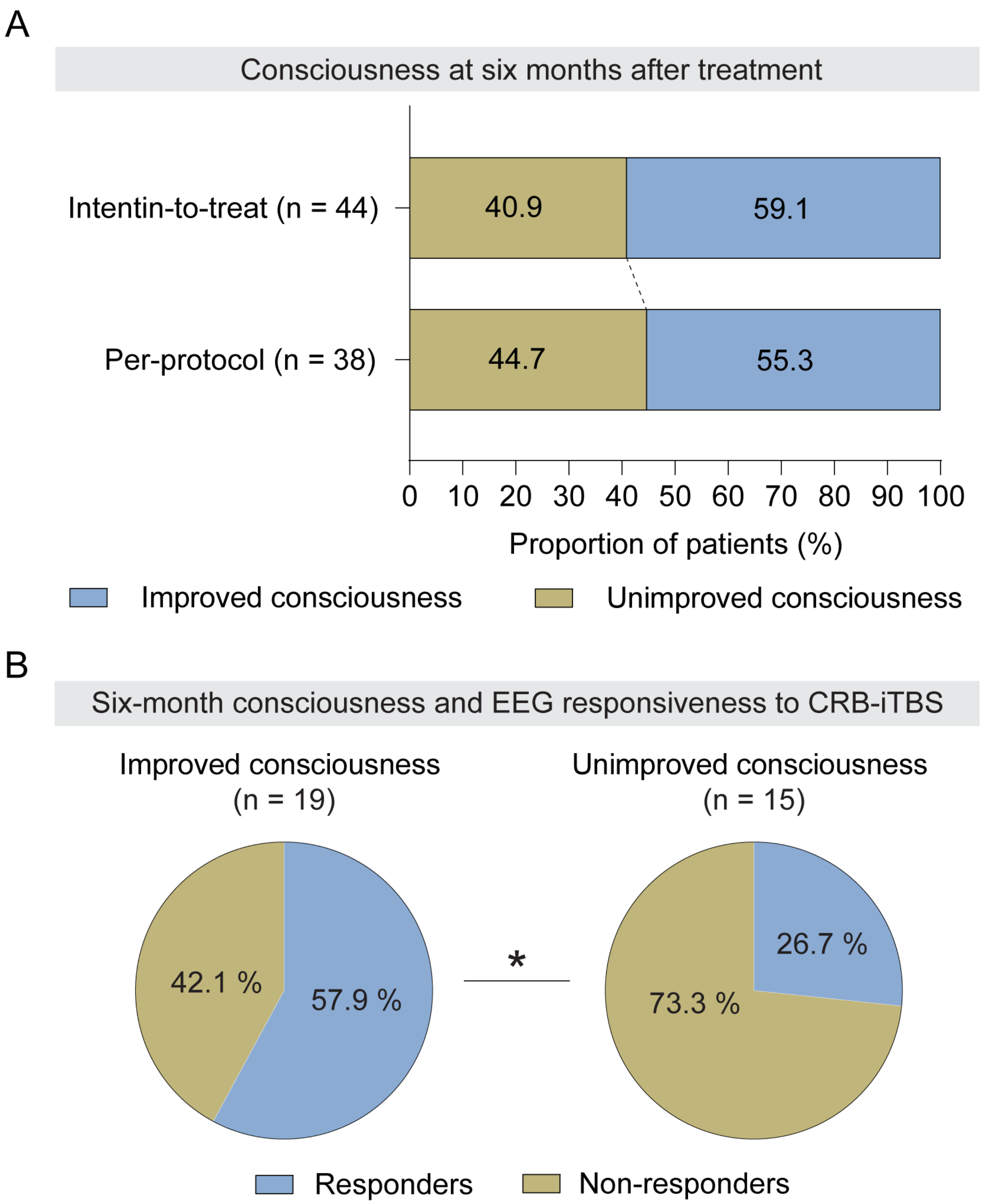

Figure S7. Long-term consciousness recovery of patients receiving CRB-iTBS.
(A) The recovery of consciousness at six months after treatment according the changes in levels of consciousness assessed with CRS-R in the intention-to-treat population and the per-protocol population. **(B)** Six-month consciousness and EEG responsiveness to cerebellar intermittent theta-burst stimulation (CRB-iTBS). Responders were defined as patients who had elevated level of "ABCD" EEG patterns during active stimulation either after the first or the fifth treatment sessions. *$P < 0.01$, comparison between patients with improved consciousness and patients with unimproved consciousness.

Supplementary Tables

Table S1. Detailed clinical and demographic data of the enrolled patients.

ID	Group	Sex, Age (years)	Etiology	Time since injury (days)	Consciousness	Baseline CRS-R (A/V/M/O/C/A)	Period 1		Period 2			Three-month consciousness	Three-month GOSE	Six-month consciousness	Six-month GOSE
							D1 post-stimulation CRS-R (A/V/M/O/C/A)	D5 post-stimulation CRS-R (A/V/M/O/C/A)	D11 pre-stimulation CRS-R (A/V/M/O/C/A)	D11 post-stimulation CRS-R (A/V/M/O/C/A)	D15 post-stimulation CRS-R (A/V/M/O/C/A)				
**1**	Active stimulation-first	Female , 75	Hemorrhagic stroke	88	MCS-	8 (1/1/3/2/0/1)	9(1/1/3/2/0/2)	8(1/1/3/2/0/1)	9(1/1/3/2/0/2)	9(1/1/3/2/0/2)	9(1/1/3/2/0/2)	MCS-	3	MCS-	3
**2**	Sham stimulation-first	Female, 48	Anoxia	17	VS/UWS	6(1/0/2/1/0/2)	6(1/0/2/1/0/2)	6(1/0/2/1/0/2)	6(1/0/2/1/0/2)	7(1/1/2/1/0/2)	7(1/1/2/1/0/2)	MCS-	3	MCS-	3
**3**	Active stimulation-first	Male, 59	Hemorrhagic stroke	123	MCS-	13(2/3/5/1/0/2)	16(2/4/5/2/1/2)	16(2/4/5/2/1/2)	13(2/3/5/1/0/2)	13(2/3/5/1/0/2)	13(2/3/5/1/0/2)	EMCS	4	EMCS	4
**4**	Active stimulation-first	Female, 70	Anoxia	44	VS/UWS	6(1/0/2/1/0/2)	6(1/0/2/1/0/2)	5(1/0/2/1/0/1)	5(1/0/2/1/0/1)	5(1/0/2/1/0/1)	5(1/0/2/1/0/1)	VS/UWS	1	VS/UWS	1

**5**	Sham stimulation-first	Male, 31	Hemorrhagic stroke	127	MCS-	6(0/3/0/1/0/2)	6(0/3/0/1/0/2)	8(1/3/1/2/0/1)	8(1/3/1/2/0/1)	10(2/3/1/2/0/2)	9(1/3/1/2/0/2)	MCS-	3	MCS-	3
**6**	Sham stimulation-first	Male, 62	Hemorrhagic stroke	298	VS/UWS	6(1/0/2/1/0/2)	6(1/0/2/1/0/2)	6(1/0/2/1/0/2)	7(1/0/2/2/0/2)	7(1/0/2/2/0/2)	7(1/0/2/2/0/2)	VS/UWS	2	VS/UWS	2
**7**	Active stimulation-first	Female, 53	Hemorrhagic stroke	86	MCS-	7(1/0/5/0/0/1)	8(1/1/5/0/0/1)	8(1/1/5/0/0/1)	7(1/0/5/0/0/1)	7(1/0/5/0/0/1)	7(1/0/5/0/0/1)	MCS+	3	EMCS	3
**8**	Active stimulation-first	Female, 40	Anoxia	53	VS/UWS	3(1/0/1/0/0/1)	4(1/1/1/0/0/1)	7(1/3/1/0/0/2)	3(1/0/1/0/0/1)	3(1/0/1/0/0/1)	3(1/0/1/0/0/1)	MCS-	3	MCS-	3
**9**	Sham stimulation-first	Female, 46	Hemorrhagic stroke	96	MCS-	9(1/3/1/2/0/2)	9(1/3/1/2/0/2)	9(1/3/1/2/0/2)	9(1/3/1/2/0/2)	10(2/3/1/2/0/2)	9(1/3/1/2/0/2)	MCS+	3	MCS+	3
**10**	Sham stimulation-first	Male, 67	Ischemic stroke	47	MCS-	9(1/0/5/2/0/1)	9(1/0/5/2/0/1)	9(1/0/5/2/0/1)	10(1/1/5/2/0/1)	13(3/1/5/2/1/1)	13(3/1/5/2/1/1)	EMCS	4	EMCS	5
**11**	Sham stimulation-first	Male, 62	Traumatic brain injury	62	MCS-	9(1/3/2/1/0/2)	9(1/3/2/1/0/2)	13(1/3/5/2/0/2)	12(1/3/5/1/0/2)	13(1/3/5/2/0/2)	14(2/3/5/2/0/2)	EMCS	4	EMCS	6
**12**	Active stimulation-first	Female, 64	Osmotic myelinolysis syndrome	16	VS/UWS	6(1/0/2/2/0/1)	6(1/0/2/2/0/1)	5(0/0/2/2/0/1)	5(0/0/2/2/0/1)	5(0/0/2/2/0/1)	5(0/0/2/2/0/1)	VS/UWS	2	VS/UWS	2
**13**	Active stimulation-first	Male, 21	Anoxia	60	VS/UWS	5(1/0/0/2/0/2)	5(1/0/0/2/0/2)	6(1/1/0/2/0/2)	6(1/1/0/2/0/2)	6(1/1/0/2/0/2)	6(1/1/0/2/0/2)	MCS-	3	EMCS	3

**14**	Sham stimulation-first	Male, 34	Anoxia	208	VS/UWS	8(1/1/2/2/0/2)	8(1/1/2/2/0/2)	8(1/1/2/2/0/2)	7(1/1/2/2/0/1)	8(1/1/2/2/0/2)	6(1/1/2/0/0/2)	MCS-	3	MCS-	3
**15**	Sham stimulation-first	Male, 46	Anoxia	65	MCS-	9(2/3/1/1/0/2)	9(2/3/1/1/0/2)	10(2/3/2/1/0/2)	10(2/3/2/1/0/2)	11(2/3/2/2/0/2)	11(2/3/2/2/0/2)	EMCS	3	EMCS	3
**16**	Active stimulation-first	Female, 60	Osmotic myelinolysis syndrome	45	MCS-	7(1/2/0/2/0/2)	8(1/3/0/2/0/2)	10(1/3/2/2/0/2)	9(1/3/1/2/0/2)	10(1/3/2/2/0/2)	13(3/3/2/2/1/2)	MCS-	1	MCS-	1
**17**	Active stimulation-first	Female, 82	Anoxia	81	VS/UWS	1(0/0/0/0/0/1)	3(0/0/2/1/0/0)	4(0/0/2/1/0/1)	4(0/0/2/1/0/1)	4(0/0/2/1/0/1)	3(0/0/2/0/0/1)	VS/UWS	1	VS/UWS	1
**18**	Active stimulation-first	Female, 60	Hemorrhagic stroke	74	MCS-	11(2/3/2/1/1/2)	19(4/4/5/2/1/3)	14(2/3/5/2/0/2)	14(2/3/5/2/0/2)	16(3/3/5/2/1/2)	18(3/4/5/2/1/3)	MCS+	3	EMCS	3
**19**	Active stimulation-first	Female, 47	Anoxia	145	VS/UWS	6(2/0/0/2/0/2)	8(2/0/2/2/0/2)	7(1/0/2/2/0/2)	7(1/0/2/2/0/2)	7(1/0/2/2/0/2)	8(2/0/2/2/0/2)	MCS-	3	MCS-	3
**20**	Sham stimulation-first	Male, 49	Traumatic brain injury	25	VS/UWS	4(1/0/1/1/0/1)	4(1/0/1/1/0/1)	5(1/1/1/1/0/1)	9(0/1/5/2/0/1)	11(0/3/5/2/0/1)	16(3/3/5/2/1/2)	EMCS	3	EMCS	4
**21**	Sham stimulation-first	Male, 48	Anoxia	35	VS/UWS	5(1/0/2/1/0/1)	5(1/0/1/1/0/2)	6(1/0/2/1/0/2)	5(1/0/2/1/0/1)	5(1/0/2/1/0/1)	6(1/0/2/1/0/2)	VS/UWS	2	VS/UWS	2
**22**	Active stimulation-first	Male, 78	Hemorrhagic stroke	87	MCS+	17(4/3/5/1/1/3)	18(4/3/5/2/1/3)	16(3/3/5/2/1/2)	16(3/3/5/2/1/2)	16(3/3/5/2/1/2)	16(3/3/5/2/1/2)	MCS+	3	EMCS	3

**23**	Active stimulation-first	Male, 56	Anoxia	40	VS/UWS	8(2/1/2/1/0/2)	8((2/1/2/1/0/2)	6(1/0/2/1/0/2)	5(0/0/2/1/0/2)	5(0/0/2/1/0/2)	6(1/0/2/1/0/2)	VS/UWS	2	VS/UWS	1
**24**	Sham stimulation-first	Female, 76	Ischemic stroke	18	VS/UWS	6(1/0/2/1/0/2)	6(1/0/2/1/0/2)	8(1/0/3/2/0/2)	13(3/2/3/2/1/2)	16(4/4/3/2/1/2)	13(3/2/3/2/1/2)	MCS+	3	MCS+	1
**25**	Active stimulation-first	Male, 53	Traumatic brain injury	20	VS/UWS	2(0/0/1/0/0/1)	2(0/0/1/0/0/1)	3(0/0/2/0/0/1)	2(0/0/1/0/0/1)	2(0/0/1/0/0/1)	3(1/0/1/0/0/1)	EMCS	3	EMCS	4
**26**	Active stimulation-first	Male, 38	Anoxia	29	VS/UWS	5(1/0/2/0/0/2)	5(1/0/2/0/0/2)	3(0/0/2/0/0/1)	5(1/0/2/0/0/2)	5(1/0/2/0/0/2)	4(0/0/2/1/0/1)	VS/UWS	2	VS/UWS	2
**27**	Sham stimulation-first	Male, 79	Ischemic stroke	110	MCS-	6(0/0/5/1/0/0)	6(0/0/5/1/0/0)	7(1/0/5/1/0/0/)	6(0/0/5/1/0/0)	6(0/0/5/1/0/0)	6(0/0/5/1/0/0)	MCS-	3	MCS-	1
**28**	Sham stimulation-first	Male, 70	Hemorrhagic stroke	33	MCS-	10(2/2/3/1/0/2)	11(2/2/3/2/0/2)	9(2/2/3/1/0/1)	10(2/2/3/1/0/2)	11(2/2/3/2/0/2)	10(2/3/2/1/0/2)	MCS-	1	MCS-	1
**29**	Active stimulation-first	Male, 60	Traumatic brain injury	59	MCS+	17(3/3/5/2/1/3)	17(3/3/5/2/1/3)	22(4/4/6/3/2/3)	23(4/5/6/3/2/3)	23(4/5/6/3/2/3)	23(4/5/6/3/2/3)	EMCS	4	EMCS	4
**30**	Sham stimulation-first	Female, 67	Anoxia	18	VS/UWS	5(1/0/2/1/0/1)	5(1/0/2/1/0/1)	6(1/1/2/1/0/1)	4(0/0/2/1/0/1)	6(1/0/2/1/0/2)	5(1/0/2/0/0/2)	MCS-	3	MCS-	3
**31**	Active stimulation-first	Female, 65	Anoxia	20	VS/UWS	3(0/0/1/1/0/1)	4(0/0/1/1/0/2)	5(0/0/2/1/0/2)	4(0/0/1/1/0/2)	4(0/0/1/1/0/2)	4(0/0/2/0/0/2)	VS/UWS	2	VS/UWS	2

**32**	Active stimulation-first	Male, 31	Anoxia	21	MCS-	9(1/3/2/1/0/2)	9(1/3/2/1/0/2)	12(2/3/3/2/0/2)	12(2/3/3/2/0/2)	12(2/3/3/2/0/2)	17(4/3/5/2/1/2)	EMCS	1	EMCS	1
**33**	Sham stimulation-first	Female, 31	Anoxia	64	VS/UWS	3(1/0/0/0/0/2)	3(1/0/0/0/0/2)	5(1/1/1/0/0/2)	6(1/1/1/1/0/2)	6(1/1/1/1/0/2)	8(1/2/2/1/0/2)	VS/UWS	2	VS/UWS	1
**34**	Sham stimulation-first	Male, 52	Traumatic brain injury	39	VS/UWS	5(0/0/2/1/0/2)	5(0/0/2/1/0/2)	7(1/1/2/1/0/2)	5(0/0/2/1/0/2)	5(0/0/2/1/0/2)	5(0/0/2/1/0/2)	VS/UWS	2	VS/UWS	2
**35**	Active stimulation-first	Male, 71	Anoxia	46	VS/UWS	5(1/0/1/1/0/2)	5(1/0/1/1/0/2)	4(1/0/0/1/0/2)	4(1/0/0/1/0/2)	4(0/0/2/1/0/1)	4(0/0/2/1/0/1)	VS/UWS	2	VS/UWS	2
**36**	Sham stimulation-first	Male, 22	Anoxia	252	MCS+	13(3/3/2/2/1/2)	13(3/3/2/2/1/2)	13(3/3/2/2/1/2)	13(3/3/2/2/1/2)	13(3/3/2/2/1/2)	14(3/3/3/2/1/2)	MCS+	3	MCS+	3
**37**	Sham stimulation-first	Male, 28	Anoxia	23	VS/UWS	3(1/0/1/0/0/1)	3(1/0/1/0/0/1)	4(1/0/2/0/0/1)	3(1/0/0/0/0/2)	3(1/0/0/0/0/2)	3(1/0/0/0/0/2)	VS/UWS	2	MCS-	3
**38**	Active stimulation-first	Female, 45	Hemorrhagic stroke	49	VS/UWS	4(0/0/2/1/0/1)	5(0/0/2/1/0/2)	3(0/0/2/0/0/1)	4(1/0/2/0/0/1)	4(1/0/2/0/0/1)	4(0/0/2/1/0/1)	MCS-	3	MCS-	3
**39**	Sham stimulation-first	Male, 50	Hemorrhagic stroke	42	MCS+	15(3/3/4/2/1/2)	15(3/3/4/2/1/2)	15(3/3/4/2/1/2)	15(3/3/4/2/1/2)	15(3/3/4/2/1/2)	15(3/3/4/2/1/2)	EMCS	3	EMCS	3
**40**	Active stimulation-first	Male, 41	Osmotic myelinolysis syndrome	45	MCS-	13(1/3/5/2/0/2)	16(2/3/5/2/1/3)	15(2/3/5/2/1/2)	15(2/3/5/2/1/2)	15(2/3/5/2/1/2)	15(2/3/5/2/1/2)	EMCS	1	EMCS	1

**41**	Active stimulation-first	Female, 48	Metabolic encephalopathy	50	MCS+	16(2/4/5/2/1/2)	17(3/4/5/2/1/2)	18(3/4/5/2/1/3)	18(3/4/5/2/1/3)	18(3/4/5/2/1/3)	18(3/4/5/2/1/3)	MCS+	3	EMCS	4
**42**	Sham stimulation-first	Female, 60	Traumatic brain injury	21	MCS-	11(1/3/5/1/0/1)	11(1/3/4/2/0/1)	13(1/3/5/2/0/2)	13(1/3/5/2/0/2)	13(1/3/5/2/0/2)	13(1/3/5/2/0/2)	MCS-	1	MCS-	1
**43**	Active stimulation-first	Female, 56	Hemorrhagic stroke	28	MCS+	17(3/4/5/2/1/2)	18(3/4/5/2/1/3)	19(3/5/5/2/1/3)	19(3/5/5/2/1/3)	19(3/5/5/2/1/3)	19(3/5/5/2/1/3)	EMCS	3	EMCS	4
**44**	Sham stimulation-first	Female, 29	Traumatic brain injury	55	MCS+	14(3/3/4/1/1/2)	14(3/3/4/1/1/2)	14(3/3/4/1/1/2)	14(3/3/4/1/1/2)	14(3/3/4/1/1/2)	14(3/3/4/1/1/2)	MCS+	3	EMCS	3

VS/UWS, vegetative state/unresponsive wakefulness syndrome; MCS-, minimal conscious state minus; MCS+, minimal conscious state plus; CRS-R, Coma Recovery Scale-Revised;
A/V/M/O/C/A, auditory/visual/motor/oromotor/communication/arousal subscales of the CRS-R; NA, not available;
D1, day 1; D5, day 5; D11, day 11; D15, day 15.
Patients #1-38 were included in the per-protocol population. Patients #39-44 did not follow protocol and were excluded from the per-protocol population.

Table S2. Baseline demographic and clinical characteristics of the per-protocol population.

	Total (n = 38)	Active stimulation-first group (n = 20)	Sham stimulation-first group (n = 18)	*P* value
**Age, years**	54.5 (43.3 - 66.5)	59.5 (46.5 - 66.3)	48.5 (37.0 - 65.8)	0.380
**Sex**				0.112
Female	16 (42.1 %)	11 (55.0 %)	5 (27.8 %)	
Male	22 (57.9 %)	9 (45.0 %)	13 (72.2 %)	
**Time since brain injury**				
≤28 days	9 (23.7 %)	4 (20.0 %)	5 (27.8 %)	0.709
> 28 days	29 (76.3 %)	16 (80.0 %)	13 (72.2 %)	
**Consciousness**				1.000
VS/UWS	22 (57.9 %)	12 (60.0 %)	10 (55.6 %)	
MCS	16 (42.1 %)	8 (40.0 %)	8 (44.4 %)	
**CRS-R score**				
Total	6.0 (5.0 - 9.0)	6.0 (4.8 - 8.3)	6.0 (5.0 - 9.0)	0.848
Auditory	1.00 (1.00 - 1.00)	1.00 (1.00 - 2.00)	1.00 (1.00 - 1.00)	0.623
Visual	0.00 (0.00 - 2.75)	0.00 (0.00 - 2.25)	0.00 (0.00 - 2.75)	0.987
Motor	2.00 (1.00 - 2.00)	2.00 (1.00 - 2.25)	2.00 (1.00 - 2.00)	0.939
Oromotor/verbal	1.00 (1.00 - 1.75)	1.00 (0.75 - 2.00)	1.00 (1.00 - 1.00)	0.833
Communication	0.00 (0.00 - 0.00)	0.00 (0.00 - 0.00)	0.00 (0.00 - 0.00)	0.364
Arousal	2.00 (1.00 - 2.00)	2.00 (1.00 - 2.00)	2.00 (1.00 - 2.00)	0.907
**Etiology**				0.659
Anoxia	18 (47.4 %)	10 (50.0 %)	8 (44.4 %)	
Stroke				
Hemorrhagic stroke	10 (26.3 %)	6 (30.0 %)	4 (22.2 %)	
Ischemic stroke	3 (7.9 %)	0 (0)	3 (16.7 %)	
TBI	5 (13.2 %)	2 (10.0 %)	3 (16.7 %)	
Other*	2 (5.3 %)	2 (10.0 %)	0 (0)	

Data are presented as n (%), mean (standard deviation), or median (interquartile range). VS/UWS, vegetative state/unresponsive wakefulness syndrome; MCS, minimally conscious state; CRS-R, Coma Recovery Scale-Revised; TBI, traumatic brain injury. *Other etiology refers to osmotic demyelination syndrome.

Table S3. Changes in Coma Recovery Scale-Revised subscale scores in the intention-to-treat population.

	Active stimulation (n=44)	Sham stimulation (n=44)	Active stimulation vs Sham stimulation Mean difference (95% CI)	*P* value
**Changes in CRS-R subscale scores after five treatment sessions**				
Auditory	0.19 ± 0.79	0.18 ± 0.62	0.014 (-0.287 – 0.315)	0.926
Visual	0.35 ± 0.72	0.14 ± 0.35	0.217 (-0.007 – 0.441)	0.057
Motor	0.33 ± 0.78	0.32 ± 0.67	0.006 (-0.302 – 0.314)	0.967
Oromotor/verbal	0.12 ± 0.59	0.09 ± 0.42	0.030 (-0.186 – 0.245)	0.784
Communication	0.12 ± 0.39	0.07 ± 0.25	0.050 (-0.093 – 0.192)	0.490
Arousal	0.14 ± 0.52	-0.02 ± 0.40	0.166 (-0.019 – 0.351)	0.078
**Changes in CRS-R subscale scores after the first treatment session**				
Auditory	0.27 ± 0.66	0.00 ± 0.22	0.263 (0.054 – 0.472)	**0.014**
Visual	0.27 ± 0.73	0.02 ± 0.15	0.289 (0.030 – 0.549)	**0.030**
Motor	0.18 ± 0.62	0.02 ± 0.40	0.159 (-0.059 – 0.377)	0.150
Oromotor/verbal	0.18 ± 0.39	0.05 ± 0.21	0.136 (0.014 – 0.259)	**0.030**
Communication	0.09 ± 0.29	0.02 ± 0.15	0.068 (-0.029 – 0.166)	0.168
Arousal	0.18 ± 0.45	0.00 ± 0.22	0.182 (0.033 – 0.330)	**0.017**

CRS-R, Coma Recovery Scale-Revised; CI, confidence interval.

Table S4. Changes in Coma Recovery Scale-Revised subscale scores in the per-protocol population.

	Active stimulation (n=38)	Sham stimulation (n=38)	Active stimulation vs Sham stimulation	
			**Mean difference (95% CI)**	***P* value**
**Changes in CRS-R subscale scores after five treatment sessions**				
Auditory	0.16 ± 0.82	0.21 ± 0.66	-0.053 (-0.386 – 0.281)	0.754
Visual	0.37 ± 0.75	0.16 ± 0.37	0.211 (-0.041 – 0.462)	0.099
Motor	0.37 ± 0.82	0.37 ± 0.71	0.000 (-0.353 – 0.353)	1.000
Oromotor/verbal	0.13 ± 0.62	0.08 ± 0.43	0.053 (-0.190 – 0.295)	0.666
Communication	0.11 ± 0.39	0.08 ± 0.27	0.026 (-0.130 – 0.182)	0.737
Arousal	0.11 ± 0.51	-0.05 ± 0.40	0.158 (-0.034 – 0.350)	0.106
**Changes in CRS-R subscale scores after the first treatment session**				
Auditory	0.26 ± 0.69	0.00 ± 0.23	0.263 (0.054 - 0.472)	**0.014**
Visual	0.32 ± 0.77	0.03 ± 0.16	0.250 (0.030 - 0.549)	**0.030**
Motor	0.21 ± 0.66	0.05 ± 0.40	0.158 (-0.091 - 0.407)	0.210
Oromotor/verbal	0.21 ± 0.41	0.03 ± 0.16	0.184 (0.057 – 0.311)	**0.005**
Communication	0.08 ± 0.27	0.03 ± 0.16	0.053 (-0.048 – 0.153)	0.300
Arousal	0.16 ± 0.44	0.00 ± 0.23	0.158 (-0.002 – 0.318)	0.053

CRS-R, Coma Recovery Scale-Revised; CI, confidence interval.

Table S5. Baseline "ABCD" EEG patterns of patients with favorable and unfavorable functional outcomes

	Pattern A (n = 5)	Pattern B (n = 22)	Pattern C (n = 7)	Pattern D (n = 0)	*P* value
**Functional outcomes**					0.223
Favorable, GOSE ⩾ 4 (n = 6)	0 (0)	4 (18.2 %)	2 (28.6 %)	0 (0)	
Unfavorable, GOSE < 4 (n = 28)	5 (100.0 %)	18 (81.8 %)	5 (71.4 %)	0 (0)	

Data are presented as n (%). GOSE, Glasgow Outcome Scale-Extended.

Table S6. Baseline "ABCD" EEG patterns of patients with improved and unimproved consciousness

	Pattern A (n = 5)	Pattern B (n = 22)	Pattern C (n = 7)	Pattern D (n = 0)	*P* value
**Consciousness**					**0.014**
Improved consciousness (n = 19)	1 (20.0 %)	11 (50.0 %)	7 (100.0 %)	0 (0)	
Unimproved consciousness (n = 15)	4 (80.0 %)	11 (50.0 %)	0 (0)	0 (0)	

Data are presented as n (%).
