# Supplementary material for: Effect of cerebellar stimulation on awareness recovery in disorders of consciousness (CARE-DoC): A randomized, sham-controlled, crossover clinical trial
Source: Neurotherapeutics. 2025 Jul 5;22(5):e00635. doi: 10.1016/j.neurot.2025.e00635 (PMC12491794; doi:10.1016/j.neurot.2025.e00635)
Supplement: Multimedia component 2 [file mmc2.pdf]

# Clinical study Protocol

Effect of Cerebellar Stimulation on the Awareness Recovery in Disorders of Consciousness: A randomized, controlled, double-blind, cross-over trial

This supplementary file contains the following items:


1. Protocol version 1.0 (original)  page 2-27
2. Protocol version 2.0 (final)  page 28-55
3. Summary of protocol amendments  page 56-61
4. Original statistical analysis plan  page 62-69
5. Final statistical analysis plan  page 70-77
6. Summary of statistical analysis plan amendments  page 78-81

ClinicalTrials registration: NCT05558930

Effect of Cerebellar Stimulation on the Awareness Recovery in Disorders of Consciousness: A randomized, controlled, double-blind, cross-over trial

# CLINICAL STUDY PROTOCOL(Original)

Research site: Xijing Hospital

Principle Investigator: Wen Jiang

Responsible department: Department of Neurology

Contact: +86-29-84771319

Version 1.0-March 2022

## Table of Contents

STUDY SYNOPSIS ..... 5
ABBREVIATION ..... 7
1 STUDY FLOW CHART ..... 8
2 BACKGROUND ..... 9
3 STUDY OBJECTIVES ..... 10
3.1 Primary objective(s) ..... 10
3.2 Secondary objective(s) ..... 10
3.3 Safety outcome(s) ..... 11
4 STUDY DESIGN ..... 11
4.1 General study design ..... 11
5 RANDOMIZATION ..... 11
6 STUDY POPULATION ..... 12
6.1 Eligibility criteria ..... 12
6.2 Recruitment and screening ..... 12
6.3 Assignment to study groups ..... 12
7 STUDY INTERVENTION ..... 12
7.1 Rest motor threshold determination ..... 12
7.2 iTBS stimulation ..... 13
7.3 Sham stimulation ..... 13
7.4 Concomitant treatments ..... 13
8 STUDY ASSESSMENTS ..... 13
8.1 Level of consciousness ..... 13
8.2 Functional outcome assessment ..... 14
8.3 EEG recording and analysis ..... 14
8.3.1 EEG recordings ..... 14
8.3.2 EEG data pre-processing ..... 14
8.3.3 Power spectra density and EEG pattern categorization ..... 14
9 DATA MANAGEMENT AND QUALITY ASSURANCE ..... 15
9.1 Confidentiality ..... 15
9.2 Data handling and source document identification ..... 15
9.3 Record keeping and archiving ..... 15
10 SAFETY ..... 15
10.1 Definitions of safety outcomes ..... 15
10.2 Recording and reporting ..... 16
10.3 Follow up ..... 16
11 END OF THE TRIAL AND WITHDRAWAL RULES ..... 16
11.1 Definition of end of the trial ..... 16
11.2 Rules for withdrawal of participants ..... 16
12 STUDY ADMINISTRATIVE STRUCTURE ..... 17
12.1 Principal Investigator (PI) ..... 17
12.2 Academic Committee (AC) ..... 17
12.3 Data and Safety Monitoring Board (DSMB) ..... 17

12.4 Executive Committee (EC) ........ 17
13 STATISTICAL CONSIDERATIONS ........ 18
13.1 Determination of Sample Size ........ 18
13.2 Planned Analyses ........ 18
13.3 Handling of missing data and drop-outs ........ 19
14 QUALITY ASSURANCE AND CONTROL ........ 19
14.1 Independent oversight organization ........ 19
14.2 Training of the study team ........ 19
14.3 Monitoring of study procedures ........ 20
15 ETHICAL ASPECTS ........ 20
15.1 Study registration ........ 20
15.2 Ethical Conduct of the Study ........ 20
15.3 Patient Information and Informed Consent ........ 20
15.4 Participant privacy and confidentiality ........ 20
15.5 Protocol amendments ........ 20
16 PUBLICATION AND DISSEMINATION POLICY ........ 21
17 FUNDING ........ 21
18 APPENDICES ........ 22
19 REFERENCES ........ 23

# STUDY SYNOPSIS

| | |
|---|---|
| Sponsor / Sponsor-Investigator: | Xijing Hospital, The Forth Military Medical University-Prof. Wen Jiang, MD, PhD<br>*(investigator-initiated trial)* |
| Study Title: | Effect of cerebellar stimulation on the awareness recovery in disorders of consciousness |
| Short Title / Study ID: | CARE-DOC |
| Protocol Version and Date: | V1.0, March 2022 |
| Trial registration: | www.clinicaltrial.gov NCT05558930 |
| Study design: | A randomized, double-blind, sham-controlled, crossover trial |
| Objective(s): | To evaluate the effects of cerebellar intermittent theta burst stimulation (iTBS) in promoting consciousness recovery in patients with disorders of consciousness (DoC) |
| Primary outcome (s): | • The difference in the change of Coma Recovery Scale-Revised (CRS-R) scores between the iTBS and sham stimulation groups after the 5-session treatment |
| Secondary outcome (s): | • The between-group differences in the "ABCD" EEG patterns after the fifth session of stimulation.<br>• The Glasgow Outcome Scale-Extended (GOS-E) scores of all the participants after 3 months and 6 months of enrollment |
| Inclusion / Exclusion criteria: | Inclusion:<br>• Vegetative state (VS) / unresponsive wakefulness syndrome (UWS) or minimally conscious state (MCS) according to at least two CRS-R assessments<br>• Age ≥18 years<br>• Written informed consent obtained from legal surrogates<br>Exclusion:<br>• Patients in coma<br>• Brain injury <1 week<br>• Presence of metallic hardware in close contact with the discharging coil (such as cochlear implants, an Internal Pulse Generator or medication pumps)<br>• Patients with high risks according to standard questionnaire to screen transcranial magnetic stimulation (TMS) candidates |
| Measurements and procedures: | Eligible patients will receive bilateral cerebellar iTBS and sham stimulation once daily for 5 consecutive days, either iTBS stimulation first or sham stimulation first, |

| | |
|---|---|
| | separated by a 5-day washout period. Demographic, MRI and clinical information are collected at enrolment. The level of consciousness is assessed with CRS-R before and after each stimulation session within 2 hours.<br>Additionally, 32-channel EEG will be performed 20 minutes before and after the first and after the fifth treatment sessions within 30 minutes.<br>Analyses will compare the effects of iTBS and sham stimulation on the CRS-R score and EEG and assess the functional outcomes of all the participants 3 months and 6 months after enrolment. |
| Study Intervention: | Bilateral cerebellar iTBS stimulation under neuronavigation |
| Number of Participants | 44 |

# ABBREVIATION

| | |
|---|---|
| AC | Academic Committee |
| AE | adverse event |
| CLMM | cumulative link mixed effects model |
| CRF | case report form |
| CRS-R | Coma Recovery Scale-Revised |
| DSMB | Data and Safety Monitoring Board |
| DoC | disorders of consciousness |
| DLPFC | dorsolateral prefrontal cortex |
| DSMB | Data and Safety Monitoring Board |
| EEG | electroencephalography |
| EMCS | emergence from MCS |
| EC | Ethics Committee |
| EC | Executive Committee |
| FFT | fast fourier transform |
| GLM | generalized linear model |
| GPi | globus pallidus interna |
| HF-rTMS | high-frequency rTMS |
| ICA | independent component analysis |
| ITT | intention-to-treat |
| iTBS | intermittent theta burst stimulation |
| IQR | interquartile range |
| LOCF | last observation carried forward |
| LMM | linear mixed model |
| MRI | magnetic resonance imaging |
| MCS | minimally conscious state |
| PP | per-protocol |
| PPC | posterior parietal cortex |
| PSD | power spectral density |
| PI | principal investigator |
| rTMS | repetitive transcranial magnetic stimulation |
| RMT | resting motor thresholds |
| SAE | serious adverse event |
| SAP | statistical analysis plan |
| SCS | spinal cord stimulation |
| SD | standard deviation |
| GOS-E | Glasgow Outcome Scale-Extended |
| tDCS | transcranial direct current stimulation |
| TMS | transcranial magnetic stimulation |
| TBI | traumatic brain injury |
| UWS | unresponsive wakefulness syndrome |
| VNS | vagus nerve electrical stimulation |
| VS | vegetative state |

# 1 STUDY FLOW CHART

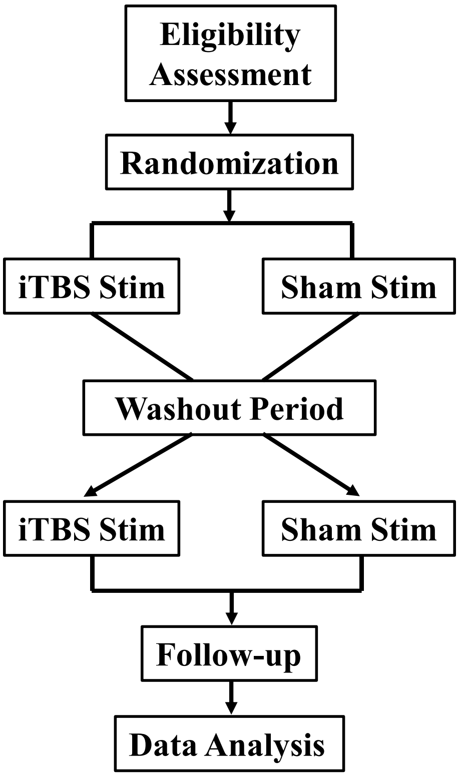


**Flow chart**: iTBS=intermittent transcranial magnetic stimulation; stim=stimulation

# 2 BACKGROUND

Disorders of consciousness (DoC) are characterized by alterations in arousal and/or awareness and are caused by severe brain injuries that disrupt the functional connectivity of corticothalamic system and its connection between basal ganglia and limbic system, to weaken or stop the excitatory synaptic activity in the cerebral cortex. Cardiac arrest, traumatic brain injury (TBI), intracerebral hemorrhage, and ischemic stroke are common causes of DoC.[1] Patients who survive coma after the acute stage of brain injury but still do not awaken often step into a phase of VS/UWS (arousal without awareness)[2] or minimally conscious state (MCS) (minimal, reproducible but inconsistent awareness)[3].

**Lack of evidently effective treatment for recovery of patients with DoC**

Pharmacological treatments that focused on promoting dopamine signaling[4], such as amantadine[5], methylphenidate[6], levodopa[7], bromocriptine[8], and subcutaneous apomorphine[9], have been tested in previous trials to promote recovery in patients with DoC. However, only amantadine has been confirmed to have some effect on restoring consciousness in patients with VS/UWS and MCS 4-16 weeks after acute brain injury. Still, the actual clinical efficacy is not satisfactory. Electrophysiological interventions such as deep brain stimulation[10], TMS[11], transcranial direct current stimulation (tDCS)[12,13], low-frequency focused ultrasound[14], vagus nerve electrical stimulation (VNS)[15] and spinal cord stimulation (SCS)[16], may promote recovery in patients with subacute to chronic DoC in some trials with varying effects, but it seems difficult to get consistent evidence of efficacy in different study populations. For example, tDCS was shown to benefit patients with MCS in a randomized controlled trial in 2014[12], but was recently confirmed to be of no benefit in either MCS or UWS[13].

**Transcranial magnetic stimulation: a feasible treatment approach**

Transcranial magnetic stimulation is widely considered to be a safe, convenient and effective method to induce excitability changes in brain activity. Repetitive transcranial magnetic stimulation (rTMS) can modulate cortical activity via long-term potentiation and long-term depression to treat neurological and psychiatric disorders, such as Parkinson's disease, stroke, epilepsy, Alzheimer's disease, depression, schizophrenia, etc. [17,18]In recent ten years, the conscious promoting effect of rTMS in patients with MCS and/or UWS has been tested in clinical trials, mostly targeting the left M1, and the left or right dorsolateral prefrontal cortex (DLPFC) with high-frequency stimulation to produce a brain excitation effect and induce long-term potentiation-like plasticity.[19,11,17] M1 and DLPFC seem to have shown conscious restoring efficiency in some trials, but they lack consistent and convincing results in different study populations because the specific mechanism underlying the function is unclear. [20–23] A recent study showed that rTMS over the posterior parietal cortex (PPC) can be used to treat patients with DoC; however, the efficacy requires further studies.[22] Besides the high-frequency stimulation, iTBS, another more efficient and time-saving stimulation, has been used in a small sample of patients with DoC and resulted in both clinical

behavioral and neurophysiological improvement.[24] Importantly, no serious side effects have been reported in most previous studies.

**Cerebellum is a promising target for improving recovery of consciousness in patients with DoC**

According to the mesocircuit model, all severe brain injuries that cause leading to coma share a common pathological substrate, which is a marked loss of background synaptic activity owing to either widespread neocortical, striatal, and thalamic neuronal death, dysfunction or disconnection, or focal injury to the paramedian mesodiencephalon (i.e., the central thalamus and rostral brainstem tegmentum). Widespread disfacilitation occurs, involving neocortical, striatal, and thalamic neurons, with a specific contribution from central thalamic neurons that integrate the loss of input from multiple cerebral targets. Loss of medium spiny neurons inhibition of the globus pallidus interna (GPi) produces active inhibition of components of the central thalamus, including the central lateral nucleus. Together, these mechanisms are proposed to produce a downregulation of activity across the anterior forebrain, resulting in limited or fluctuating behavioral responsiveness.[25]

The cerebellum is well known for coordinating motor function and balance control; besides, it is also involved in non-motor functions, such as cognitive and executive functions, memory, learning, language, et al[26], and consciousness[27]. The cerebellar hemisphere is divided into 10 lobes, and the dentato-rubro-thalamic tract is the main tract connecting the cerebellum and cerebral cortex with lobe VII located in the posterior part as the cardinal afferent area[28]. Functional connectivity reveals that lobe VII is mainly mapped to frontoparietal network of cerebral cortex.

Considering the topography of the frontoparietal network in the cerebellum and the cortex-cerebellar pathway, lobe VII may be a candidate target to modulate the brain network in patients with DoC.

In the present study, we conduct a randomized, double-blind, cross-over trial to explore the consciousness-restoring efficacy and brain function-modulating effect of cerebellar iTBS in patients with DoC.

# 3 STUDY OBJECTIVES

## 3.1 Primary objective(s)

- To evaluate the effect of 5-session bilateral cerebellar iTBS stimulation on promoting consciousness recovery in patients with DoC.

## 3.2 Secondary objective(s)

- To evaluate the effects of cerebellar iTBS on EEG after five treatment sessions.
- To assess the functional outcomes of all the participants at 3 months and 6 months after enrolment.

## 3.3 Safety outcome(s)

Safety outcomes included adverse events, including intolerance to TMS, and severe adverse events. The common adverse effects of TMS include dizziness, headache, tinnitus, and tingling or burning sensation on the scalp, which usually ease spontaneously without symptomatic treatment. Any uncomfortable condition due to the iTBS stimulation and needs to be eased by down-regulating stimulus intensity or discontinuation of stimulation are seen as intolerance. Rare adverse effect of TMS includes seizures, for which physicians will administer appropriate symptomatic treatment.

A Serious Adverse Event (SAE) is defined as any event that:

- requires inpatient treatment not envisioned in the protocol or extends a current hospital stay;
- results in permanent or significant incapacity or disability;
- is life-threatening or results in death;
- causes a congenital anomaly or birth defect.

# 4 STUDY DESIGN

## 4.1 General study design

This is a randomized, double-blind, crossover trial. Patients will be randomly assigned to the iTBS stimulation-first group or the sham stimulation-first group after the 5-day baseline assessments (see section 7). During each treatment session, patients will receive either iTBS stimulation or sham stimulation once daily for 5 consecutive days. The two treatment sessions are separated by a 5-day washout period. Demographic, MRI, and clinical information of the patients will be collected at enrolment. The CRS-R score will be assessed before and after every session of iTBS and sham stimulation within 2 hours. 32-channel EEG will be performed 20 minutes before and after the first and after the fifth treatment sessions within 30 minutes. The analyses will compare the effects of iTBS and sham stimulation on the CRS-R score and EEG and assess the functional outcomes of all the participants 3 months and 6 months after enrolment. The TMS deliverer is the only investigator who is aware of the grouping results and is required to sign the confidentiality agreement. All other investigators, including attending physicians, nurses, clinical score raters, EEG analysts, and statisticians, are blinded to the allocation.

# 5 RANDOMIZATION

Once informed consent has been provided, the investigator should obtain the result of the randomization. Based on the random number table without stratification or blocks, patients will be assigned to one of the two intervention groups (1:1 ratio): iTBS stimulation-first or sham stimulation-first group.

# 6 STUDY POPULATION

## 6.1 Eligibility criteria

**Inclusion Criteria:**

- VS/UWS or MCS according to at least two CRS-R assessments
- Age ⩾18 years
- Written informed consent obtained from the legal surrogate

**Exclusion Criteria:**

- Patients in coma
- Brain injury <1 week
- Presence of metallic hardware in close contact with the discharging coil (such as cochlear implants, an Internal Pulse Generator or medication pumps)
- Patients with high risks according to standard questionnaire to screen transcranial magnetic stimulation (TMS) candidates[29]

## 6.2 Recruitment and screening

All patients will be recruited and pre-screened by an experienced neurologist based on the inclusion and exclusion criteria. Caregivers of potential candidates will be informed about the trial, and eligible patients will be transferred to the Coma Awakening Center for a comprehensive baseline assessment and further eligibility verification.

## 6.3 Assignment to study groups

After the baseline assessment and final eligibility check, the study patients will be randomly allocated by an investigator who will not be involved in the treatment, data analysis and follow-up.

# 7 STUDY INTERVENTION

## 7.1 Rest motor threshold determination

Bilateral RMTs are assessed prior to the initial administration of either active or sham stimulation on the same day, using single pulses delivered via an eight-figure coil positioned over the corresponding hemisphere of the patient's right and left hands, respectively. This is reported by the lowest intensity that elicits motor evoked potential $\geq 50\ \mu V$ in at least 5 of 10 consecutive stimulations.[30] Given the abnormal corticospinal excitability associated with various brain injuries in some patients with DoC, RMT may not be determinable in some patients or on certain sides.[31] For patients with only one side of RMT detectable, the intensity of iTBS is based on this detectable RMT. For patients with both sides of RMT undetectable, 50 % of maximal stimulator output will be used as RMT, as previously described.[31]

## 7.2 iTBS stimulation

The iTBS stimulation will be delivered by targeting the VII lobule of the cerebellum.[32] Structural MRI scans will be performed and uploaded to a neuronavigation system (Quicks Vision, Yingchi, Shenzhen, China) to precisely localize the VII lobe of the cerebellum for each participant based on the MR atlas[33]. During stimulation, the position and orientation of the coil will be localized and maintained through the neuronavigation system. Cerebellar iTBS will be delivered with a double-cone coil in 90 mm diameter connected to the TMS device (M-100 Ultimate, Yingchi, Shenzhen, China). The stimulation intensity will be set at 90% of the RMT of the contralateral primary motor cortex, adjusted according to the individual scalp-to-cortex distance.[34] The iTBS pattern is 3 pulses at 50 Hz repeated at 5 Hz, and a 2 s train of TBS was repeated every 10 s, for a total of 200 s (600 pulses)[35]. During each stimulation session, the left and right hemispheres of the cerebellum are sequentially stimulated, separated by 5-min interval.[36] Sham stimulation will be delivered by rotating the coil 90 degrees from the scalp over the targeting position. All patients wear earplugs during stimulation to block out auditory interference.

## 7.3 Sham stimulation

In the sham intervention phase, all participants undergo the same procedure as the active intervention, except that the coil is rotated 90 degrees from the scalp over the target position.

## 7.4 Concomitant treatments

During the study period, all participants will receive routine neurocritical care management. Medications that may have potential influences on cortical excitability, such as sedative drugs, are suspended from baseline assessment until the end of the last EEG and behavior assessments to avoid interference with the evaluation of EEG and consciousness. [35]

# 8 STUDY ASSESSMENTS

## 8.1 Level of consciousness

Two qualified and independent neurologists who are blinded to the patient allocation evaluate the CRS-R scores.[37] The CRS-R is subdivided into six subscales, including auditory, visual, motor, oromotor/verbal, communication, and arousal, for a total of 23 points to assess both the conscious awareness and arousal aspects of DoC patients. Diagnoses of VS/UWS and MCS (minus or plus) are determined based on the presence of specific items during subscale assessments.[38] The baseline consciousness is determined by the highest score obtained from at least two CRS-R assessments during enrollment.

## 8.2 Functional outcome assessment

GOS-E is utilized to evaluate the functional outcome 3 and 6 months after treatment, with a score of ≥ 4 indicating a favorable outcome and < 4 indicating an unfavorable outcome. [39,40] It will be conducted by another trained and independent evaluator through structured phone interviews.

## 8.3 EEG recording and analysis

### 8.3.1 EEG recordings

EEG signals are recorded using 32 Ag/AgCl ring electrodes connected to an amplifier system (BrainAmp MR Plus, Brain Products GmbH, Gilching, Germany). The electrodes are positioned according to the international 10-20 system. The EEG data acquisition occurs at a sampling rate of 1000 Hz, with impedance kept below 5 kΩ. EEG data is continuously monitored for 20 minutes, within 30 minutes before and after the first stimulation session, as well as after the fifth stimulation session. Patients are lying on the beds, awake with their eyes open. An arousal-promoting protocol will be implemented if their eyes are closed as previously described.[37]

### 8.3.2 EEG data pre-processing

Pre-processing and all subsequent analyses are carried out in MATLAB 2023a using the EEGLAB toolbox (https://sccn.ucsd.edu/eeglab/index.php). First, the recorded EEG data, with a sample frequency of 5000 Hz, is downsampled to 500 Hz. Next, the data are processed using a notch filter (48-52 Hz) and a band-pass filter (0.5–70 Hz). Following this, EEG segments with obvious artifacts are manually removed. Then, channels removed due to noise are interpolated with cleaned data, and independent component analysis (ICA) is performed to remove excessive eye movements and movement artifacts. Finally, each channel is re-referenced to the average of all channels.

### 8.3.3 Power spectra density and EEG pattern categorization

Power spectral density (PSD) and EEG pattern categorization are analyzed to evaluate the neurophysiological efficacy of five consecutive sessions and a single session of iTBS simulation targeting the bilateral cerebellum.

We estimate the power spectrum using Welch's method by segmenting EEG signals into equal-sized 5-second signals.[41] Each 5-second signal is then further segmented into sub-bands with a 50% overlap rate between adjacent sub-bands using a sliding window. Meanwhile, the Fast Fourier Transform (FFT) is applied to the overlapping segments and averaged in the frequency domain to reduce noise.

We generate PSD plots for each trial over the 1 to 70 Hz frequency range. Consistent with previous studies, the data are analyzed in six different frequency bands: delta (1-4 Hz), theta (4-8 Hz), alpha (8-13 Hz), beta (13-30 Hz), low gamma (30-50 Hz), and high gamma (50-70 Hz).

"ABCD" EEG pattern analysis is based on the power spectrum density across different frequency bands, which decodes the integrity of thalamocortical circuitry and the preservation of consciousness[42]. The "ABCD" EEG patterns are defined as follows: (1) Pattern A refers to the complete loss of thalamocortical integrity, and only delta frequency (< 4 Hz) exists; (2) Pattern B refers to a narrow oscillation of layer V pyramidal cells in the theta frequency range (4-7 Hz), which is due to the depolarization of neocortical neurons with depressed membrane potentials; (3) Pattern C refers to an oscillation of theta and beta frequencies resulting from the partial restoration of neocortical membrane potentials and coincident bursts of deafferented thalamic neurons; (4) Pattern D refers to the normal neocortical neuronal firing pattern at alpha and beta frequencies. Two qualified EEG technicians analyze the EEG independently, and disagreement is resolved by consulting a third EEG technician.

# 9 DATA MANAGEMENT AND QUALITY ASSURANCE

## 9.1 Confidentiality

All data will be handled using Microsoft Excel software. Detailed patient information is available solely for this trial and will not be used for any other purpose.

## 9.2 Data handling and source document identification

All trial data for each patient will be recorded from the source documents onto a pre-designed case report form (CRF) by a trained investigator, adhering to the following rules. All data should be legibly entered in black or blue-black ink using a ballpoint pen in regular script. In the event of an error, it should be crossed out with a single line to ensure the original entry remains legible. The correct entry should then be inserted clearly, and the alterations must be initialed and dated by the person making the correction. Rewriting or the use of correction fluid is not permitted. Participants must not be identified in the CRF by name or admission number. Instead, appropriate coded identification must be used in accordance with study-specific standard operating procedures.

## 9.3 Record keeping and archiving

The principal investigator (PI) is responsible for the secure archiving of trial documents and databases. All study-specific data and documents must be archived for a minimum of 10 years following the termination of the study. The data will be retained to facilitate potential additional retrospective subgroup analyses or long-term follow-up studies.

# 10 SAFETY

## 10.1 Definitions of safety outcomes

Safety outcomes included adverse events and severe adverse events. The common

adverse effects of TMS include dizziness, headache, tinnitus, and tingling or burning sensation on the scalp, which usually ease spontaneously without symptomatic treatment. Any uncomfortable condition due to the iTBS stimulation and needs to be eased by down-regulating stimulus intensity or discontinuation of stimulation are seen as intolerance. Rare adverse effect of TMS includes seizures, for which physicians will administer appropriate symptomatic treatment.

A Serious Adverse Event (SAE) is defined as any event that:

- requires inpatient treatment not envisioned in the protocol or extends a current hospital stay;
- results in permanent or significant incapacity or disability;
- is life-threatening or results in death;
- causes a congenital anomaly or birth defect.

## 10.2 Recording and reporting

Intolerance conditions, as well as seizures, will be dealt with by down-regulating the stimulus intensity, termination of stimulation, or symptomatic management if necessary. During the intervention phase of the study, all SAEs that cannot be excluded as being related to the intervention and intolerance conditions will be collected, fully investigated, and documented in source documents and CRFs. It is not expected that SAEs related to the intervention will occur after the intervention. In addition, all such events will be reported to the PI and the Data and Safety Monitoring Board (DSMB) as soon as possible (within 24 hours). The DSMB will closely monitor all SAEs for any relationship to the study procedures and protocol and will submit all SAEs to the independent statistician for review.

## 10.3 Follow up

SAEs that cannot be excluded as attributable to the intervention under investigation will be monitored until resolution or stabilization. Participants who experience ongoing SAEs at the time of study termination will undergo further follow-up until recovery or stabilization of the condition post-termination.

# 11 END OF THE TRIAL AND WITHDRAWAL RULES

## 11.1 Definition of end of the trial

The end of the trial will be the completion of the last visit for the last participant.

## 11.2 Rules for withdrawal of participants

The participation of individuals in either group should be discontinued if any of the following occur:

- SAEs, as determined by the DSMB, are deemed to be probably related to the trial protocol.

- The investigator believes that it is in the subject's best interest to withdraw from the trial.
- The legal representative of the patient chooses to withdraw consent for participation in the study.

# 12 STUDY ADMINISTRATIVE STRUCTURE

## 12.1 Principal Investigator (PI)

- Wen Jiang
  Department of Neurology
  Xijing Hospital, Fourth Military Medical University
  Xi'an, 710032, China
  Tel: +86-29-84771319
  Email: jiangwen@fmmu.edu.cn

## 12.2 Academic Committee (AC)

- Wen Jiang, Department of Neurology, Xijing Hospital, Fourth Military Medical University, Shaanxi, China
- Changgeng Song, Department of Neurology, Xijing Hospital, Fourth Military Medical University, Shaanxi, China
- Haibo Di, International Unresponsive Wakefulness Syndrome and Consciousness Science Institute, Hangzhou Normal University, Hangzhou, China
- Xiaogang Kang, Department of Neurology, Xijing Hospital, Fourth Military Medical University, Shaanxi, China

## 12.3 Data and Safety Monitoring Board (DSMB)

- Fang Yang, Department of Neurology, Xijing Hospital, Fourth Military Medical University, Shaanxi, China
- Chen Ma, Department of Neurology, Xijing Hospital, Fourth Military Medical University, Shaanxi, China
- Le Wang, Department of Neurology, Xijing Hospital, Fourth Military Medical University, Shaanxi, China
- Chen Li, Department of Statistics, Fourth Military Medical University, Shaanxi, China

## 12.4 Executive Committee (EC)

**Project Manager**

- Xiaogang Kang, Department of Neurology, Xijing Hospital, Fourth Military Medical University, Shaanxi, China

**Project Assistant**

- Rong Chen, Department of Neurology, Xijing Hospital, Fourth Military

Medical University, Shaanxi, China

**Data Manager**

- Jingjing Zhao, Department of Neurology, Xijing Hospital, Fourth Military Medical University, Shaanxi, China
- Xiaona Li, Department of Neurology, Xijing Hospital, Fourth Military Medical University, Shaanxi, China
- Jiheng He, Department of Neurology, Xijing Hospital, Fourth Military Medical University, Shaanxi, China

**Follow-up personnel**

- Luojun Wang, Department of Neurology, Xijing Hospital, Fourth Military Medical University, Shaanxi, China
- Dan Mi, Department of Neurology, Xijing Hospital, Fourth Military Medical University, Shaanxi, China

**Clinical Endpoint Event Arbitration Board**

- Wen Li, Department of Neurology, Xijing Hospital, Fourth Military Medical University, Shaanxi, China
- Rui Li, Department of Neurology, Xijing Hospital, Fourth Military Medical University, Shaanxi, China
- Qiong Gao, Department of Neurology, Xijing Hospital, Fourth Military Medical University, Shaanxi, China

**Statistician**

- Ling Wang, Department of Health Statistics, Fourth Military Medical University, Shaanxi, China
- Dianwei Wu, Department of Neurology, Xijing Hospita, Fourth Military Medical University, Shaanxi, China

**Finance Department**

- Yuan Che, Department of Neurology, Xijing Hospital, Fourth Military Medical University, Shaanxi, China

# 13 STATISTICAL CONSIDERATIONS

## 13.1 Determination of Sample Size

We determined that enrolling 44 patients would provide 90% power to detect a mean difference of 1.2 points (standard deviation [SD] = 1.5) in CRS-R changes between the active and sham stimulation groups, using a two-sided α of 0.05 and accounting for a 15% dropout rate. The anticipated 1.2 ± 1.5-point difference was conservatively estimated based on previous studies.[12,22,23,43]

## 13.2 Planned Analyses

Continuous variables will be expressed as mean ± standard deviation (SD) or median (interquartile range, IQR), and categorical variables will be expressed as percentages. Baseline continuous variables will be analyzed using Student's t-test for normal distribution and Mann - Whitney U test for skewed distribution. Categorical variables

will be analyzed using $\chi 2$ test analysis and Fisher's exact tests, when appropriate.

The between-group differences in the change of CRS-R total and subscale scores after the fifth treatment sessions will be analyzed using a linear mixed model (LMM) with fixed effects (sequence, period, time since injury, etiology, and age) and random effects (subjects for repeated measurements).[5–7]

The effects of cerebellar iTBS on CRS-R total scores will be further analyzed in subgroups of patients according to their baseline characteristics, i.e., VS/UWS and MCS, as well as anoxia etiology and non-anoxia etiology.

The between-group differences in PSD after the fifth treatment session will also be analyzed via LMM, incorporating baseline PSD as a fixed effect alongside other fixed and random variables as above.

The cumulative link mixed effects model (CLMM), which incorporated fixed and random effects as those of LMM (fixed effects: sequence, period, time since injury, etiology, and age; random effects: subjects for repeated measurements) and added baseline "ABCD" EEG patterns as fixed effects, will be used to analyze the between-group differences in "ABCD" EEG patterns after the fifth treatments.[8–10]

The analysis will be conducted in the intention-to-treat (ITT) populations and repeated in the per-protocol (PP) populations to test the robustness. For the ITT population, missing data will be imputed using the last observation carried forward method. EEG analyses wil be performed in patients with valid EEG data, and adjustments for multiple comparisons will be conducted using the false discovery rate method.[44] Two-sided P-values $< 0.05$ were considered statistically significant. All statistical analyses will be performed using PASS 20.0 (NCSS, LLC, Kaysville, UT, USA), R version 4.3.0 and SPSS version 26 (SPSS Inc., Chicago, IL, USA).

## 13.3 Handling of missing data and drop-outs

For the ITT population, missing data are imputed using the LOCF method. The patients who drop out before the cessation of the last intervention will be excluded from the PP analyses.

# 14 QUALITY ASSURANCE AND CONTROL

## 14.1 Independent oversight organization

The DSMB is responsible for assessing clinical care, investigating AEs, and determining whether a reported AE relates to the intervention and is relevant to the trial. The members of the DSMB will not be directly involved in the trial.

## 14.2 Training of the study team

Before the trial begins, all personnel involved, including the intervention operator, CRS-R evaluator, follow-up staff, and clinical physicians, will receive systematic training on the protocol.

## 14.3 Monitoring of study procedures

The PI is responsible for quality control, ensuring the trial follows predefined protocols, guidelines, and regulations. The PI will review informed consent forms, eligibility criteria, abnormal data, CRFs, and serious adverse events after every four patients have been randomized.

# 15 ETHICAL ASPECTS

## 15.1 Study registration

Once approved by the Ethics Committee (EC), the study will be registered on www.clinicaltrial.gov registry.

## 15.2 Ethical Conduct of the Study

Before conducting the study, we submit the protocol, consent forms, and other study-specific documents to the EC of Xijing Hospital and obtain formal approval. Any amendments to the protocol must be approved again by the EC. The trial will be conducted in accordance with the protocol and the principles of the current version of the Declaration of Helsinki and Good Clinical Practice.

## 15.3 Patient Information and Informed Consent

Considering that the patients we plan to enroll do not have the ability to give informed consent, we obtain all consent from the patients' representatives after thoroughly informing them of the study design, possible risks and benefits, costs, privacy measures, and alternative treatments should the representatives choose not to participate in the trial. Additionally, the representatives have the right to withdraw from the trial at any stage, and there will be no punishment or loss of benefits as a result.

## 15.4 Participant privacy and confidentiality

All the research data collected in this study will be kept confidential at Xijing Hospital; in order to protect the patients' identities, uniform format numbers will be assigned to participants in place of their names in study documents. Any information that could potentially identify the patients will be removed from all subjects' information collected, ensuring that the information cannot be linked to a specific study subject.

## 15.5 Protocol amendments

Any important protocol modifications will be submitted for approval to the EC and updated in the clinical trials registry after discussion and agreement among study team members. If necessary, these protocol amendments will inform the legal representatives of patients who are already enrolled. All non-substantial amendments will be communicated to the EC within the Annual Safety Report.

# 16 PUBLICATION AND DISSEMINATION POLICY

Upon completion of the study, the results of the present study can be communicated through abstracts presented at national or international conferences. Scientific papers will be written by the study team and submitted to peer-reviewed scientific journals. All authors must contribute substantially to the paper, including at least a detailed critical review.

# 17 FUNDING

National Natural Science Foundation of China (82441054), Shaanxi Province Special Support Program for Leading Talents in Scientific and Technological Innovation (tzihjw), and Clinical Research Project of the Fourth Military Medical University (2023LC2314).

# 18 APPENDICES

**JFK COMA RECOVERY SCALE ©2004**
**Record Form**

| | |
|---|---|
| **Patient:** **Date:** | |
| **Study period** | |
| **AUDITORY FUNCTION SCALE** | |
| 4 – Consistent Movement to Command* | |
| 3 – Reproducible Movement to Command* | |
| 2 – Localization to Sound | |
| 1 – Auditory Startle | |
| 0 – None | |
| **VISUAL FUNCTION SCALE** | |
| 5 – Object Recognition* | |
| 4 – Object localization: Reaching* | |
| 3 – Visual Pursuit* | |
| 2 – Fixation* | |
| 1 – Visual Startle | |
| 0 – None | |
| **MOTOR FUNCTION SCALE** | |
| 6 – Functional Object Use† | |
| 5 – Automatic Motor Response* | |
| 4 – Object Manipulation* | |
| 3 – Localisation to Noxious Stimulation* | |
| 2 – Flexion Withdrawal | |
| 1 – Abnormal Posturing | |
| 0 – None | |
| **OROMOTOR/VERBAL FUNCTION SCALE** | |
| 3 – Intelligible Verbalization* | |
| 2 – Vocalization/Oral Movement | |
| 1 – Oral Reflexive Movement | |
| 0 – None | |
| **COMMUNICATION SCALE** | |
| 2 – Functional: Accurate† | |
| 1 – Non-functional: Intentional* | |
| 0 – None | |
| **AROUSAL SCALE** | |
| 3 – Attention* | |
| 2 – Eye Opening w/o Stimulation | |
| 1 – Eye Opening with Stimulation | |
| 0 – Unarousable | |
| **TOTAL SCORE** | |

Abbreviation: w/o, without.

*Denotes MCS.

†Denotes emergence from MCS.

# 19 REFERENCES

(1) Edlow, B. L.; Claassen, J.; Schiff, N. D.; Greer, D. M. Recovery from Disorders of Consciousness: Mechanisms, Prognosis and Emerging Therapies. *Nat Rev Neurol* **2021**, *17* (3), 135–156. https://doi.org/10.1038/s41582-020-00428-x.

(2) Laureys, S.; Celesia, G. G.; Cohadon, F.; Lavrijsen, J.; León-Carrión, J.; Sannita, W. G.; et al. Unresponsive Wakefulness Syndrome: A New Name for the Vegetative State or Apallic Syndrome. *BMC Med* **2010**, *8*, 68. https://doi.org/10.1186/1741-7015-8-68.

(3) Giacino, J. T.; Ashwal, S.; Childs, N.; Cranford, R.; Jennett, B.; Katz, D. I.; et al. The Minimally Conscious State: Definition and Diagnostic Criteria. *Neurology* **2002**, *58* (3), 349–353. https://doi.org/10.1212/wnl.58.3.349.

(4) Fridman, E. A.; Schiff, N. D. Neuromodulation of the Conscious State Following Severe Brain Injuries. *Curr Opin Neurobiol* **2014**, *29*, 172–177. https://doi.org/10.1016/j.conb.2014.09.008.

(5) Giacino, J. T.; Whyte, J.; Bagiella, E.; Kalmar, K.; Childs, N.; Khademi, A.; et al. Placebo-Controlled Trial of Amantadine for Severe Traumatic Brain Injury. *N Engl J Med* **2012**, *366* (9), 819–826. https://doi.org/10.1056/NEJMoa1102609.

(6) Caliendo, E.; Lowder, R.; McLaughlin, M. J.; Watson, W. D.; Baum, K. T.; Blackwell, L. S.; et al. The Use of Methylphenidate During Inpatient Rehabilitation After Pediatric Traumatic Brain Injury: Population Characteristics and Prescribing Patterns. *J Head Trauma Rehabil* **2024**, *39* (3), E122–E131. https://doi.org/10.1097/HTR.0000000000000889.

(7) Krimchansky, B.-Z.; Keren, O.; Sazbon, L.; Groswasser, Z. Differential Time and Related Appearance of Signs, Indicating Improvement in the State of Consciousness in Vegetative State Traumatic Brain Injury (VS-TBI) Patients after Initiation of Dopamine Treatment. *Brain Inj* **2004**, *18* (11), 1099–1105. https://doi.org/10.1080/02699050310001646206.

(8) Passler, M. A.; Riggs, R. V. Positive Outcomes in Traumatic Brain Injury-Vegetative State: Patients Treated with Bromocriptine. *Arch Phys Med Rehabil* **2001**, *82* (3), 311–315. https://doi.org/10.1053/apmr.2001.20831.

(9) Fridman, E. A.; Krimchansky, B. Z.; Bonetto, M.; Galperin, T.; Gamzu, E. R.; Leiguarda, R. C.; et al. Continuous Subcutaneous Apomorphine for Severe Disorders of Consciousness after Traumatic Brain Injury. *Brain Inj* **2010**, *24* (4), 636–641. https://doi.org/10.3109/02699051003610433.

(10) Schiff, N. D. Central Thalamic Contributions to Arousal Regulation and Neurological Disorders of Consciousness. *Ann N Y Acad Sci* **2008**, *1129*, 105–118.

https://doi.org/10.1196/annals.1417.029.

(11) Yang, Z.; Yue, T.; Zschorlich, V. R.; Li, D.; Wang, D.; Qi, F. Behavioral Effects of Repetitive Transcranial Magnetic Stimulation in Disorders of Consciousness: A Systematic Review and Meta-Analysis. *Brain Sci* **2023**, *13* (10). https://doi.org/10.3390/brainsci13101362.

(12) Thibaut, A.; Bruno, M.-A.; Ledoux, D.; Demertzi, A.; Laureys, S. tDCS in Patients with Disorders of Consciousness: Sham-Controlled Randomized Double-Blind Study. *Neurology* **2014**, *82* (13), 1112–1118. https://doi.org/10.1212/WNL.0000000000000260.

(13) Thibaut, A.; Fregni, F.; Estraneo, A.; Fiorenza, S.; Noe, E.; Llorens, R.; et al. Sham-Controlled Randomized Multicentre Trial of Transcranial Direct Current Stimulation for Prolonged Disorders of Consciousness. *Eur J Neurol* **2023**. https://doi.org/10.1111/ene.15974.

(14) Cain, J. A.; Spivak, N. M.; Coetzee, J. P.; Crone, J. S.; Johnson, M. A.; Lutkenhoff, E. S.; et al. Ultrasonic Thalamic Stimulation in Chronic Disorders of Consciousness. *Brain Stimul* **2021**, *14* (2), 301–303. https://doi.org/10.1016/j.brs.2021.01.008.

(15) Corazzol, M.; Lio, G.; Lefevre, A.; Deiana, G.; Tell, L.; André-Obadia, N.; et al. Restoring Consciousness with Vagus Nerve Stimulation. *Curr Biol* **2017**, *27* (18), R994–R996. https://doi.org/10.1016/j.cub.2017.07.060.

(16) Piedade, G. S.; Assumpcao de Monaco, B.; Guest, J. D.; Cordeiro, J. G. Review of Spinal Cord Stimulation for Disorders of Consciousness. *Curr Opin Neurol* **2023**, *36* (6), 507–515. https://doi.org/10.1097/WCO.0000000000001222.

(17) Klomjai, W.; Katz, R.; Lackmy-Vallée, A. Basic Principles of Transcranial Magnetic Stimulation (TMS) and Repetitive TMS (rTMS). *Ann Phys Rehabil Med* **2015**, *58* (4), 208–213. https://doi.org/10.1016/j.rehab.2015.05.005.

(18) Lefaucheur, J.-P.; Aleman, A.; Baeken, C.; Benninger, D. H.; Brunelin, J.; Di Lazzaro, V.; et al. Evidence-Based Guidelines on the Therapeutic Use of Repetitive Transcranial Magnetic Stimulation (rTMS): An Update (2014-2018). *Clin Neurophysiol* **2020**, *131* (2), 474–528. https://doi.org/10.1016/j.clinph.2019.11.002.

(19) O'Neal, C. M.; Schroeder, L. N.; Wells, A. A.; Chen, S.; Stephens, T. M.; Glenn, C. A.; et al. Patient Outcomes in Disorders of Consciousness Following Transcranial Magnetic Stimulation: A Systematic Review and Meta-Analysis of Individual Patient Data. *Front Neurol* **2021**, *12*, 694970. https://doi.org/10.3389/fneur.2021.694970.

(20) Chen, J.-M.; Chen, Q.-F.; Wang, Z.-Y.; Chen, Y.-J.; Zhang, N.-N.; Xu, J.-W.; et al.

Influence of High-Frequency Repetitive Transcranial Magnetic Stimulation on Neurobehavioral and Electrophysiology in Patients with Disorders of Consciousness. *Neural Plast* **2022**, *2022*, 7195699. https://doi.org/10.1155/2022/7195699.

(21) Zhang, X.-H.; Han, P.; Zeng, Y.-Y.; Wang, Y.-L.; Lv, H.-L. The Clinical Effect of Repetitive Transcranial Magnetic Stimulation on the Disturbance of Consciousness in Patients in a Vegetative State. *Front Neurosci* **2021**, *15*, 647517. https://doi.org/10.3389/fnins.2021.647517.

(22) Fan, J.; Zhong, Y.; Wang, H.; Aierken, N.; He, R. Repetitive Transcranial Magnetic Stimulation Improves Consciousness in Some Patients with Disorders of Consciousness. *Clin Rehabil* **2022**, *36* (7), 916–925. https://doi.org/10.1177/02692155221089455.

(23) Shen, L.; Huang, Y.; Liao, Y.; Yin, X.; Huang, Y.; Ou, J.; et al. Effect of High-Frequency Repetitive Transcranial Magnetic Stimulation over M1 for Consciousness Recovery after Traumatic Brain Injury. *Brain Behav* **2023**, *13* (5), e2971. https://doi.org/10.1002/brb3.2971.

(24) Wu, M.; Wu, Y.; Yu, Y.; Gao, J.; Meng, F.; He, F.; et al. Effects of Theta Burst Stimulation of the Left Dorsolateral Prefrontal Cortex in Disorders of Consciousness. *Brain Stimul* **2018**, *11* (6), 1382–1384. https://doi.org/10.1016/j.brs.2018.07.055.

(25) Schiff, N. D. Recovery of Consciousness after Brain Injury: A Mesocircuit Hypothesis. *Trends Neurosci* **2010**, *33* (1), 1–9. https://doi.org/10.1016/j.tins.2009.11.002.

(26) Stoodley, C. J.; Schmahmann, J. D. Functional Topography in the Human Cerebellum: A Meta-Analysis of Neuroimaging Studies. *Neuroimage* **2009**, *44* (2), 489–501. https://doi.org/10.1016/j.neuroimage.2008.08.039.

(27) Zhu, J.; Chen, C.; Liu, X.; He, M.; Fang, Y.; Wang, L.; et al. Cerebellar Purkinje Cell Firing Promotes Conscious Recovery from Anesthesia State through Coordinating Neuronal Communications with Motor Cortex. *Theranostics* **2024**, *14* (2), 480–495. https://doi.org/10.7150/thno.89592.

(28) Ou, S.-Q.; Wei, P.-H.; Fan, X.-T.; Wang, Y.-H.; Meng, F.; Li, M.-Y.; et al. Delineating the Decussating Dentato-Rubro-Thalamic Tract and Its Connections in Humans Using Diffusion Spectrum Imaging Techniques. *Cerebellum* **2022**, *21* (1), 101–115. https://doi.org/10.1007/s12311-021-01283-2.

(29) Rossi, S.; Hallett, M.; Rossini, P. M.; Pascual-Leone, A. Safety, Ethical Considerations, and Application Guidelines for the Use of Transcranial Magnetic Stimulation in Clinical Practice and Research. *Clin Neurophysiol* **2009**, *120* (12),

2008–2039. https://doi.org/10.1016/j.clinph.2009.08.016.

(30) Rossini, P. M.; Burke, D.; Chen, R.; Cohen, L. G.; Daskalakis, Z.; Di Iorio, R.; et al. Non-Invasive Electrical and Magnetic Stimulation of the Brain, Spinal Cord, Roots and Peripheral Nerves: Basic Principles and Procedures for Routine Clinical and Research Application. An Updated Report from an I.F.C.N. Committee. *Clin Neurophysiol* **2015**, *126* (6), 1071–1107. https://doi.org/10.1016/j.clinph.2015.02.001.

(31) Lapitskaya, N.; Gosseries, O.; De Pasqua, V.; Pedersen, A. R.; Nielsen, J. F.; de Noordhout, A. M.; et al. Abnormal Corticospinal Excitability in Patients with Disorders of Consciousness. *Brain Stimul* **2013**, *6* (4), 590–597. https://doi.org/10.1016/j.brs.2013.01.002.

(32) Buckner, R. L. The Cerebellum and Cognitive Function: 25 Years of Insight from Anatomy and Neuroimaging. *Neuron* **2013**, *80* (3), 807–815. https://doi.org/10.1016/j.neuron.2013.10.044.

(33) Park, M. T. M.; Pipitone, J.; Baer, L. H.; Winterburn, J. L.; Shah, Y.; Chavez, S.; et al. Derivation of High-Resolution MRI Atlases of the Human Cerebellum at 3T and Segmentation Using Multiple Automatically Generated Templates. *Neuroimage* **2014**, *95*, 217–231. https://doi.org/10.1016/j.neuroimage.2014.03.037.

(34) Stokes, M. G.; Chambers, C. D.; Gould, I. C.; Henderson, T. R.; Janko, N. E.; Allen, N. B.; et al. Simple Metric for Scaling Motor Threshold Based on Scalp-Cortex Distance: Application to Studies Using Transcranial Magnetic Stimulation. *J Neurophysiol* **2005**, *94* (6), 4520–4527. https://doi.org/10.1152/jn.00067.2005.

(35) Vitello, M. M.; Rosenfelder, M. J.; Cardone, P.; Niimi, M.; Willacker, L.; Thibaut, A.; et al. A Protocol for a Multicenter Randomized and Personalized Controlled Trial Using rTMS in Patients with Disorders of Consciousness. *Front Neurol* **2023**, *14*, 1216468. https://doi.org/10.3389/fneur.2023.1216468.

(36) Rossi, S.; Hallett, M.; Rossini, P. M.; Pascual-Leone, A. Safety, Ethical Considerations, and Application Guidelines for the Use of Transcranial Magnetic Stimulation in Clinical Practice and Research. *Clin Neurophysiol* **2009**, *120* (12), 2008–2039. https://doi.org/10.1016/j.clinph.2009.08.016.

(37) Giacino, J. T.; Kalmar, K.; Whyte, J. The JFK Coma Recovery Scale-Revised: Measurement Characteristics and Diagnostic Utility. *Arch Phys Med Rehabil* **2004**, *85* (12), 2020–2029. https://doi.org/10.1016/j.apmr.2004.02.033.

(38) Kondziella, D.; Bender, A.; Diserens, K.; van Erp, W.; Estraneo, A.; Formisano, R.; et al. European Academy of Neurology Guideline on the Diagnosis of Coma and Other Disorders of Consciousness. *Eur J Neurol* **2020**, *27* (5), 741–756.

https://doi.org/10.1111/ene.14151.

(39) Egbebike, J.; Shen, Q.; Doyle, K.; Der-Nigoghossian, C. A.; Panicker, L.; Gonzales, I. J.; et al. Cognitive-Motor Dissociation and Time to Functional Recovery in Patients with Acute Brain Injury in the USA: A Prospective Observational Cohort Study. *Lancet Neurol* **2022**, *21* (8), 704–713. https://doi.org/10.1016/S1474-4422(22)00212-5.

(40) McCrea, M. A.; Giacino, J. T.; Barber, J.; Temkin, N. R.; Nelson, L. D.; Levin, H. S.; et al. Functional Outcomes Over the First Year After Moderate to Severe Traumatic Brain Injury in the Prospective, Longitudinal TRACK-TBI Study. *JAMA Neurol* **2021**, *78* (8), 982–992. https://doi.org/10.1001/jamaneurol.2021.2043.

(41) Welch, P. D. The Use of Fast Fourier Transform for the Estimation of Power Spectra: A Method Based on Time Averaging over Short, Modified Periodograms. *IEEE Trans. Audio & Electroacoust.* **1967**, *15* (2), 70–73. https://doi.org/10.1109/TAU.1967.1161901.

(42) Forgacs, P. B.; Frey, H.-P.; Velazquez, A.; Thompson, S.; Brodie, D.; Moitra, V.; et al. Dynamic Regimes of Neocortical Activity Linked to Corticothalamic Integrity Correlate with Outcomes in Acute Anoxic Brain Injury after Cardiac Arrest. *Ann Clin Transl Neurol* **2017**, *4* (2), 119–129. https://doi.org/10.1002/acn3.385.

(43) He, R. H.; Wang, H. J.; Zhou, Z.; Fan, J. Z.; Zhang, S. Q.; Zhong, Y. H. The Influence of High-Frequency Repetitive Transcranial Magnetic Stimulation on Endogenous Estrogen in Patients with Disorders of Consciousness. *Brain Stimul* **2021**, *14* (3), 461–466. https://doi.org/10.1016/j.brs.2021.02.014.

(44) Glickman, M. E.; Rao, S. R.; Schultz, M. R. False Discovery Rate Control Is a Recommended Alternative to Bonferroni-Type Adjustments in Health Studies. *J Clin Epidemiol* **2014**, *67* (8), 850–857. https://doi.org/10.1016/j.jclinepi.2014.03.012.

ClinicalTrials registration: NCT05558930

Effect of Cerebellar Stimulation on the Awareness Recovery in Disorders of Consciousness: A randomized, controlled, double-blind, cross-over trial

# CLINICAL STUDY PROTOCOL(Final)

Research site: Xijing Hospital

Principle Investigator: Wen Jiang

Responsible department: Department of Neurology

Contact: +86-29-84771319

Version 2.0-October 2022

# Table of Contents

STUDY SYNOPSIS ........ 31
ABBREVIATION ........ 33
1 STUDY FLOW CHART ........ 34
2 BACKGROUND ........ 35
3 STUDY OBJECTIVES ........ 36
3.1 Primary objective(s) ........ 36
3.2 Secondary objective(s) ........ 36
3.3 Safety outcome(s) ........ 37
4 STUDY DESIGN ........ 37
4.1 General study design ........ 37
5 RANDOMIZATION ........ 37
6 STUDY POPULATION ........ 38
6.1 Eligibility criteria ........ 38
6.2 Recruitment and screening ........ 38
6.3 Assignment to study groups ........ 38
7 STUDY INTERVENTION ........ 38
7.1 Rest motor threshold determination ........ 38
7.2 iTBS stimulation ........ 39
7.3 Sham stimulation ........ 39
7.4 Concomitant treatments ........ 39
8 STUDY ASSESSMENTS ........ 39
8.1 Level of consciousness ........ 39
8.2 Definition of consciousness improvement and unimprovement ........ 40
8.3 Functional outcome assessment ........ 40
8.4 EEG recording and analysis ........ 40
8.4.1 EEG recordings ........ 40
8.4.2 EEG data pre-processing ........ 40
8.4.3 Power spectra density and EEG pattern categorization ........ 41
8.4.4 Definition of EEG responsiveness to cerebellar iTBS ........ 41
9 DATA MANAGEMENT AND QUALITY ASSURANCE ........ 41
9.1 Confidentiality ........ 41
9.2 Data handling and source document identification ........ 42
9.3 Record keeping and archiving ........ 42
10 SAFETY ........ 42
10.1 Definitions of safety outcomes ........ 42
10.2 Recording and reporting ........ 42
10.3 Follow up ........ 43
11 END OF THE TRIAL AND WITHDRAWAL RULES ........ 43
11.1 Definition of end of the trial ........ 43
11.2 Rules for withdrawal of participants ........ 43
12 STUDY ADMINISTRATIVE STRUCTURE ........ 43
12.1 Principal Investigator (PI) ........ 43

12.2 Academic Committee (AC) ..... 43
12.3 Data and Safety Monitoring Board (DSMB) ..... 44
12.4 Executive Committee (EC) ..... 44
13 STATISTICAL CONSIDERATIONS ..... 45
13.1 Determination of Sample Size ..... 45
13.2 Planned Analyses ..... 45
13.3 Handling of missing data and drop-outs ..... 46
14 QUALITY ASSURANCE AND CONTROL ..... 46
14.1 Independent oversight organization ..... 46
14.2 Training of the study team ..... 46
14.3 Monitoring of study procedures ..... 46
15 ETHICAL ASPECTS ..... 47
15.1 Study registration ..... 47
15.2 Ethical Conduct of the Study ..... 47
15.3 Patient Information and Informed Consent ..... 47
15.4 Participant privacy and confidentiality ..... 47
15.5 Protocol amendments ..... 47
16 PUBLICATION AND DISSEMINATION POLICY ..... 47
17 FUNDING ..... 48
18 REFERENCES ..... 48
19 APPENDICES ..... 54

# STUDY SYNOPSIS

| | |
|---|---|
| Sponsor / Sponsor-Investigator: | Xijing Hospital, The Forth Military Medical University-Prof. Wen Jiang, MD, PhD<br>*(investigator-initiated trial)* |
| Study Title: | Effect of cerebellar stimulation on the awareness recovery in disorders of consciousness |
| Short Title / Study ID: | CARE-DOC |
| Protocol Version and Date: | V2.0, October 2022 |
| Trial registration: | www.clinicaltrial.gov NCT05558930 |
| Study design: | A randomized, double-blind, sham-controlled, crossover trial |
| Objective(s): | To evaluate the effects of cerebellar intermittent theta burst stimulation (iTBS) in promoting consciousness recovery in patients with disorders of consciousness (DoC) |
| Primary outcome (s): | • The difference in the change of Coma Recovery Scale-Revised (CRS-R) scores between the iTBS and sham stimulation groups after the 5-session treatment |
| Secondary outcome (s): | • The between-group differences in the “ABCD” EEG patterns after the fifth session of stimulation.<br>• The Glasgow Outcome Scale-Extended (GOS-E) scores of all the participants after 3 months and 6 months of enrollment |
| Inclusion / Exclusion criteria: | Inclusion:<br>• Vegetative state (VS) / unresponsive wakefulness syndrome (UWS) or minimally conscious state (MCS) according to at least two CRS-R assessments<br>• Age ≥18 years<br>• Time since brain injury from 15 days to 1 year<br>• Written informed consent obtained from legal surrogates<br>Exclusion:<br>• With brain injury due to unknown etiology<br>• With a history of mental or psychiatric disorder before the brain injury<br>• With uncontrolled seizures or status epilepticus<br>• With unstable or deteriorating medical conditions<br>• Contraindications to magnetic resonance imaging (MRI)<br>• With a skull defect<br>• Contraindications to transcranial magnetic stimulation (TMS) |

| | |
|---|---|
| Measurements and procedures: | Eligible patients will receive bilateral cerebellar iTBS and sham stimulation once daily for 5 consecutive days, either iTBS stimulation first or sham stimulation first, separated by a 5-day washout period. Demographic, MRI and clinical information are collected at enrolment. The level of consciousness is assessed with CRS-R before and after each stimulation session within 2 hours.<br>Additionally, 32-channel EEG will be performed 20 minutes before and after the first and after the fifth treatment sessions within 30 minutes.<br>The analyses will compare the effects of iTBS and sham stimulation on the CRS-R score and EEG and assess the functional outcomes of all the participants 3 months and 6 months after enrolment. |
| Study Intervention: | Bilateral cerebellar iTBS stimulation under neuronavigation |
| Number of Participants | 44 |

# ABBREVIATION

| | |
|---|---|
| AC | Academic Committee |
| AE | adverse event |
| CLMM | cumulative link mixed effects model |
| CRF | case report form |
| CRS-R | Coma Recovery Scale-Revised |
| CLMM | cumulative link mixed effects model |
| DSMB | Data and Safety Monitoring Board |
| DoC | disorders of consciousness |
| DLPFC | dorsolateral prefrontal cortex |
| DSMB | Data and Safety Monitoring Board |
| EEG | electroencephalography |
| EMCS | emergence from MCS |
| EC | Ethics Committee |
| EC | Executive Committee |
| FFT | fast fourier transform |
| GLM | generalized linear model |
| GPi | globus pallidus interna |
| HF-rTMS | high-frequency rTMS |
| ICA | independent component analysis |
| ITT | intention-to-treat |
| iTBS | intermittent theta burst stimulation |
| IQR | interquartile range |
| LOCF | last observation carried forward |
| LMM | linear mixed model |
| MRI | magnetic resonance imaging |
| MCS | minimally conscious state |
| PP | per-protocol |
| PPC | posterior parietal cortex |
| PSD | power spectral density |
| PI | Principal Investigator |
| rTMS | repetitive transcranial magnetic stimulation |
| RMT | resting motor thresholds |
| SAE | serious adverse event |
| SAP | statistical analysis plan |
| SCS | spinal cord stimulation |
| SD | standard deviation |
| GOS-E | Glasgow Outcome Scale-Extended |
| tDCS | transcranial direct current stimulation |
| TMS | transcranial magnetic stimulation |
| TBI | traumatic brain injury |
| UWS | unresponsive wakefulness syndrome |
| VNS | vagus nerve electrical stimulation |
| VS | vegetative state |

# 1 STUDY FLOW CHART

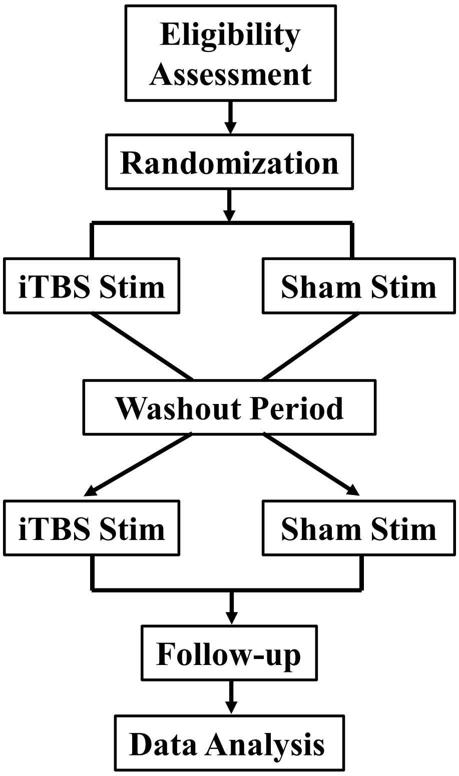


**Flow chart**: iTBS=intermittent transcranial magnetic stimulation; stim=stimulation

# 2 BACKGROUND

Disorders of consciousness (DoC) are characterized by alterations in arousal and/or awareness and are caused by severe brain injuries that disrupt the functional connectivity of corticothalamic system and its connection between basal ganglia and limbic system, to weaken or stop the excitatory synaptic activity in the cerebral cortex. Cardiac arrest, traumatic brain injury (TBI), intracerebral hemorrhage, and ischemic stroke are common causes of DoC.[1] Patients who survive coma after the acute stage of brain injury but still do not awaken often step into a phase of VS/UWS (arousal without awareness)[2] or minimally conscious state (MCS) (minimal, reproducible but inconsistent awareness)[3].

**Lack of evidently effective treatment for recovery of patients with DoC**

Pharmacological treatments that focused on promoting dopamine signaling[4], such as amantadine[5], methylphenidate[6], levodopa[7], bromocriptine[8], and subcutaneous apomorphine[9], have been tested in previous trials to promote recovery in patients with DoC. However, only amantadine has been confirmed to have some effect on restoring consciousness in patients with VS/UWS and MCS 4-16 weeks after acute brain injury. Still, the actual clinical efficacy is not satisfactory. Electrophysiological interventions such as deep brain stimulation[10], TMS[11], transcranial direct current stimulation (tDCS)[12,13], low-frequency focused ultrasound[14], vagus nerve electrical stimulation (VNS)[15] and spinal cord stimulation (SCS)[16], may promote recovery in patients with subacute to chronic DoC in some trials with varying effects, but it seems difficult to get consistent evidence of efficacy in different study populations. For example, tDCS was shown to benefit patients with MCS in a randomized controlled trial in 2014[12], but was recently confirmed to be of no benefit in either MCS or UWS[13].

**Transcranial magnetic stimulation: a feasible treatment approach**

Transcranial magnetic stimulation is widely considered to be a safe, convenient and effective method to induce excitability changes in brain activity. Repetitive transcranial magnetic stimulation (rTMS) can modulate cortical activity via long-term potentiation and long-term depression to treat neurological and psychiatric disorders, such as Parkinson's disease, stroke, epilepsy, Alzheimer's disease, depression, schizophrenia, etc. [17,18]In recent ten years, the conscious promoting effect of rTMS in patients with MCS and/or UWS has been tested in clinical trials, mostly targeting the left M1, and the left or right dorsolateral prefrontal cortex (DLPFC) with high-frequency stimulation to produce a brain excitation effect and induce long-term potentiation-like plasticity.[19,11,17] M1 and DLPFC seem to have shown conscious restoring efficiency in some trials, but they lack consistent and convincing results in different study populations because the specific mechanism underlying the function is unclear. [20–23] A recent study showed that rTMS over the posterior parietal cortex (PPC) can be used to treat patients with DoC; however, the efficacy requires further studies.[22] Besides the high-frequency stimulation, iTBS, another more efficient and time-saving stimulation, has been used in a small sample of patients with DoC and resulted in both clinical

behavioral and neurophysiological improvement.[24] Importantly, no serious side effects have been reported in most previous studies.

**Cerebellum is a promising target for improving recovery of consciousness in patients with DoC**

According to the mesocircuit model, all severe brain injuries that cause leading to coma share a common pathological substrate, which is a marked loss of background synaptic activity owing to either widespread neocortical, striatal, and thalamic neuronal death, dysfunction or disconnection, or focal injury to the paramedian mesodiencephalon (i.e., the central thalamus and rostral brainstem tegmentum). Widespread disfacilitation occurs, involving neocortical, striatal, and thalamic neurons, with a specific contribution from central thalamic neurons that integrate the loss of input from multiple cerebral targets. Loss of medium spiny neurons inhibition of the globus pallidus interna (GPi) produces active inhibition of components of the central thalamus, including the central lateral nucleus. Together, these mechanisms are proposed to produce a downregulation of activity across the anterior forebrain, resulting in limited or fluctuating behavioral responsiveness.[25]

The cerebellum is well known for coordinating motor function and balance control; besides, it is also involved in non-motor functions, such as cognitive and executive functions, memory, learning, language, et al[26], and consciousness[27]. The cerebellar hemisphere is divided into 10 lobes, and the dentato-rubro-thalamic tract is the main tract connecting the cerebellum and cerebral cortex with lobe VII located in the posterior part as the cardinal afferent area[28]. Functional connectivity reveals that lobe VII is mainly mapped to frontoparietal network of cerebral cortex.

Considering the topography of the frontoparietal network in the cerebellum and the cortex-cerebellar pathway, lobe VII may be a candidate target to modulate the brain network in patients with DoC.

In the present study, we conduct a randomized, double-blind, cross-over trial to explore the consciousness-restoring efficacy and brain function-modulating effect of cerebellar iTBS in patients with DoC.

# 3 STUDY OBJECTIVES

## 3.1 Primary objective(s)

- To evaluate the effect of 5-session bilateral cerebellar iTBS stimulation on promoting consciousness recovery in patients with DoC.

## 3.2 Secondary objective(s)

- To evaluate the effects of cerebellar iTBS on EEG after five treatment sessions.
- To assess the functional outcomes of all the participants at 3 months and 6 months after enrolment.

## 3.3 Safety outcome(s)

Safety outcomes included adverse events, including intolerance to TMS, and severe adverse events. The common adverse effects of TMS include dizziness, headache, tinnitus, and tingling or burning sensation on the scalp, which usually ease spontaneously without symptomatic treatment. Any uncomfortable condition due to the iTBS stimulation and needs to be eased by down-regulating stimulus intensity or discontinuation of stimulation are seen as intolerance. Rare adverse effect of TMS includes seizures, for which physicians will administer appropriate symptomatic treatment.

A Serious Adverse Event (SAE) is defined as any event that:

- requires inpatient treatment not envisioned in the protocol or extends a current hospital stay;
- results in permanent or significant incapacity or disability;
- is life-threatening or results in death;
- causes a congenital anomaly or birth defect.

# 4 STUDY DESIGN

## 4.1 General study design

This is a randomized, double-blind, crossover trial. Patients will be randomly assigned to the iTBS stimulation-first group or the sham stimulation-first group after the 5-day baseline assessments (see section 7). During each treatment session, patients will receive either iTBS stimulation or sham stimulation once daily for 5 consecutive days. The two treatment sessions are separated by a 5-day washout period. Demographic, MRI, and clinical information of the patients will be collected at enrolment. The CRS-R score will be assessed before and after every session of iTBS and sham stimulation within 2 hours. 32-channel EEG will be performed 20 minutes before and after the first and after the fifth treatment sessions within 30 minutes. The analyses will compare the effects of iTBS and sham stimulation on the CRS-R score and EEG and assess the functional outcomes of all the participants 3 months and 6 months after enrolment. The TMS deliverer is the only investigator who is aware of the grouping results and is required to sign the confidentiality agreement. All other investigators, including attending physicians, nurses, clinical score raters, EEG analysts, and statisticians, are blinded to the allocation.

# 5 RANDOMIZATION

Once informed consent has been provided, the investigator should obtain the result of the randomization. Based on the random number table without stratification or blocks, patients will be assigned to one of the two intervention groups (1:1 ratio): iTBS stimulation-first or sham stimulation-first group.

# 6 STUDY POPULATION

## 6.1 Eligibility criteria

**Inclusion Criteria:**

- VS/UWS or MCS according to at least two CRS-R assessments
- Age ⩾18 years
- Time since brain injury between 15 days and 1 year
- Written informed consent obtained from the legal surrogate

**Exclusion Criteria:**

- Brain injury due to unknown etiology
- History of mental or psychiatric disorder prior to the brain injury
- Uncontrolled seizures or status epilepticus
- Unstable or deteriorating medical condition
- Contraindications to MRI
- Skull defect
- Contraindications to TMS[29]

## 6.2 Recruitment and screening

All patients will be recruited and pre-screened by an experienced neurologist based on the inclusion and exclusion criteria. Caregivers of potential candidates will be informed about the trial, and eligible patients will be transferred to the Coma Awakening Center for a comprehensive baseline assessment and further eligibility verification.

## 6.3 Assignment to study groups

After the baseline assessment and final eligibility check, the study patients will be randomly allocated by an investigator who will not be involved in the treatment, data analysis and follow-up.

# 7 STUDY INTERVENTION

## 7.1 Rest motor threshold determination

Bilateral RMTs are assessed prior to the initial administration of either active or sham stimulation on the same day, using single pulses delivered via an eight-figure coil positioned over the corresponding hemisphere of the patient's right and left hands, respectively. This is indicated by a visually detectable twitch in the abductor pollicis brevis muscle.[31] Given the abnormal corticospinal excitability associated with various brain injuries in some patients with DoC, RMT may not be determinable in some patients or on certain sides.[32] For patients with only one side of RMT detectable, the intensity of iTBS is based on this detectable RMT. For patients with both sides of RMT undetectable, 50 % of maximal stimulator output will be used as RMT, as previously

described.[32]

## 7.2 iTBS stimulation

iTBS stimulation will be delivered by targeting the VII lobule of the cerebellum.[33] Structural MRI scans will be performed and uploaded to a neuronavigation system (Quicks Vision, Yingchi, Shenzhen, China) to precisely localize the VII lobe of the cerebellum for each participant based on the MR atlas[34]. During stimulation, the position and orientation of the coil will be localized and maintained through the neuronavigation system. Cerebellar iTBS will be delivered with a double-cone coil in 90 mm diameter connected to the TMS device (M-100 Ultimate, Yingchi, Shenzhen, China). The stimulation intensity will be set at 90% of the RMT of the contralateral primary motor cortex, adjusted according to the individual scalp-to-cortex distance.[35] The iTBS pattern is 3 pulses at 50 Hz repeated at 5 Hz, and a 2 s train of TBS was repeated every 10 s, for a total of 200 s (600 pulses)[31]. During each stimulation session, the left and right hemispheres of the cerebellum are sequentially stimulated, separated by 5-min interval.[36] Sham stimulation will be delivered by rotating the coil 90 degrees from the scalp over the targeting position. All patients wear earplugs during stimulation to block out auditory interference.

## 7.3 Sham stimulation

In the sham intervention phase, all participants undergo the same procedure as the active intervention, except that the coil is rotated 90 degrees from the scalp over the target position.

## 7.4 Concomitant treatments

During the study period, all participants will receive routine neurocritical care management. Medications that may have potential influences on cortical excitability, such as sedative drugs, are suspended from baseline assessment until the end of the last EEG and behavior assessments to avoid interference with the evaluation of EEG and consciousness. [31]

# 8 STUDY ASSESSMENTS

## 8.1 Level of consciousness

Two qualified and independent neurologists who are blinded to the patient allocation evaluate the CRS-R scores.[37] The CRS-R is subdivided into six subscales, including auditory, visual, motor, oromotor/verbal, communication, and arousal, for a total of 23 points to assess both the conscious awareness and arousal aspects of DoC patients. Diagnoses of VS/UWS and MCS (minus or plus) are determined based on the presence of specific items during subscale assessments.[38] The baseline consciousness is determined by the highest score obtained from at least two CRS-R assessments during enrollment. The consciousness level during the follow-up phase will be evaluated via

structured telephone interviews developed from CRS-R, either with patients themselves or close relatives.[39,40]

## 8.2 Definition of consciousness improvement and unimprovement

According to the 6-month consciousness, patients will be categorized into the improved consciousness group if they transition from UWS to MCS or emergence from MCS (EMCS), from MCS minus to MCS plus or EMCS, and from MCS plus to EMCS. Patients with unimproved consciousness are defined as those with a reduced or unchanged level of consciousness at six months. Patients with unimproved consciousness are defined as those with a reduced or unchanged level of consciousness. If patients die during the follow-up period, their consciousness will be evaluated based on their level of consciousness before death. For patients who die between 3 and 6 months after treatment, their pre-death consciousness will be assessed based on their condition at 3 months post-treatment. For those who die within 3 months of treatment, their pre-death consciousness will be evaluated according to their consciousness at the time of discharge.

## 8.3 Functional outcome assessment

GOS-E is utilized to evaluate the functional outcome 3 and 6 months after treatment, with a score of ≥ 4 indicating a favorable outcome and < 4 indicating an unfavorable outcome. [41,42] It will be conducted by another trained and independent evaluator through structured phone interviews.

## 8.4 EEG recording and analysis

### 8.4.1 EEG recordings

EEG signals are recorded using 32 Ag/AgCl ring electrodes connected to an amplifier system (BrainAmp MR Plus, Brain Products GmbH, Gilching, Germany). The electrodes are positioned according to the international 10-20 system. The EEG data acquisition occurs at a sampling rate of 1000 Hz, with impedance kept below 5 kΩ. EEG data is continuously monitored for 20 minutes, within 30 minutes before and after the first stimulation session, as well as after the fifth stimulation session. Patients are lying on the beds, awake with their eyes open. An arousal-promoting protocol will be implemented if their eyes are closed as previously described.[37]

### 8.4.2 EEG data pre-processing

Pre-processing and all subsequent analyses are carried out in MATLAB 2023a using the EEGLAB toolbox (https://sccn.ucsd.edu/eeglab/index.php). First, the recorded EEG data, with a sample frequency of 5000 Hz, is downsampled to 500 Hz. Next, the data are processed using a notch filter (48-52 Hz) and a band-pass filter (0.5–70 Hz).

Following this, EEG segments with obvious artifacts are manually removed. Then, channels removed due to noise are interpolated with cleaned data, and independent component analysis (ICA) is performed to remove excessive eye movements and movement artifacts. Finally, each channel is re-referenced to the average of all channels.

### 8.4.3 Power spectra density and EEG pattern categorization

Power spectral density (PSD) and EEG pattern categorization are analyzed to evaluate the neurophysiological efficacy of five consecutive sessions and a single session of iTBS simulation targeting the bilateral cerebellum.

We estimate the power spectrum using Welch's method by segmenting EEG signals into equal-sized 5-second signals.[43] Each 5-second signal is then further segmented into sub-bands with a 50% overlap rate between adjacent sub-bands using a sliding window. Meanwhile, the Fast Fourier Transform (FFT) is applied to the overlapping segments and averaged in the frequency domain to reduce noise.

We generate PSD plots for each trial over the 1 to 70 Hz frequency range. Consistent with previous studies, the data are analyzed in six different frequency bands: delta (1-4 Hz), theta (4-8 Hz), alpha (8-13 Hz), beta (13-30 Hz), low gamma (30-50 Hz), and high gamma (50-70 Hz).

"ABCD" EEG pattern analysis is based on the power spectrum density across different frequency bands, which decodes the integrity of thalamocortical circuitry and the preservation of consciousness[44]. The "ABCD" EEG patterns are defined as follows: (1) Pattern A refers to the complete loss of thalamocortical integrity, and only delta frequency (< 4 Hz) exists; (2) Pattern B refers to a narrow oscillation of layer V pyramidal cells in the theta frequency range (4-7 Hz), which is due to the depolarization of neocortical neurons with depressed membrane potentials; (3) Pattern C refers to an oscillation of theta and beta frequencies resulting from the partial restoration of neocortical membrane potentials and coincident bursts of deafferented thalamic neurons; (4) Pattern D refers to the normal neocortical neuronal firing pattern at alpha and beta frequencies. Two qualified EEG technicians analyze the EEG independently, and disagreement is resolved by consulting a third EEG technician.

### 8.4.4 Definition of EEG responsiveness to cerebellar iTBS

The EEG responsiveness to cerebellar iTBS is defined as patients who exhibited elevated levels of "ABCD" EEG patterns compared to the baseline after either the first or fifth session of iTBS stimulation.

# 9 DATA MANAGEMENT AND QUALITY ASSURANCE

## 9.1 Confidentiality

All data will be handled using Microsoft Excel software. Detailed patient information is available solely for this trial and will not be used for any other purpose.

## 9.2 Data handling and source document identification

All trial data for each patient will be recorded from the source documents onto a pre-designed case report form (CRF) by a trained investigator, adhering to the following rules. All data should be legibly entered in black or blue-black ink using a ballpoint pen in regular script. In the event of an error, it should be crossed out with a single line to ensure the original entry remains legible. The correct entry should then be inserted clearly, and the alterations must be initialed and dated by the person making the correction. Rewriting or the use of correction fluid is not permitted. Participants must not be identified in the CRF by name or admission number. Instead, appropriate coded identification must be used in accordance with study-specific standard operating procedures.

## 9.3 Record keeping and archiving

The principal investigator (PI) is responsible for the secure archiving of trial documents and databases. All study-specific data and documents must be archived for a minimum of 10 years following the termination of the study. The data will be retained to facilitate potential additional retrospective subgroup analyses or long-term follow-up studies.

# 10 SAFETY

## 10.1 Definitions of safety outcomes

Safety outcomes included adverse events, including intolerance to TMS, and severe adverse events. The common adverse effects of TMS include dizziness, headache, tinnitus, and tingling or burning sensation on the scalp, which usually ease spontaneously without symptomatic treatment. Any uncomfortable condition due to the iTBS stimulation and needs to be eased by down-regulating stimulus intensity or discontinuation of stimulation are seen as intolerance. Rare adverse effect of TMS includes seizures, for which physicians will administer appropriate symptomatic treatment.

A Serious Adverse Event (SAE) is defined as any event that:

- requires inpatient treatment not envisioned in the protocol or extends a current hospital stay;
- results in permanent or significant incapacity or disability;
- is life-threatening or results in death;
- causes a congenital anomaly or birth defect.

## 10.2 Recording and reporting

Intolerance conditions, as well as seizures, will be dealt with by down-regulating the stimulus intensity, termination of stimulation, or symptomatic management if necessary. During the intervention phase of the study, all SAEs that cannot be excluded as being related to the intervention and intolerance conditions will be collected, fully

investigated, and documented in source documents and CRFs. It is not expected that SAEs related to the intervention will occur after the intervention. In addition, all such events will be reported to the PI and the Data and Safety Monitoring Board (DSMB) as soon as possible (within 24 hours). The DSMB will closely monitor all SAEs for any relationship to the study procedures and protocol and will submit all SAEs to the independent statistician for review.

## 10.3 Follow up

SAEs that cannot be excluded as attributable to the intervention under investigation will be monitored until resolution or stabilization. Participants who experience ongoing SAEs at the time of study termination will undergo further follow-up until recovery or stabilization of the condition post-termination.

# 11 END OF THE TRIAL AND WITHDRAWAL RULES

## 11.1 Definition of end of the trial

The end of the trial will be the completion of the last visit for the last participant.

## 11.2 Rules for withdrawal of participants

The participation of individuals in either group should be discontinued if any of the following occur:

- SAEs, as determined by the DSMB, are deemed to be probably related to the trial protocol.
- The investigator believes that it is in the subject's best interest to withdraw from the trial.
- The legal representative of the patient chooses to withdraw consent for participation in the study.

# 12 STUDY ADMINISTRATIVE STRUCTURE

## 12.1 Principal Investigator (PI)

- Wen Jiang
  Department of Neurology
  Xijing Hospital, Fourth Military Medical University
  Xi’an, 710032, China
  Tel: +86-29-84771319
  Email: jiangwen@fmmu.edu.cn

## 12.2 Academic Committee (AC)

- Wen Jiang, Department of Neurology, Xijing Hospital, Fourth Military

Medical University, Shaanxi, China
- Changgeng Song, Department of Neurology, Xijing Hospital, Fourth Military Medical University, Shaanxi, China
- Haibo Di, International Unresponsive Wakefulness Syndrome and Consciousness Science Institute, Hangzhou Normal University, Hangzhou, China
- Xiaogang Kang, Department of Neurology, Xijing Hospital, Fourth Military Medical University, Shaanxi, China

## 12.3 Data and Safety Monitoring Board (DSMB)

- Fang Yang, Department of Neurology, Xijing Hospital, Fourth Military Medical University, Shaanxi, China
- Chen Ma, Department of Neurology, Xijing Hospital, Fourth Military Medical University, Shaanxi, China
- Le Wang, Department of Neurology, Xijing Hospital, Fourth Military Medical University, Shaanxi, China
- Chen Li, Department of Statistics, Fourth Military Medical University, Shaanxi, China

## 12.4 Executive Committee (EC)

**Project Manager**
- Xiaogang Kang, Department of Neurology, Xijing Hospital, Fourth Military Medical University, Shaanxi, China

**Project Assistant**
- Rong Chen, Department of Neurology, Xijing Hospital, Fourth Military Medical University, Shaanxi, China

**Data Manager**
- Jingjing Zhao, Department of Neurology, Xijing Hospital, Fourth Military Medical University, Shaanxi, China
- Xiaona Li, Department of Neurology, Xijing Hospital, Fourth Military Medical University, Shaanxi, China
- Jiheng He, Department of Neurology, Xijing Hospital, Fourth Military Medical University, Shaanxi, China

**Follow-up personnel**
- Luojun Wang, Department of Neurology, Xijing Hospital, Fourth Military Medical University, Shaanxi, China
- Dan Mi, Department of Neurology, Xijing Hospital, Fourth Military Medical University, Shaanxi, China

**Clinical Endpoint Event Arbitration Board**
- Wen Li, Department of Neurology, Xijing Hospital, Fourth Military Medical University, Shaanxi, China
- Rui Li, Department of Neurology, Xijing Hospital, Fourth Military Medical University, Shaanxi, China

- Qiong Gao, Department of Neurology, Xijing Hospital, Fourth Military Medical University, Shaanxi, China

**Statistician**

- Ling Wang, Department of Health Statistics, Fourth Military Medical University, Shaanxi, China
- Dianwei Wu, Department of Neurology, Xijing Hospita, Fourth Military Medical University, Shaanxi, China

**Finance Department**

- Yuan Che, Department of Neurology, Xijing Hospital, Fourth Military Medical University, Shaanxi, China

# 13 STATISTICAL CONSIDERATIONS

## 13.1 Determination of Sample Size

We determined that enrolling 44 patients would provide 90% power to detect a mean difference of 1.2 points (standard deviation [SD] = 1.5) in CRS-R changes between the active and sham stimulation groups, using a two-sided α of 0.05 and accounting for a 15% dropout rate. The anticipated 1.2 ± 1.5-point difference was conservatively estimated based on previous studies.[12,22,23,45]

## 13.2 Planned Analyses

Continuous variables will be expressed as mean ± standard deviation (SD) or median (interquartile range, IQR), and categorical variables will be expressed as percentages. Baseline continuous variables will be analyzed using Student's t-test for normal distribution and Mann - Whitney U test for skewed distribution. Categorical variables will be analyzed using $\chi 2$ test analysis and Fisher's exact tests, when appropriate.

Besides the primary and secondary outcomes mentioned above, we will conduct a post-hoc analysis of the changes in CRS-R scores and EEG after the first treatment session. At three- and six-months post-treatment, the consciousness of patients will be additionally assessed based-on CRS-R via telephone interview.

The between-group differences in the change of CRS-R total and subscale scores after the first and fifth treatment sessions will be analyzed using a linear mixed model (LMM) with fixed effects (sequence, period, time since injury, etiology, and age) and random effects (subjects for repeated measurements).[46–48]

The effects of cerebellar iTBS on CRS-R total scores will be further analyzed in subgroups of patients according to their baseline characteristics, i.e., VS/UWS and MCS, as well as anoxia etiology and non-anoxia etiology.

The between-group differences in PSD after the first and fifth treatment sessions will also be analyzed via LMM, incorporating baseline PSD as a fixed effect alongside other fixed and random variables as above.

The cumulative link mixed effects model (CLMM), which incorporated fixed and random effects as those of LMM (fixed effects: sequence, period, time since injury, etiology, and age; random effects: subjects for repeated measurements) and added

baseline "ABCD" EEG patterns as fixed effects, will be used to analyze the between-group differences in "ABCD" EEG patterns after the first and fifth treatments.[49–51]We will use the ordinal logistic regression to explore the relationships between the baseline "ABCD" EEG patterns and the functional outcomes and recovery of consciousness six months post-treatment. To investigate the relationships between EEG responsiveness and six-month outcomes, we will use the generalized linear model (GLM), with adjustments made for baseline characteristics and time period.

The analysis will be conducted in the intention-to-treat (ITT) populations and repeated in the per-protocol (PP) populations to test the robustness. For the ITT population, missing data will be imputed using the last observation carried forward method. EEG analyses wil be performed in patients with valid EEG data, and adjustments for multiple comparisons will be conducted using the false discovery rate method.[52] Two-sided P-values < 0.05 were considered statistically significant. All statistical analyses will be performed using PASS 20.0 (NCSS, LLC, Kaysville, UT, USA), R version 4.3.0 and SPSS version 26 (SPSS Inc., Chicago, IL, USA).

## 13.3 Handling of missing data and drop-outs

For the ITT population, missing data are imputed using the LOCF method. The patients who drop out before the cessation of the last intervention will be excluded from the PP analyses.

# 14 QUALITY ASSURANCE AND CONTROL

## 14.1 Independent oversight organization

The DSMB is responsible for assessing clinical care, investigating AEs, and determining whether a reported AE relates to the intervention and is relevant to the trial. The members of the DSMB will not be directly involved in the trial.

## 14.2 Training of the study team

Before the trial begins, all personnel involved, including the intervention operator, CRS-R evaluator, follow-up staff, and clinical physicians, will receive systematic training on the protocol.

## 14.3 Monitoring of study procedures

The PI is responsible for quality control, ensuring the trial follows predefined protocols, guidelines, and regulations. The PI will review informed consent forms, eligibility criteria, abnormal data, CRFs, and serious adverse events after every four patients have been randomized.

# 15 ETHICAL ASPECTS

## 15.1 Study registration

Once approved by the Ethics Committee (EC), the study will be registered on www.clinicaltrial.gov registry.

## 15.2 Ethical Conduct of the Study

Before conducting the study, we submit the protocol, consent forms, and other study-specific documents to the EC of Xijing Hospital and obtain formal approval. Any amendments to the protocol must be approved again by the EC. The trial will be conducted in accordance with the protocol and the principles of the current version of the Declaration of Helsinki and Good Clinical Practice.

## 15.3 Patient Information and Informed Consent

Considering that the patients we plan to enroll do not have the ability to give informed consent, we obtain all consent from the patients' representatives after thoroughly informing them of the study design, possible risks and benefits, costs, privacy measures, and alternative treatments should the representatives choose not to participate in the trial. Additionally, the representatives have the right to withdraw from the trial at any stage, and there will be no punishment or loss of benefits as a result.

## 15.4 Participant privacy and confidentiality

All the research data collected in this study will be kept confidential at Xijing Hospital; in order to protect the patients' identities, uniform format numbers will be assigned to participants in place of their names in study documents. Any information that could potentially identify the patients will be removed from all subjects' information collected, ensuring that the information cannot be linked to a specific study subject.

## 15.5 Protocol amendments

Any important protocol modifications will be submitted for approval to the EC and updated in the clinical trials registry after discussion and agreement among study team members. If necessary, these protocol amendments will inform the legal representatives of patients who are already enrolled. All non-substantial amendments will be communicated to the EC within the Annual Safety Report.

# 16 PUBLICATION AND DISSEMINATION POLICY

Upon completion of the study, the results of the present study can be communicated through abstracts presented at national or international conferences. Scientific papers will be written by the study team and submitted to peer-reviewed scientific journals. All authors must contribute substantially to the paper, including at least a detailed

critical review.

# 17 FUNDING

National Natural Science Foundation of China (82441054), Shaanxi Province Special Support Program for Leading Talents in Scientific and Technological Innovation (tzihjw), and Clinical Research Project of the Fourth Military Medical University (2023LC2314).

# 18 REFERENCES

(1) Edlow, B. L.; Claassen, J.; Schiff, N. D.; Greer, D. M. Recovery from Disorders of Consciousness: Mechanisms, Prognosis and Emerging Therapies. *Nat Rev Neurol* **2021**, *17* (3), 135–156. https://doi.org/10.1038/s41582-020-00428-x.

(2) Laureys, S.; Celesia, G. G.; Cohadon, F.; Lavrijsen, J.; León-Carrión, J.; Sannita, W. G.; et al. Unresponsive Wakefulness Syndrome: A New Name for the Vegetative State or Apallic Syndrome. *BMC Med* **2010**, *8*, 68. https://doi.org/10.1186/1741-7015-8-68.

(3) Giacino, J. T.; Ashwal, S.; Childs, N.; Cranford, R.; Jennett, B.; Katz, D. I.; et al. The Minimally Conscious State: Definition and Diagnostic Criteria. *Neurology* **2002**, *58* (3), 349–353. https://doi.org/10.1212/wnl.58.3.349.

(4) Fridman, E. A.; Schiff, N. D. Neuromodulation of the Conscious State Following Severe Brain Injuries. *Curr Opin Neurobiol* **2014**, *29*, 172–177. https://doi.org/10.1016/j.conb.2014.09.008.

(5) Giacino, J. T.; Whyte, J.; Bagiella, E.; Kalmar, K.; Childs, N.; Khademi, A.; et al. Placebo-Controlled Trial of Amantadine for Severe Traumatic Brain Injury. *N Engl J Med* **2012**, *366* (9), 819–826. https://doi.org/10.1056/NEJMoa1102609.

(6) Caliendo, E.; Lowder, R.; McLaughlin, M. J.; Watson, W. D.; Baum, K. T.; Blackwell, L. S.; et al. The Use of Methylphenidate During Inpatient Rehabilitation After Pediatric Traumatic Brain Injury: Population Characteristics and Prescribing Patterns. *J Head Trauma Rehabil* **2024**, *39* (3), E122–E131. https://doi.org/10.1097/HTR.0000000000000889.

(7) Krimchansky, B.-Z.; Keren, O.; Sazbon, L.; Groswasser, Z. Differential Time and Related Appearance of Signs, Indicating Improvement in the State of Consciousness in Vegetative State Traumatic Brain Injury (VS-TBI) Patients after Initiation of Dopamine Treatment. *Brain Inj* **2004**, *18* (11), 1099–1105. https://doi.org/10.1080/02699050310001646206.

(8) Passler, M. A.; Riggs, R. V. Positive Outcomes in Traumatic Brain Injury-Vegetative State: Patients Treated with Bromocriptine. *Arch Phys Med Rehabil* **2001**, *82* (3), 311–315. https://doi.org/10.1053/apmr.2001.20831.

(9) Fridman, E. A.; Krimchansky, B. Z.; Bonetto, M.; Galperin, T.; Gamzu, E. R.; Leiguarda, R. C.; et al. Continuous Subcutaneous Apomorphine for Severe Disorders of Consciousness after Traumatic Brain Injury. *Brain Inj* **2010**, *24* (4), 636–641. https://doi.org/10.3109/02699051003610433.

(10) Schiff, N. D. Central Thalamic Contributions to Arousal Regulation and Neurological Disorders of Consciousness. *Ann N Y Acad Sci* **2008**, *1129*, 105–118. https://doi.org/10.1196/annals.1417.029.

(11) Yang, Z.; Yue, T.; Zschorlich, V. R.; Li, D.; Wang, D.; Qi, F. Behavioral Effects of Repetitive Transcranial Magnetic Stimulation in Disorders of Consciousness: A Systematic Review and Meta-Analysis. *Brain Sci* **2023**, *13* (10). https://doi.org/10.3390/brainsci13101362.

(12) Thibaut, A.; Bruno, M.-A.; Ledoux, D.; Demertzi, A.; Laureys, S. tDCS in Patients with Disorders of Consciousness: Sham-Controlled Randomized Double-Blind Study. *Neurology* **2014**, *82* (13), 1112–1118. https://doi.org/10.1212/WNL.0000000000000260.

(13) Thibaut, A.; Fregni, F.; Estraneo, A.; Fiorenza, S.; Noe, E.; Llorens, R.; et al. Sham-Controlled Randomized Multicentre Trial of Transcranial Direct Current Stimulation for Prolonged Disorders of Consciousness. *Eur J Neurol* **2023**. https://doi.org/10.1111/ene.15974.

(14) Cain, J. A.; Spivak, N. M.; Coetzee, J. P.; Crone, J. S.; Johnson, M. A.; Lutkenhoff, E. S.; et al. Ultrasonic Thalamic Stimulation in Chronic Disorders of Consciousness. *Brain Stimul* **2021**, *14* (2), 301–303. https://doi.org/10.1016/j.brs.2021.01.008.

(15) Corazzol, M.; Lio, G.; Lefevre, A.; Deiana, G.; Tell, L.; André-Obadia, N.; et al. Restoring Consciousness with Vagus Nerve Stimulation. *Curr Biol* **2017**, *27* (18), R994–R996. https://doi.org/10.1016/j.cub.2017.07.060.

(16) Piedade, G. S.; Assumpcao de Monaco, B.; Guest, J. D.; Cordeiro, J. G. Review of Spinal Cord Stimulation for Disorders of Consciousness. *Curr Opin Neurol* **2023**, *36* (6), 507–515. https://doi.org/10.1097/WCO.0000000000001222.

(17) Klomjai, W.; Katz, R.; Lackmy-Vallée, A. Basic Principles of Transcranial Magnetic Stimulation (TMS) and Repetitive TMS (rTMS). *Ann Phys Rehabil Med* **2015**, *58* (4), 208–213. https://doi.org/10.1016/j.rehab.2015.05.005.

(18) Lefaucheur, J.-P.; Aleman, A.; Baeken, C.; Benninger, D. H.; Brunelin, J.; Di Lazzaro, V.; et al. Evidence-Based Guidelines on the Therapeutic Use of Repetitive Transcranial Magnetic Stimulation (rTMS): An Update (2014-2018). *Clin Neurophysiol* **2020**, *131* (2), 474–528. https://doi.org/10.1016/j.clinph.2019.11.002.

(19) O'Neal, C. M.; Schroeder, L. N.; Wells, A. A.; Chen, S.; Stephens, T. M.; Glenn, C. A.; et al. Patient Outcomes in Disorders of Consciousness Following Transcranial Magnetic Stimulation: A Systematic Review and Meta-Analysis of Individual Patient Data. *Front Neurol* **2021**, *12*, 694970. https://doi.org/10.3389/fneur.2021.694970.

(20) Chen, J.-M.; Chen, Q.-F.; Wang, Z.-Y.; Chen, Y.-J.; Zhang, N.-N.; Xu, J.-W.; et al. Influence of High-Frequency Repetitive Transcranial Magnetic Stimulation on Neurobehavioral and Electrophysiology in Patients with Disorders of Consciousness. *Neural Plast* **2022**, *2022*, 7195699. https://doi.org/10.1155/2022/7195699.

(21) Zhang, X.-H.; Han, P.; Zeng, Y.-Y.; Wang, Y.-L.; Lv, H.-L. The Clinical Effect of Repetitive Transcranial Magnetic Stimulation on the Disturbance of Consciousness in Patients in a Vegetative State. *Front Neurosci* **2021**, *15*, 647517. https://doi.org/10.3389/fnins.2021.647517.

(22) Fan, J.; Zhong, Y.; Wang, H.; Aierken, N.; He, R. Repetitive Transcranial Magnetic Stimulation Improves Consciousness in Some Patients with Disorders of Consciousness. *Clin Rehabil* **2022**, *36* (7), 916–925. https://doi.org/10.1177/02692155221089455.

(23) Shen, L.; Huang, Y.; Liao, Y.; Yin, X.; Huang, Y.; Ou, J.; et al. Effect of High-Frequency Repetitive Transcranial Magnetic Stimulation over M1 for Consciousness Recovery after Traumatic Brain Injury. *Brain Behav* **2023**, *13* (5), e2971. https://doi.org/10.1002/brb3.2971.

(24) Wu, M.; Wu, Y.; Yu, Y.; Gao, J.; Meng, F.; He, F.; et al. Effects of Theta Burst Stimulation of the Left Dorsolateral Prefrontal Cortex in Disorders of Consciousness. *Brain Stimul* **2018**, *11* (6), 1382–1384. https://doi.org/10.1016/j.brs.2018.07.055.

(25) Schiff, N. D. Recovery of Consciousness after Brain Injury: A Mesocircuit Hypothesis. *Trends Neurosci* **2010**, *33* (1), 1–9. https://doi.org/10.1016/j.tins.2009.11.002.

(26) Stoodley, C. J.; Schmahmann, J. D. Functional Topography in the Human Cerebellum: A Meta-Analysis of Neuroimaging Studies. *Neuroimage* **2009**, *44* (2), 489–501. https://doi.org/10.1016/j.neuroimage.2008.08.039.

(27) Zhu, J.; Chen, C.; Liu, X.; He, M.; Fang, Y.; Wang, L.; et al. Cerebellar Purkinje Cell Firing Promotes Conscious Recovery from Anesthesia State through Coordinating Neuronal Communications with Motor Cortex. *Theranostics* **2024**, *14* (2), 480–495. https://doi.org/10.7150/thno.89592.

(28) Ou, S.-Q.; Wei, P.-H.; Fan, X.-T.; Wang, Y.-H.; Meng, F.; Li, M.-Y.; et al.

Delineating the Decussating Dentato-Rubro-Thalamic Tract and Its Connections in Humans Using Diffusion Spectrum Imaging Techniques. *Cerebellum* **2022**, *21* (1), 101–115. https://doi.org/10.1007/s12311-021-01283-2.

(29) Rossi, S.; Hallett, M.; Rossini, P. M.; Pascual-Leone, A. Safety, Ethical Considerations, and Application Guidelines for the Use of Transcranial Magnetic Stimulation in Clinical Practice and Research. *Clin Neurophysiol* **2009**, *120* (12), 2008–2039. https://doi.org/10.1016/j.clinph.2009.08.016.

(30) Rossini, P. M.; Burke, D.; Chen, R.; Cohen, L. G.; Daskalakis, Z.; Di Iorio, R.; et al. Non-Invasive Electrical and Magnetic Stimulation of the Brain, Spinal Cord, Roots and Peripheral Nerves: Basic Principles and Procedures for Routine Clinical and Research Application. An Updated Report from an I.F.C.N. Committee. *Clin Neurophysiol* **2015**, *126* (6), 1071–1107. https://doi.org/10.1016/j.clinph.2015.02.001.

(31) Vitello, M. M.; Rosenfelder, M. J.; Cardone, P.; Niimi, M.; Willacker, L.; Thibaut, A.; et al. A Protocol for a Multicenter Randomized and Personalized Controlled Trial Using rTMS in Patients with Disorders of Consciousness. *Front Neurol* **2023**, *14*, 1216468. https://doi.org/10.3389/fneur.2023.1216468.

(32) Lapitskaya, N.; Gosseries, O.; De Pasqua, V.; Pedersen, A. R.; Nielsen, J. F.; de Noordhout, A. M.; et al. Abnormal Corticospinal Excitability in Patients with Disorders of Consciousness. *Brain Stimul* **2013**, *6* (4), 590–597. https://doi.org/10.1016/j.brs.2013.01.002.

(33) Buckner, R. L. The Cerebellum and Cognitive Function: 25 Years of Insight from Anatomy and Neuroimaging. *Neuron* **2013**, *80* (3), 807–815. https://doi.org/10.1016/j.neuron.2013.10.044.

(34) Park, M. T. M.; Pipitone, J.; Baer, L. H.; Winterburn, J. L.; Shah, Y.; Chavez, S.; et al. Derivation of High-Resolution MRI Atlases of the Human Cerebellum at 3T and Segmentation Using Multiple Automatically Generated Templates. *Neuroimage* **2014**, *95*, 217–231. https://doi.org/10.1016/j.neuroimage.2014.03.037.

(35) Stokes, M. G.; Chambers, C. D.; Gould, I. C.; Henderson, T. R.; Janko, N. E.; Allen, N. B.; et al. Simple Metric for Scaling Motor Threshold Based on Scalp-Cortex Distance: Application to Studies Using Transcranial Magnetic Stimulation. *J Neurophysiol* **2005**, *94* (6), 4520–4527. https://doi.org/10.1152/jn.00067.2005.

(36) Rossi, S.; Hallett, M.; Rossini, P. M.; Pascual-Leone, A. Safety, Ethical Considerations, and Application Guidelines for the Use of Transcranial Magnetic Stimulation in Clinical Practice and Research. *Clin Neurophysiol* **2009**, *120* (12), 2008–2039. https://doi.org/10.1016/j.clinph.2009.08.016.

(37) Giacino, J. T.; Kalmar, K.; Whyte, J. The JFK Coma Recovery Scale-Revised: Measurement Characteristics and Diagnostic Utility. *Arch Phys Med Rehabil* **2004**, *85* (12), 2020–2029. https://doi.org/10.1016/j.apmr.2004.02.033.

(38) Kondziella, D.; Bender, A.; Diserens, K.; van Erp, W.; Estraneo, A.; Formisano, R.; et al. European Academy of Neurology Guideline on the Diagnosis of Coma and Other Disorders of Consciousness. *Eur J Neurol* **2020**, *27* (5), 741–756. https://doi.org/10.1111/ene.14151.

(39) Thibaut, A.; Panda, R.; Annen, J.; Sanz, L. R. D.; Naccache, L.; Martial, C.; et al. Preservation of Brain Activity in Unresponsive Patients Identifies MCS Star. *Ann Neurol* **2021**, *90* (1), 89–100. https://doi.org/10.1002/ana.26095.

(40) Petzinka, V. N.; Endisch, C.; Streitberger, K. J.; Salih, F.; Ploner, C. J.; Storm, C.; et al. Unresponsive Wakefulness or Coma after Cardiac Arrest-A Long-Term Follow-up Study. *Resuscitation* **2018**, *131*, 121–127. https://doi.org/10.1016/j.resuscitation.2018.07.007.

(41) Egbebike, J.; Shen, Q.; Doyle, K.; Der-Nigoghossian, C. A.; Panicker, L.; Gonzales, I. J.; et al. Cognitive-Motor Dissociation and Time to Functional Recovery in Patients with Acute Brain Injury in the USA: A Prospective Observational Cohort Study. *Lancet Neurol* **2022**, *21* (8), 704–713. https://doi.org/10.1016/S1474-4422(22)00212-5.

(42) McCrea, M. A.; Giacino, J. T.; Barber, J.; Temkin, N. R.; Nelson, L. D.; Levin, H. S.; et al. Functional Outcomes Over the First Year After Moderate to Severe Traumatic Brain Injury in the Prospective, Longitudinal TRACK-TBI Study. *JAMA Neurol* **2021**, *78* (8), 982–992. https://doi.org/10.1001/jamaneurol.2021.2043.

(43) Welch, P. D. The Use of Fast Fourier Transform for the Estimation of Power Spectra: A Method Based on Time Averaging over Short, Modified Periodograms. *IEEE Trans. Audio & Electroacoust.* **1967**, *15* (2), 70–73. https://doi.org/10.1109/TAU.1967.1161901.

(44) Forgacs, P. B.; Frey, H.-P.; Velazquez, A.; Thompson, S.; Brodie, D.; Moitra, V.; et al. Dynamic Regimes of Neocortical Activity Linked to Corticothalamic Integrity Correlate with Outcomes in Acute Anoxic Brain Injury after Cardiac Arrest. *Ann Clin Transl Neurol* **2017**, *4* (2), 119–129. https://doi.org/10.1002/acn3.385.

(45) He, R. H.; Wang, H. J.; Zhou, Z.; Fan, J. Z.; Zhang, S. Q.; Zhong, Y. H. The Influence of High-Frequency Repetitive Transcranial Magnetic Stimulation on Endogenous Estrogen in Patients with Disorders of Consciousness. *Brain Stimul* **2021**, *14* (3), 461–466. https://doi.org/10.1016/j.brs.2021.02.014.

(46) Cavinato, M.; Genna, C.; Formaggio, E.; Gregorio, C.; Storti, S. F.; Manganotti, P.; et al. Behavioural and Electrophysiological Effects of tDCS to Prefrontal Cortex in Patients with Disorders of Consciousness. *Clin Neurophysiol* **2019**, *130* (2), 231–238. https://doi.org/10.1016/j.clinph.2018.10.018.

(47) Liu, P.; Gao, J.; Pan, S.; Meng, F.; Pan, G.; Li, J.; et al. Effects of High-Frequency Repetitive Transcranial Magnetic Stimulation on Cerebral Hemodynamics in Patients with Disorders of Consciousness: A Sham-Controlled Study. *Eur Neurol* **2016**, *76* (1–2), 1–7. https://doi.org/10.1159/000447325.

(48) Putt, M.; Chinchilli, V. M. A Mixed Effects Model for the Analysis of Repeated Measures Cross-over Studies. *Stat Med* **1999**, *18* (22), 3037–3058. https://doi.org/10.1002/(sici)1097-0258(19991130)18:22<3037::aid-sim243>3.0.co;2-7.

(49) Darlow, B.; Stanley, J.; Dean, S.; Abbott, J. H.; Garrett, S.; Wilson, R.; et al. The Fear Reduction Exercised Early (FREE) Approach to Management of Low Back Pain in General Practice: A Pragmatic Cluster-Randomised Controlled Trial. *PLoS Med* **2019**, *16* (9), e1002897. https://doi.org/10.1371/journal.pmed.1002897.

(50) Nelson, E. J.; Khan, A. I.; Keita, A. M.; Brintz, B. J.; Keita, Y.; Sanogo, D.; et al. Improving Antibiotic Stewardship for Diarrheal Disease With Probability-Based Electronic Clinical Decision Support: A Randomized Crossover Trial. *JAMA Pediatr* **2022**, *176* (10), 973–979. https://doi.org/10.1001/jamapediatrics.2022.2535.

(51) Taylor, J. E.; Rousselet, G. A.; Scheepers, C.; Sereno, S. C. Rating Norms Should Be Calculated from Cumulative Link Mixed Effects Models. *Behav Res Methods* **2023**, *55* (5), 2175–2196. https://doi.org/10.3758/s13428-022-01814-7.

(52) Glickman, M. E.; Rao, S. R.; Schultz, M. R. False Discovery Rate Control Is a Recommended Alternative to Bonferroni-Type Adjustments in Health Studies. *J Clin Epidemiol* **2014**, *67* (8), 850–857. https://doi.org/10.1016/j.jclinepi.2014.03.012.

# 19 APPENDICES

| **JFK COMA RECOVERY SCALE ©2004 Record Form** | | | | | | | | | | | | | | | | | | | | | | | | | | | | |
|---|---|---|---|---|---|---|---|---|---|---|---|---|---|---|---|---|---|---|---|---|---|---|---|---|---|---|---|---|
| **Patient: Date:** | | | | | | | | | | | | | | | | | | | | | | | | | | | | |
| **Study period** | | | | | | | | | | | | | | | | | | | | | | | | | | | | |
| **AUDITORY FUNCTION SCALE** | | | | | | | | | | | | | | | | | | | | | | | | | | | | |
| 4 – Consistent Movement to Command* | | | | | | | | | | | | | | | | | | | | | | | | | | | | |
| 3 – Reproducible Movement to Command* | | | | | | | | | | | | | | | | | | | | | | | | | | | | |
| 2 – Localization to Sound | | | | | | | | | | | | | | | | | | | | | | | | | | | | |
| 1 – Auditory Startle | | | | | | | | | | | | | | | | | | | | | | | | | | | | |
| 0 – None | | | | | | | | | | | | | | | | | | | | | | | | | | | | |
| **VISUAL FUNCTION SCALE** | | | | | | | | | | | | | | | | | | | | | | | | | | | | |
| 5 – Object Recognition* | | | | | | | | | | | | | | | | | | | | | | | | | | | | |
| 4 – Object localization: Reaching* | | | | | | | | | | | | | | | | | | | | | | | | | | | | |
| 3 – Visual Pursuit* | | | | | | | | | | | | | | | | | | | | | | | | | | | | |
| 2 – Fixation* | | | | | | | | | | | | | | | | | | | | | | | | | | | | |
| 1 – Visual Startle | | | | | | | | | | | | | | | | | | | | | | | | | | | | |
| 0 – None | | | | | | | | | | | | | | | | | | | | | | | | | | | | |
| **MOTOR FUNCTION SCALE** | | | | | | | | | | | | | | | | | | | | | | | | | | | | |
| 6 – Functional Object Use† | | | | | | | | | | | | | | | | | | | | | | | | | | | | |
| 5 – Automatic Motor Response* | | | | | | | | | | | | | | | | | | | | | | | | | | | | |
| 4 – Object Manipulation* | | | | | | | | | | | | | | | | | | | | | | | | | | | | |
| 3 – Localisation to Noxious Stimulation* | | | | | | | | | | | | | | | | | | | | | | | | | | | | |
| 2 – Flexion Withdrawal | | | | | | | | | | | | | | | | | | | | | | | | | | | | |
| 1 – Abnormal Posturing | | | | | | | | | | | | | | | | | | | | | | | | | | | | |
| 0 – None | | | | | | | | | | | | | | | | | | | | | | | | | | | | |
| **OROMOTOR/VERBAL FUNCTION SCALE** | | | | | | | | | | | | | | | | | | | | | | | | | | | | |
| 3 – Intelligible Verbalization* | | | | | | | | | | | | | | | | | | | | | | | | | | | | |
| 2 – Vocalization/Oral Movement | | | | | | | | | | | | | | | | | | | | | | | | | | | | |
| 1 – Oral Reflexive Movement | | | | | | | | | | | | | | | | | | | | | | | | | | | | |
| 0 – None | | | | | | | | | | | | | | | | | | | | | | | | | | | | |
| **COMMUNICATION SCALE** | | | | | | | | | | | | | | | | | | | | | | | | | | | | |
| 2 – Functional: Accurate† | | | | | | | | | | | | | | | | | | | | | | | | | | | | |
| 1 – Non-functional: Intentional* | | | | | | | | | | | | | | | | | | | | | | | | | | | | |
| 0 – None | | | | | | | | | | | | | | | | | | | | | | | | | | | | |
| **AROUSAL SCALE** | | | | | | | | | | | | | | | | | | | | | | | | | | | | |
| 3 – Attention* | | | | | | | | | | | | | | | | | | | | | | | | | | | | |
| 2 – Eye Opening w/o Stimulation | | | | | | | | | | | | | | | | | | | | | | | | | | | | |
| 1 – Eye Opening with Stimulation | | | | | | | | | | | | | | | | | | | | | | | | | | | | |
| 0 – Unarousable | | | | | | | | | | | | | | | | | | | | | | | | | | | | |
| **TOTAL SCORE** | | | | | | | | | | | | | | | | | | | | | | | | | | | | |

Abbreviation: w/o, without.

*Denotes MCS.

†Denotes emergence from MCS.

## Structured Telephone Questionnaire based on CRS-R

| Subscale | Question | |
|---|---|---|
| **Auditory** | # | Has the patient at any point been able to follow instructions (e.g. to fix a certain object or deliberately move a part of the body)? |
| | • | Did you ever detect eye opening or movements of the head in response to direct speech? |
| | • | Did you ever detect an eyelid movement, a blink or any other reaction in response to direct speech? |
| **Visual** | #/* | Upon instruction, is the patient able to touch a given object or to move a limb towards this object? |
| | * | Is the patient able to fix a given object and to track this object when it is moved? |
| | * | Is the patient able to fix any object or person with her or his eyes (e.g. you)? |
| **Motor** | † | Upon instruction, is the patient able to use common objects (e.g. a comb, a cup or a toothbrush)? |
| | * | Did you ever observe any spontaneous or automatic movements by the patient? If so, describe the situation(s). |
| | */• | Have you ever detected any reaction to pressure, pain or passive movement of the patient's body? |
| **Verbal/ Oromotor** | # | Has the patient at any point shown a reaction with understandable words or sentences to questions (e.g. "what is your name?") |
| **Communication** | †/# | Between the patient and her or his environment, has there been any type of verbal or non-verbal communication since hospital discharge? If so, describe the situation. |
| **Arousal** | • | Is the patient able to open her or his eyes in response or any other interaction of the environment? If so, is the patient able to keep the eyes open? |

†: EMCS (emergence from minimally conscious state)

#: MCS+ (minimally conscious state plus)

*: MCS- (minimally conscious state minus)

•: UWS (unresponsive wakefulness syndrome)

# Summary of protocol amendments

| Number | Page | Section | Version 1.0 | Version 2.0 |
|---|---|---|---|---|
| 1 | 12 | 6.1 | **Inclusion Criteria:**<br>• VS/UWS or MCS according to at least two CRS-R assessments<br>• Age ⩾18 years<br>• Written informed consent obtained from the legal surrogate<br>**Exclusion Criteria:**<br>• Patients in coma<br>• Brain injury <1 week<br>• Presence of metallic hardware in close contact with the discharging coil (such as cochlear implants, an Internal Pulse Generator or medication pumps)<br>• Patients with high risks according to standard questionnaire to screen transcranial magnetic stimulation (TMS) candidates | **Inclusion Criteria:**<br>• VS/UWS or MCS according to at least two CRS-R assessments<br>• Age ⩾18 years<br>• Time since brain injury between 15 days and 1 year<br>• Written informed consent obtained from the legal surrogate<br>**Exclusion Criteria:**<br>• Brain injury due to unknown etiology<br>• History of mental or psychiatric disorder prior to the brain injury<br>• Uncontrolled seizures or status epilepticus<br>• Unstable or deteriorating medical condition<br>• Contraindications to MRI<br>• Skull defect<br>• Contraindications to TMS |
| 2 | 12 | 7.1 | Bilateral RMTs are assessed prior to the initial administration of either active or sham stimulation on the same day, using single pulses delivered via an eight-figure coil positioned over the corresponding hemisphere of the patient's right and left hands, respectively. This is reported by the lowest intensity that elicits motor evoked potential ≥ 50 μV in at least 5 of 10 consecutive | Bilateral RMTs are assessed prior to the initial administration of either active or sham stimulation on the same day, using single pulses delivered via an eight-figure coil positioned over the corresponding hemisphere of the patient's right and left hands, respectively. This is indicated by a visually detectable twitch in the abductor pollicis brevis muscle. |

| | | | | |
|---|---|---|---|---|
| | | | stimulations. | |
| 3 | 13 | 8.1 | **Level of consciousness:** Two qualified and independent neurologists who are blinded to the patient allocation evaluate the CRS-R scores. The CRS-R is subdivided into six subscales, including auditory, visual, motor, oromotor/verbal, communication, and arousal, for a total of 23 points to assess both the conscious awareness and arousal aspects of DoC patients. Diagnoses of VS/UWS and MCS (minus or plus) are determined based on the presence of specific items during subscale assessments. The baseline consciousness is determined by the highest score obtained from at least two CRS-R assessments during enrollment. | **Level of consciousness:** Two qualified and independent neurologists who are blinded to the patient allocation evaluate the CRS-R scores. The CRS-R is subdivided into six subscales, including auditory, visual, motor, oromotor/verbal, communication, and arousal, for a total of 23 points to assess both the conscious awareness and arousal aspects of DoC patients. Diagnoses of VS/UWS and MCS (minus or plus) are determined based on the presence of specific items during subscale assessments. The baseline consciousness is determined by the highest score obtained from at least two CRS-R assessments during enrollment. The CRS-R assessment during the follow-up phase will be evaluated via structured telephone interviews, either with patients themselves or close relatives. |
| 4 | 14 | 8.2 | **Functional outcome assessment:** GOS-E is utilized to evaluate the functional outcome 3 and 6 months after treatment, with a score of ≥ 4 indicating a favorable outcome and < 4 indicating an unfavorable outcome. It will be conducted by another trained and independent evaluator through structured phone interviews. | **Definition of consciousness improvement and unimprovement:** According to the 6-month consciousness, patients will be categorized into the improved consciousness group if they transition from UWS to MCS or emergence from MCS (EMCS), from MCS minus to MCS plus or EMCS, and from MCS plus to EMCS. Patients with unimproved consciousness are defined as those with a reduced or unchanged |

| | | | | |
|---|---|---|---|---|
| | | | | level of consciousness at six months. Patients with unimproved consciousness are defined as those with a reduced or unchanged level of consciousness. If patients die during the follow-up period, their consciousness will be evaluated based on their level of consciousness before death. For patients who die between 3 and 6 months after treatment, their pre-death consciousness will be assessed based on their condition at 3 months post-treatment. For those who die within 3 months of treatment, their pre-death consciousness will be evaluated according to their consciousness at the time of discharge. |
| 5 | 14 | 8.3 | **EEG recording and analysis** | **Functional outcome assessment:** GOS-E is utilized to evaluate the functional outcome 3 and 6 months after treatment, with a score of ≥ 4 indicating a favorable outcome and < 4 indicating an unfavorable outcome. It will be conducted by another trained and independent evaluator through structured phone interviews. |
| 6 | 15 | 8.4.4 | | **Definition of EEG responsiveness to cerebellar iTBS:** The EEG responsiveness to cerebellar iTBS is defined as patients who exhibited elevated levels of "ABCD" EEG patterns compared to the baseline after either the first or fifth session of iTBS stimulation. |
| 7 | 18 | 13.2 | Continuous variables will be expressed as mean ± standard | Continuous variables will be expressed as mean ± standard |

| | | | | |
|---|---|---|---|---|
| | | | deviation (SD) or median (interquartile range, IQR), and categorical variables will be expressed as percentages. Baseline continuous variables will be analyzed using Student's t-test for normal distribution and Mann - Whitney U test for skewed distribution. Categorical variables will be analyzed using χ2 test analysis and Fisher's exact tests, when appropriate.<br>The between-group differences in the change of CRS-R total and subscale scores after the fifth treatment sessions will be analyzed using a linear mixed model (LMM) with fixed effects (sequence, period, time since injury, etiology, and age) and random effects (subjects for repeated measurements).[5–7]<br>The effects of cerebellar iTBS on CRS-R total scores will be further analyzed in subgroups of patients according to their baseline characteristics, i.e., VS/UWS and MCS, as well as anoxia etiology and non-anoxia | deviation (SD) or median (interquartile range, IQR), and categorical variables will be expressed as percentages. Baseline continuous variables will be analyzed using Student's t-test for normal distribution and Mann - Whitney U test for skewed distribution. Categorical variables will be analyzed using χ2 test analysis and Fisher's exact tests, when appropriate.<br>Besides the primary and secondary outcomes mentioned above, we will conduct a post-hoc analysis of the changes in CRS-R scores and EEG after the first treatment session. At three- and six-months post-treatment, the consciousness of patients will be additionally assessed based-on CRS-R via telephone interview.<br>The between-group differences in the change of CRS-R total and subscale scores after the first and fifth treatment sessions will be analyzed using a linear mixed model (LMM) with fixed effects (sequence, period, time since injury, etiology, and age) and random effects (subjects for repeated measurements).[46–48]<br>The effects of cerebellar iTBS |

| | | | | |
|---|---|---|---|---|
| | | | etiology.<br>The between-group differences in PSD after the fifth treatment session will also be analyzed via LMM, incorporating baseline PSD as a fixed effect alongside other fixed and random variables as above.<br>The cumulative link mixed effects model (CLMM), which incorporated fixed and random effects as those of LMM (fixed effects: sequence, period, time since injury, etiology, and age; random effects: subjects for repeated measurements) and added baseline "ABCD" EEG patterns as fixed effects, will be used to analyze the between-group differences in "ABCD" EEG patterns after the fifth treatments.[8–10]<br>The analysis will be conducted in the intention-to-treat (ITT) populations and repeated in the per-protocol (PP) populations to test the robustness. For the ITT population, missing data will be imputed using the last observation carried forward method. EEG analyses wil be | on CRS-R total scores will be further analyzed in subgroups of patients according to their baseline characteristics, i.e., VS/UWS and MCS, as well as anoxia etiology and non-anoxia etiology.<br>The between-group differences in PSD after the first and fifth treatment sessions will also be analyzed via LMM, incorporating baseline PSD as a fixed effect alongside other fixed and random variables as above.<br>The cumulative link mixed effects model (CLMM), which incorporated fixed and random effects as those of LMM (fixed effects: sequence, period, time since injury, etiology, and age; random effects: subjects for repeated measurements) and added baseline "ABCD" EEG patterns as fixed effects, will be used to analyze the between-group differences in "ABCD" EEG patterns after the first and fifth treatments.[49–51]We will use the ordinal logistic regression to explore the relationships between the baseline "ABCD" EEG patterns and the functional outcomes and recovery of consciousness six months post-treatment. To |

| | | | | |
|---|---|---|---|---|
| | | | performed in patients with valid EEG data, and adjustments for multiple comparisons will be conducted using the false discovery rate method.[44] Two-sided P-values < 0.05 were considered statistically significant. All statistical analyses will be performed using PASS 20.0 (NCSS, LLC, Kaysville, UT, USA), R version 4.3.0 and SPSS version 26 (SPSS Inc., Chicago, IL, USA). | investigate the relationships between EEG responsiveness and six-month outcomes, we will use the generalized linear model (GLM), with adjustments made for baseline characteristics and time period.<br>The analysis will be conducted in the intention-to-treat (ITT) populations and repeated in the per-protocol (PP) populations to test the robustness. For the ITT population, missing data will be imputed using the last observation carried forward method. EEG analyses wil be performed in patients with valid EEG data, and adjustments for multiple comparisons will be conducted using the false discovery rate method.[52] Two-sided P-values < 0.05 were considered statistically significant. All statistical analyses will be performed using PASS 20.0 (NCSS, LLC, Kaysville, UT, USA), R version 4.3.0 and SPSS version 26 (SPSS Inc., Chicago, IL, USA). |

ClinicalTrials registration: NCT05558930

Effect of Cerebellar Stimulation on the Awareness Recovery in Disorders of Consciousness: A randomized, controlled, double-blind, cross-over trial

# STATISTICAL ANALYSIS PLAN(Original)

Research site: Xijing Hospital

Principle Investigator: Wen Jiang

Responsible department: Department of Neurology

Contact: +86-29-84771319

Version 1.0-March 2022

# Table of Contents

ABBREVIATION ....................................................................................64
1 INTRODUCTION ....................................................................................65
2 STUDY OBJECTIVES AND OUTCOMES ....................................................................................65
2.1 Study objectives ....................................................................................65
2.1.1 Primary objective(s) ....................................................................................65
2.1.2 Secondary objective(s) ....................................................................................65
2.2 Study outcomes ....................................................................................65
2.2.1 Primary outcome(s) ....................................................................................65
2.2.2 Secondary outcome(s) ....................................................................................65
2.2.3 Safety outcome(s) ....................................................................................65
3 STUDY DESIGN ....................................................................................66
4 DETERMINATION OF SAMPLE SIZE ....................................................................................66
4.1 Analysis populations ....................................................................................66
5 MISSING DATA ....................................................................................66
6 EFFICACY ANALYSIS ....................................................................................67
7 SAFETY ANALYSIS ....................................................................................67
8 REFERENCES ....................................................................................67

# ABBREVIATION

| | |
|---|---|
| AEs | adverse events |
| CLMM | cumulative link mixed effects model |
| CRS-R | Coma Recovery Scale-Revised |
| DOC | disorders of consciousness |
| EEG | electroencephalography |
| GOS-E | Glasgow Outcome Scale-Extended |
| IQR | interquartile range |
| iTBS | serious adverse events |
| ITT | adverse events |
| LMM | linear mixed model |
| LOCF | last observation carried forward |
| MCS | minimally conscious state |
| MRI | magnetic resonance imaging |
| PP | per-protocol |
| PSD | power spectral density |
| SAE | serious adverse event |
| SAEs | serious adverse events |
| SAP | statistical analysis plan |
| SD | standard deviation |
| TMS | transcranial magnetic stimulation |
| UWS | unresponsive wakefulness syndrome |
| VS | vegetative state |

# 1 INTRODUCTION

This statistical analysis plan (SAP) details the planned statistical analyses for the "Effect of Cerebellar Stimulation on the Awareness Recovery in Disorders of Consciousness: A randomized, controlled, double-blind, cross-over trial." It is based on the protocol, as well as any subsequent amendments. The SAP should be read together with the protocol.

# 2 STUDY OBJECTIVES AND OUTCOMES

## 2.1 Study objectives

### 2.1.1 Primary objective(s)

To evaluate the effect of 5-session bilateral cerebellar iTBS stimulation on promoting consciousness recovery in patients with DoC.

### 2.1.2 Secondary objective(s)

- To evaluate the effects of cerebellar iTBS on EEG after five treatment sessions.
- To assess the functional outcomes of all the participants at 3 months and 6 months after enrolment.

## 2.2 Study outcomes

### 2.2.1 Primary outcome(s)

- Difference in the change of CRS-R total score between the iTBS and sham stimulation groups after five treatment sessions.

### 2.2.2 Secondary outcome(s)

- The between-group difference in the change of CRS-R subscale scores after five treatment sessions.
- The between-group difference in the "ABCD" EEG patterns and PSD after five treatment sessions.

### 2.2.3 Safety outcome(s)

Safety outcomes included adverse events, including intolerance to TMS, and severe adverse events. The common adverse effects of TMS include dizziness, headache, tinnitus, and tingling or burning sensation on the scalp, which usually ease

spontaneously without symptomatic treatment. Any uncomfortable condition due to the iTBS stimulation and needs to be eased by down-regulating stimulus intensity or discontinuation of stimulation are seen as intolerance. Rare adverse effect of TMS includes seizures, for which physicians will administer appropriate symptomatic treatment.

A Serious Adverse Event (SAE) is defined as any event that:

- requires inpatient treatment not envisioned in the protocol or extends a current hospital stay;
- results in permanent or significant incapacity or disability;
- is life-threatening or results in death;
- causes a congenital anomaly or birth defect.

# 3 STUDY DESIGN

This will be a randomized, double-blind, crossover trial. Patients will be randomly assigned to the iTBS stimulation-first group or the sham stimulation-first group after the 5-day baseline assessments. During each treatment session, patients will receive either active stimulation or sham stimulation once daily for 5 consecutive days. The two treatment sessions are separated by a 5-day washout period. Demographic, MRI, and clinical characteristics of the patients will be collected at enrolment. The CRS-R score will be assessed before and after every session of active and sham stimulation within 2 hours. 32-channel EEG will be performed 20 minutes before and after the first and after the fifth treatment sessions within 30 minutes. The analyses will compare the effects of iTBS and sham stimulation on the CRS-R score and EEG and assess the functional outcomes of all the participants 3 months and 6 months after enrolment.

# 4 DETERMINATION OF SAMPLE SIZE

We determined that enrolling 44 patients would provide 90% power to detect a mean difference of 1.2 points (standard deviation [SD] = 1.5) in CRS-R changes between the active and sham stimulation groups, using a two-sided α of 0.05 and accounting for a 15% dropout rate. The anticipated 1.2 ± 1.5-point difference was conservatively estimated based on previous studies.[1–4]

## 4.1 Analysis populations

Analyses of primary outcomes will be conducted in the ITT population and repeated in the PP population. The ITT population will include all patients who are randomized and the PP population will include all subjects who are randomized and do not deviate from the treatment procedure.

# 5 MISSING DATA

For the ITT population, missing data are imputed using the LOCF method. The patients who drop out before the cessation of the last intervention will be excluded from the PP analyses.

# 6 EFFICACY ANALYSIS

Continuous variables will be expressed as mean ± standard deviation (SD) or median (interquartile range, IQR), and categorical variables will be expressed as percentages. Baseline continuous variables will be analyzed using Student's t-test for normal distribution and Mann - Whitney U test for skewed distribution. Categorical variables will be analyzed using $\chi 2$ test analysis and Fisher's exact tests, when appropriate.

The between-group differences in the change of CRS-R total and subscale scores after the fifth treatment sessions will be analyzed using a linear mixed model (LMM) with fixed effects (sequence, period, time since injury, etiology, and age) and random effects (subjects for repeated measurements).[5–7]

The effects of cerebellar iTBS on CRS-R total scores will be further analyzed in subgroups of patients according to their baseline characteristics, i.e., VS/UWS and MCS, as well as anoxia etiology and non-anoxia etiology.

The between-group differences in PSD after the fifth treatment session will also be analyzed via LMM, incorporating baseline PSD as a fixed effect alongside other fixed and random variables as above.

The cumulative link mixed effects model (CLMM), which incorporated fixed and random effects as those of LMM (fixed effects: sequence, period, time since injury, etiology, and age; random effects: subjects for repeated measurements) and added baseline "ABCD" EEG patterns as fixed effects, will be used to analyze the between-group differences in "ABCD" EEG patterns after the fifth treatments.[8–10]

The analysis will be conducted in the intention-to-treat (ITT) populations and repeated in the per-protocol (PP) populations to test the robustness. For the ITT population, missing data will be imputed using the last observation carried forward method. EEG analyses will be performed in patients with valid EEG data, and adjustments for multiple comparisons will be conducted using the false discovery rate method.[11] Two-sided P-values < 0.05 were considered statistically significant. All statistical analyses will be performed using PASS 20.0 (NCSS, LLC, Kaysville, UT, USA), R version 4.3.0 and SPSS version 26 (SPSS Inc., Chicago, IL, USA).

# 7 SAFETY ANALYSIS

The primary analysis will be the ITT principle between the assigned treatment groups. The proportion of SAEs between the two groups will be analyzed via generalized estimating equations. $P < 0.05$ will be statistically significant. Statistical analyses will be performed using the R software (version R4.3.0)

# 8 REFERENCES

(1) Thibaut, A.; Bruno, M.-A.; Ledoux, D.; Demertzi, A.; Laureys, S. tDCS in Patients with Disorders of Consciousness: Sham-Controlled Randomized Double-Blind Study. *Neurology* **2014**, *82* (13), 1112–1118. https://doi.org/10.1212/WNL.0000000000000260.

(2) Shen, L.; Huang, Y.; Liao, Y.; Yin, X.; Huang, Y.; Ou, J.; et al. Effect of High-

Frequency Repetitive Transcranial Magnetic Stimulation over M1 for Consciousness Recovery after Traumatic Brain Injury. *Brain Behav* **2023**, *13* (5), e2971. https://doi.org/10.1002/brb3.2971.

(3) He, R. H.; Wang, H. J.; Zhou, Z.; Fan, J. Z.; Zhang, S. Q.; Zhong, Y. H. The Influence of High-Frequency Repetitive Transcranial Magnetic Stimulation on Endogenous Estrogen in Patients with Disorders of Consciousness. *Brain Stimul* **2021**, *14* (3), 461–466. https://doi.org/10.1016/j.brs.2021.02.014.

(4) Fan, J.; Zhong, Y.; Wang, H.; Aierken, N.; He, R. Repetitive Transcranial Magnetic Stimulation Improves Consciousness in Some Patients with Disorders of Consciousness. *Clin Rehabil* **2022**, *36* (7), 916–925. https://doi.org/10.1177/02692155221089455.

(5) Cavinato, M.; Genna, C.; Formaggio, E.; Gregorio, C.; Storti, S. F.; Manganotti, P.; et al. Behavioural and Electrophysiological Effects of tDCS to Prefrontal Cortex in Patients with Disorders of Consciousness. *Clin Neurophysiol* **2019**, *130* (2), 231–238. https://doi.org/10.1016/j.clinph.2018.10.018.

(6) Liu, P.; Gao, J.; Pan, S.; Meng, F.; Pan, G.; Li, J.; et al. Effects of High-Frequency Repetitive Transcranial Magnetic Stimulation on Cerebral Hemodynamics in Patients with Disorders of Consciousness: A Sham-Controlled Study. *Eur Neurol* **2016**, *76* (1–2), 1–7. https://doi.org/10.1159/000447325.

(7) Putt, M.; Chinchilli, V. M. A Mixed Effects Model for the Analysis of Repeated Measures Cross-over Studies. *Stat Med* **1999**, *18* (22), 3037–3058. https://doi.org/10.1002/(sici)1097-0258(19991130)18:22<3037::aid-sim243>3.0.co;2-7.

(8) Darlow, B.; Stanley, J.; Dean, S.; Abbott, J. H.; Garrett, S.; Wilson, R.; et al. The Fear Reduction Exercised Early (FREE) Approach to Management of Low Back Pain in General Practice: A Pragmatic Cluster-Randomised Controlled Trial. *PLoS Med* **2019**, *16* (9), e1002897. https://doi.org/10.1371/journal.pmed.1002897.

(9) Nelson, E. J.; Khan, A. I.; Keita, A. M.; Brintz, B. J.; Keita, Y.; Sanogo, D.; et al. Improving Antibiotic Stewardship for Diarrheal Disease With Probability-Based Electronic Clinical Decision Support: A Randomized Crossover Trial. *JAMA Pediatr* **2022**, *176* (10), 973–979. https://doi.org/10.1001/jamapediatrics.2022.2535.

(10) Taylor, J. E.; Rousselet, G. A.; Scheepers, C.; Sereno, S. C. Rating Norms Should Be Calculated from Cumulative Link Mixed Effects Models. *Behav Res Methods* **2023**, *55* (5), 2175–2196. https://doi.org/10.3758/s13428-022-01814-7.

(11) Glickman, M. E.; Rao, S. R.; Schultz, M. R. False Discovery Rate Control Is a Recommended Alternative to Bonferroni-Type Adjustments in Health Studies. *J*

*Clin Epidemiol* **2014**, *67* (8), 850–857. https://doi.org/10.1016/j.jclinepi.2014.03.012.

ClinicalTrials registration: NCT05558930

Effect of Cerebellar Stimulation on the Awareness Recovery in Disorders of Consciousness: A randomized, controlled, double-blind, cross-over trial

# STATISTICAL ANALYSIS PLAN(Final)

Research site: Xijing Hospital

Principle Investigator: Wen Jiang

Responsible department: Department of Neurology

Contact: +86-29-84771319

Version 2.0- October 2022

## Table of Contents

ABBREVIATION ........ 72
1 INTRODUCTION ........ 73
2 STUDY OBJECTIVES AND OUTCOMES ........ 73
2.1 Study objectives ........ 73
2.1.1 Primary objective(s) ........ 73
2.1.2 Secondary objective(s) ........ 73
2.2 Study outcomes ........ 73
2.2.1 Primary outcome(s) ........ 73
2.2.2 Secondary outcome(s) ........ 73
2.2.3 Follow-up outcome (s) ........ 73
2.2.4 Safety outcome(s) ........ 74
3 STUDY DESIGN ........ 74
4 DETERMINATION OF SAMPLE SIZE ........ 74
4.1 Analysis populations ........ 74
5 MISSING DATA ........ 75
6 EFFICACY ANALYSIS ........ 75
7 SAFETY ANALYSIS ........ 76
8 REFERENCES ........ 76

# ABBREVIATION

| | |
|---|---|
| AEs | adverse events |
| CLMM | cumulative link mixed effects model |
| CRS-R | Coma Recovery Scale-Revised |
| DOC | disorders of consciousness |
| EEG | electroencephalography |
| GLM | generalized linear model |
| GOS-E | Glasgow Outcome Scale-Extended |
| IQR | interquartile range |
| iTBS | serious adverse events |
| ITT | adverse events |
| LMM | linear mixed model |
| LOCF | last observation carried forward |
| MCS | minimally conscious state |
| MRI | magnetic resonance imaging |
| PP | per-protocol |
| PSD | power spectral density |
| SAE | serious adverse event |
| SAEs | serious adverse events |
| SAP | statistical analysis plan |
| SD | standard deviation |
| TMS | transcranial magnetic stimulation |
| UWS | unresponsive wakefulness syndrome |
| VS | vegetative state |

# 1 INTRODUCTION

This statistical analysis plan (SAP) details the planned statistical analyses for the "Effect of Cerebellar Stimulation on the Awareness Recovery in Disorders of Consciousness: A randomized, controlled, double-blind, cross-over trial." It is based on the protocol, as well as any subsequent amendments. The SAP should be read together with the protocol.

# 2 STUDY OBJECTIVES AND OUTCOMES

## 2.1 Study objectives

### 2.1.1 Primary objective(s)

To evaluate the effect of 5-session bilateral cerebellar iTBS stimulation on promoting consciousness recovery in patients with DoC.

### 2.1.2 Secondary objective(s)

- To evaluate the effects of cerebellar iTBS on EEG after five treatment sessions.
- To assess the functional outcomes of all the participants at 3 months and 6 months after enrolment.

## 2.2 Study outcomes

### 2.2.1 Primary outcome(s)

- Difference in the change of CRS-R total score between the iTBS and sham stimulation groups after five treatment sessions.

### 2.2.2 Secondary outcome(s)

- The between-group difference in the change of CRS-R subscale scores after five treatment sessions.

- The between-group difference in the "ABCD" EEG patterns and PSD after five treatment sessions.

### 2.2.3 Follow-up outcome (s)

- GOS-E scores at 3 months and 6 months after enrolment.

### 2.2.4 Safety outcome(s)

Safety outcomes included adverse events, including intolerance to TMS, and severe adverse events. The common adverse effects of TMS include dizziness, headache, tinnitus, and tingling or burning sensation on the scalp, which usually ease spontaneously without symptomatic treatment. Any uncomfortable condition due to the iTBS stimulation and needs to be eased by down-regulating stimulus intensity or discontinuation of stimulation are seen as intolerance. Rare adverse effect of TMS includes seizures, for which physicians will administer appropriate symptomatic treatment.

A Serious Adverse Event (SAE) is defined as any event that:

- requires inpatient treatment not envisioned in the protocol or extends a current hospital stay;
- results in permanent or significant incapacity or disability;
- is life-threatening or results in death;
- causes a congenital anomaly or birth defect.

# 3 STUDY DESIGN

This will be a randomized, double-blind, crossover trial. Patients will be randomly assigned to the iTBS stimulation-first group or the sham stimulation-first group after the 5-day baseline assessments. During each treatment session, patients will receive either active stimulation or sham stimulation once daily for 5 consecutive days. The two treatment sessions are separated by a 5-day washout period. Demographic, MRI, and clinical characteristics of the patients will be collected at enrolment. The CRS-R score will be assessed before and after every session of active and sham stimulation within 2 hours. 32-channel EEG will be performed 20 minutes before and after the first and after the fifth treatment sessions within 30 minutes. The analyses will compare the effects of iTBS and sham stimulation on the CRS-R score and EEG and assess the functional outcomes of all the participants 3 months and 6 months after enrolment.

# 4 DETERMINATION OF SAMPLE SIZE

We determined that enrolling 44 patients would provide 90% power to detect a mean difference of 1.2 points (standard deviation [SD] = 1.5) in CRS-R changes between the active and sham stimulation groups, using a two-sided α of 0.05 and accounting for a 15% dropout rate. The anticipated 1.2 ± 1.5-point difference was conservatively estimated based on previous studies.[1–4]

## 4.1 Analysis populations

Analyses of primary outcomes will be conducted in the ITT population and repeated in the PP population. The ITT population will include all patients who are randomized and the PP population will include all subjects who are randomized and do not deviate from the treatment procedure.

# 5 MISSING DATA

For the ITT population, missing data are imputed using the LOCF method. The patients who drop out before the cessation of the last intervention will be excluded from the PP analyses.

# 6 EFFICACY ANALYSIS

Continuous variables will be expressed as mean ± standard deviation (SD) or median (interquartile range, IQR), and categorical variables will be expressed as percentages. Baseline continuous variables will be analyzed using Student's t-test for normal distribution and Mann - Whitney U test for skewed distribution. Categorical variables will be analyzed using $\chi 2$ test analysis and Fisher's exact tests, when appropriate.

Besides the primary and secondary outcomes mentioned above, we will conduct a post-hoc analysis of the changes in CRS-R scores and EEG after the first treatment session. At three- and six-months post-treatment, the consciousness of patients will be additionally assessed based-on CRS-R via telephone interview.

The between-group differences in the change of CRS-R total and subscale scores after the first and fifth treatment sessions will be analyzed using a linear mixed model (LMM) with fixed effects (sequence, period, time since injury, etiology, and age) and random effects (subjects for repeated measurements).[5–7]

The effects of cerebellar iTBS on CRS-R total scores will be further analyzed in subgroups of patients according to their baseline characteristics, i.e., VS/UWS and MCS, as well as anoxia etiology and non-anoxia etiology.

The between-group differences in PSD after the first and fifth treatment sessions will also be analyzed via LMM, incorporating baseline PSD as a fixed effect alongside other fixed and random variables as above.

The cumulative link mixed effects model (CLMM), which incorporated fixed and random effects as those of LMM (fixed effects: sequence, period, time since injury, etiology, and age; random effects: subjects for repeated measurements) and added baseline "ABCD" EEG patterns as fixed effects, will be used to analyze the between-group differences in "ABCD" EEG patterns after the first and fifth treatments.[8–10]

We will use the ordinal logistic regression to explore the relationships between the baseline "ABCD" EEG patterns and the functional outcomes and recovery of consciousness six months post-treatment. To investigate the relationships between EEG responsiveness and six-month outcomes, we will use the generalized linear model (GLM), with adjustments made for baseline characteristics and time period.

The analysis will be conducted in the intention-to-treat (ITT) populations and repeated in the per-protocol (PP) populations to test the robustness. For the ITT population, missing data will be imputed using the last observation carried forward method. EEG analyses wil be performed in patients with valid EEG data, and adjustments for multiple comparisons will be conducted using the false discovery rate method.[11] Two-sided P-values $< 0.05$ were considered statistically significant. All statistical analyses will be performed using PASS 20.0 (NCSS, LLC, Kaysville, UT,

USA), R version 4.3.0 and SPSS version 26 (SPSS Inc., Chicago, IL, USA).

# 7 SAFETY ANALYSIS

The primary analysis will be the ITT principle between the assigned treatment groups. The proportion of SAEs between the two groups will be analyzed via generalized estimating equations. $P < 0.05$ will be statistically significant. Statistical analyses will be performed using the R software (version R4.3.0)

# 8 REFERENCES


(1) Thibaut, A.; Bruno, M.-A.; Ledoux, D.; Demertzi, A.; Laureys, S. tDCS in Patients with Disorders of Consciousness: Sham-Controlled Randomized Double-Blind Study. *Neurology* **2014**, *82* (13), 1112–1118. https://doi.org/10.1212/WNL.0000000000000260.

(2) Shen, L.; Huang, Y.; Liao, Y.; Yin, X.; Huang, Y.; Ou, J.; et al. Effect of High-Frequency Repetitive Transcranial Magnetic Stimulation over M1 for Consciousness Recovery after Traumatic Brain Injury. *Brain Behav* **2023**, *13* (5), e2971. https://doi.org/10.1002/brb3.2971.

(3) He, R. H.; Wang, H. J.; Zhou, Z.; Fan, J. Z.; Zhang, S. Q.; Zhong, Y. H. The Influence of High-Frequency Repetitive Transcranial Magnetic Stimulation on Endogenous Estrogen in Patients with Disorders of Consciousness. *Brain Stimul* **2021**, *14* (3), 461–466. https://doi.org/10.1016/j.brs.2021.02.014.

(4) Fan, J.; Zhong, Y.; Wang, H.; Aierken, N.; He, R. Repetitive Transcranial Magnetic Stimulation Improves Consciousness in Some Patients with Disorders of Consciousness. *Clin Rehabil* **2022**, *36* (7), 916–925. https://doi.org/10.1177/02692155221089455.

(5) Cavinato, M.; Genna, C.; Formaggio, E.; Gregorio, C.; Storti, S. F.; Manganotti, P.; et al. Behavioural and Electrophysiological Effects of tDCS to Prefrontal Cortex in Patients with Disorders of Consciousness. *Clin Neurophysiol* **2019**, *130* (2), 231–238. https://doi.org/10.1016/j.clinph.2018.10.018.

(6) Liu, P.; Gao, J.; Pan, S.; Meng, F.; Pan, G.; Li, J.; et al. Effects of High-Frequency Repetitive Transcranial Magnetic Stimulation on Cerebral Hemodynamics in Patients with Disorders of Consciousness: A Sham-Controlled Study. *Eur Neurol* **2016**, *76* (1–2), 1–7. https://doi.org/10.1159/000447325.

(7) Putt, M.; Chinchilli, V. M. A Mixed Effects Model for the Analysis of Repeated Measures Cross-over Studies. *Stat Med* **1999**, *18* (22), 3037–3058. https://doi.org/10.1002/(sici)1097-0258(19991130)18:22<3037::aid-sim243>3.0.co;2-7.

(8) Darlow, B.; Stanley, J.; Dean, S.; Abbott, J. H.; Garrett, S.; Wilson, R.; et al. The

Fear Reduction Exercised Early (FREE) Approach to Management of Low Back Pain in General Practice: A Pragmatic Cluster-Randomised Controlled Trial. *PLoS Med* **2019**, *16* (9), e1002897. https://doi.org/10.1371/journal.pmed.1002897.

(9) Nelson, E. J.; Khan, A. I.; Keita, A. M.; Brintz, B. J.; Keita, Y.; Sanogo, D.; et al. Improving Antibiotic Stewardship for Diarrheal Disease With Probability-Based Electronic Clinical Decision Support: A Randomized Crossover Trial. *JAMA Pediatr* **2022**, *176* (10), 973–979. https://doi.org/10.1001/jamapediatrics.2022.2535.

(10) Taylor, J. E.; Rousselet, G. A.; Scheepers, C.; Sereno, S. C. Rating Norms Should Be Calculated from Cumulative Link Mixed Effects Models. *Behav Res Methods* **2023**, *55* (5), 2175–2196. https://doi.org/10.3758/s13428-022-01814-7.

(11) Glickman, M. E.; Rao, S. R.; Schultz, M. R. False Discovery Rate Control Is a Recommended Alternative to Bonferroni-Type Adjustments in Health Studies. *J Clin Epidemiol* **2014**, *67* (8), 850–857. https://doi.org/10.1016/j.jclinepi.2014.03.012.

## Summary of statistical analysis plan amendments

| Number | Page | Section | Version 1.0 | Version 2.0 |
|---|---|---|---|---|
| 1 | 63 | 2.2 | 2.2.1 Primary outcome(s)<br>2.2.2 Secondary outcome(s)<br>2.2.3 Safety outcome(s) | 2.2.1 Primary outcome(s)<br>2.2.2 Secondary outcome(s)<br>2.2.3 Follow-up outcome (s)<br>GOS-E scores at 3 months and 6 months after enrolment.<br>2.2.4 Safety outcome(s) |
| 2 | 64 | 3 | The analyses will compare the effects of iTBS and sham stimulation on the CRS-R score and EEG indicators and assess the functional outcomes 3 months and 6 months after enrolment. | The analyses will compare the effects of iTBS and sham stimulation on the CRS-R score and EEG indicators and assess the functional outcomes and consciousness recovery 3 months and 6 months after enrolment. |
| 3 | 65 | 6 | Continuous variables will be expressed as mean ± standard deviation (SD) or median (interquartile range, IQR), and categorical variables will be expressed as percentages. Baseline continuous variables will be analyzed using Student's t-test for normal distribution and Mann - Whitney U test for skewed distribution. Categorical variables will be analyzed using χ2 test analysis and Fisher's exact tests, when appropriate.<br>The between-group differences in the change of | Continuous variables will be expressed as mean ± standard deviation (SD) or median (interquartile range, IQR), and categorical variables will be expressed as percentages. Baseline continuous variables will be analyzed using Student's t-test for normal distribution and Mann - Whitney U test for skewed distribution. Categorical variables will be analyzed using χ2 test analysis and Fisher's exact tests, when appropriate.<br>Besides the primary and secondary outcomes mentioned above, we will conduct a post-hoc analysis of the changes in CRS-R |

| | | | | |
|---|---|---|---|---|
| | | | CRS-R total and subscale scores after the fifth treatment sessions will be analyzed using a linear mixed model (LMM) with fixed effects (sequence, period, time since injury, etiology, and age) and random effects (subjects for repeated measurements).[5–7]<br>The effects of cerebellar iTBS on CRS-R total scores will be further analyzed in subgroups of patients according to their baseline characteristics, i.e., VS/UWS and MCS, as well as anoxia etiology and non-anoxia etiology.<br>The between-group differences in PSD after the fifth treatment session will also be analyzed via LMM, incorporating baseline PSD as a fixed effect alongside other fixed and random variables as above.<br>The cumulative link mixed effects model (CLMM), which incorporated fixed and random effects as those of LMM (fixed effects: sequence, period, time since injury, etiology, and age; random effects: subjects for | scores and EEG after the first treatment session. At three- and six-months post-treatment, the consciousness of patients will be additionally assessed based-on CRS-R via telephone interview.<br>The between-group differences in the change of CRS-R total and subscale scores after the first and fifth treatment sessions will be analyzed using a linear mixed model (LMM) with fixed effects (sequence, period, time since injury, etiology, and age) and random effects (subjects for repeated measurements).[5–7]<br>The effects of cerebellar iTBS on CRS-R total scores will be further analyzed in subgroups of patients according to their baseline characteristics, i.e., VS/UWS and MCS, as well as anoxia etiology and non-anoxia etiology.<br>The between-group differences in PSD after the first and fifth treatment sessions will also be analyzed via LMM, incorporating baseline PSD as a fixed effect alongside other fixed and random variables as above.<br>The cumulative link mixed effects model (CLMM), which |

| | | | | |
|---|---|---|---|---|
| | | | repeated measurements) and added baseline "ABCD" EEG patterns as fixed effects, will be used to analyze the between-group differences in "ABCD" EEG patterns after the fifth treatments.[8–10]<br>The analysis will be conducted in the intention-to-treat (ITT) populations and repeated in the per-protocol (PP) populations to test the robustness. For the ITT population, missing data will be imputed using the last observation carried forward method. EEG analyses will be performed in patients with valid EEG data, and adjustments for multiple comparisons will be conducted using the false discovery rate method.[11] Two-sided P-values < 0.05 were considered statistically significant. All statistical analyses will be performed using PASS 20.0 (NCSS, LLC, Kaysville, UT, USA), R version 4.3.0 and SPSS version 26 (SPSS Inc., Chicago, IL, USA). | incorporated fixed and random effects as those of LMM (fixed effects: sequence, period, time since injury, etiology, and age; random effects: subjects for repeated measurements) and added baseline "ABCD" EEG patterns as fixed effects, will be used to analyze the between-group differences in "ABCD" EEG patterns after the first and fifth treatments.[8–10]<br>We will use the ordinal logistic regression to explore the relationships between the baseline "ABCD" EEG patterns and the functional outcomes and recovery of consciousness six months post-treatment. To investigate the relationships between EEG responsiveness and six-month outcomes, we will use the generalized linear model (GLM), with adjustments made for baseline characteristics and time period.<br>The analysis will be conducted in the intention-to-treat (ITT) populations and repeated in the per-protocol (PP) populations to test the robustness. For the ITT population, missing data will be imputed using the last observation |

| | | | | |
|---|---|---|---|---|
| | | | | carried forward method. EEG analyses wil be performed in patients with valid EEG data, and adjustments for multiple comparisons will be conducted using the false discovery rate method.[11] Two-sided P-values < 0.05 were considered statistically significant. All statistical analyses will be performed using PASS 20.0 (NCSS, LLC, Kaysville, UT, USA), R version 4.3.0 and SPSS version 26 (SPSS Inc., Chicago, IL, USA). |
